# A Quantitative Evaluation of Thin Slice Sampling for Parent–Infant Interactions

**DOI:** 10.1007/s10919-022-00420-7

**Published:** 2023-01-19

**Authors:** Romana Burgess, Ilaria Costantini, Marc H. Bornstein, Amy Campbell, Miguel A. Cordero Vega, Iryna Culpin, Hayley Dingsdale, Rosalind M. John, Mari-Rose Kennedy, Hannah R. Tyson, Rebecca M. Pearson, Ian Nabney

**Affiliations:** 1 Digital Health Engineering Group, Faculty of Engineering, Merchant Venturers Building, University of Bristol, Bristol, UK; 2 Centre for Academic Mental Health, Population Health Sciences, Bristol Medical School, University of Bristol, Oakfield House, Bristol, UK; 3 Eunice Kennedy Shriver National Institute of Child Health and Human Development, Bethesda, MD USA; 4 Biomedicine Division, School of Biosciences, Cardiff University, Cardiff, UK; 5 Centre for Ethics in Medicine, Population Health Sciences, Bristol Medical School, University of Bristol, Bristol, UK; 6 Bristol NIHR Biomedical Research Centre, Bristol, UK; 7 MRC Integrative Epidemiology Unit, University of Bristol, Oakfield House, Oakfield Grove, Bristol, UK

**Keywords:** Behavioural coding, Parent–infant interactions, Markov chain processes, ALSPAC, Grown in wales

## Abstract

Behavioural coding is time-intensive and laborious. Thin slice sampling provides an alternative approach, aiming to alleviate the coding burden. However, little is understood about whether different behaviours coded over thin slices are comparable to those same behaviours over entire interactions. To provide quantitative evidence for the value of thin slice sampling for a variety of behaviours. We used data from three populations of parent-infant interactions: mother-infant dyads from the Grown in Wales (GiW) cohort (*n* = 31), mother-infant dyads from the Avon Longitudinal Study of Parents and Children (ALSPAC) cohort (*n* = 14), and father-infant dyads from the ALSPAC cohort (*n* = 11). Mean infant ages were 13.8, 6.8, and 7.1 months, respectively. Interactions were coded using a comprehensive coding scheme comprised of 11–14 behavioural groups, with each group comprised of 3–13 mutually exclusive behaviours. We calculated frequencies of verbal and non-verbal behaviours, transition matrices (probability of transitioning between behaviours, e.g., from looking at the infant to looking at a distraction) and stationary distributions (long-term proportion of time spent within behavioural states) for 15 thin slices of full, 5-min interactions. Measures drawn from the full sessions were compared to those from 1-, 2-, 3- and 4-min slices. We identified many instances where thin slice sampling (i.e., < 5 min) was an appropriate coding method, although we observed significant variation across different behaviours. We thereby used this information to provide detailed guidance to researchers regarding how long to code for each behaviour depending on their objectives.

## Introduction

Microanalytic behavioural coding describes the categorisation of overt behaviours at high temporal resolution, capturing detailed information from observational data. This approach can be applied to a variety of interactions, and is effective for identifying subtle behaviours (Beebe et al., 2010). Parent-infant interaction studies have previously used microanalysis to improve understanding of behaviours across a range of contexts, e.g., associations between maternal behaviour and postpartum depression (Beebe et al., 2008) or infant behaviour and attachment outcomes (Koulomzin et al., 2002). Others have used behavioural coding to investigate many aspects of parent-infant interactions, including behaviour regulation (Feldman & Eidelman, 2007; Northrup & Iverson, 2020), synchrony and communication (Cote & Bornstein, 2021; Galligan et al., 2018; Papaligoura & Trevarthen, 2001) and impacts for infant cognitive and neuro-development (Feldman & Eidelman, 2003). Studies in this field have coded observations of varying lengths of time, including 2.5 min (Beebe et al., 2011), 5 min (Northrup & Iverson, 2020), and 15 min (Feldman & Eidelman, 2007).

Behavioural coding is a rigorous, iterative, and time-intensive process. Pesch and Lumeng (2017) outlined several factors that may increase the amount of time that researchers spend coding, including using coding schemes with complex behavioural categories, implementing novel coding schemes, and measuring contingencies between behaviours. Consequently, it is accepted practice to only code short portions of longer interactions (James et al., 2012), although these decisions are not always well justified or supported by evidence. James et al. (2012) examined researchers’ justifications for the length of the coded timeframe in 18 mother-infant interaction studies. The most common of these were “follows previous research” or “pilot study”, with only one study citing scientific evidence for their choice.

This evidence was a meta-analysis conducted by Ambady and Rosenthal (1992), which suggested that “thin slices”—defined as brief (< 5 min) observations of expressive behaviours—are predictive of a number of outcomes, such as deception, trustworthiness, and satisfaction. Many works have since investigated the use of thin slice sampling to predict outcome variables and have reproduced similar results. For example, Murphy (2007) demonstrated that 1-min slices were sufficient to predict participant intelligence, Hirschmann et al. (2018) found that 10- and 40-min slices were comparable for predicting maternal sensitivity, and Roter et al. (2011) showed that 1-min slices were adequate for predicting communication over sessions longer than 10 min. However, other studies have identified losses in predictive validity of various outcomes whilst using the thin slice sampling approach (e.g., Murphy et al., 2019; Wang et al., 2020).

Whilst there are many examples of studies investigating the suitability of thin slice sampling for outcome prediction, less is known about whether thin slice sampling of individual behaviours can accurately predict the same behaviour across the total interaction (or in the long-term). Few studies have compared behaviour proportions, frequencies and durations over thin slices and full session interactions, and those that have done so demonstrate conflicting findings. For example, Murphy (2005) found strong evidence for a moderate to high correlation between coding 1-, 2- and 3-min slices of non-verbal behaviours and full 15-min interactions, and Carcone et al. (2015) found comparable proportions of verbalisations in 1- and 2-min slices compared to the full 30-min session. Both studies found higher correlation as the lengths of slices increased. Findings from these studies indicate that behavioural features over thin slices may be predictive of those same behaviours over longer timeframes. Conversely, James et al. (2012) found evidence that continency measures of visual and vocal behaviours over 3- and 6-min slices differed significantly from those drawn from the full 18-min interactions. There is a clear need for further guidance or quantitative evidence to outline the value of varying lengths of coding, and how the accuracy of predictions may vary between different behaviours (e.g., verbal, visual).

Further, whilst thin slice sampling has currently been applied to mother-infant interactions in a small handful of studies (Hirschmann et al., 2018; James et al., 2012), the approach has not yet been applied to father-infant interactions, so to generate new insight in this area would be beneficial for future parent-infant interaction studies.

The aims of this work were two-fold: (1) to provide quantitative evidence to inform researchers decisions regarding the length of interaction needed for coding different behavioural groups, and (2) to supplement existing understanding of using thin slices in parent-infant interactions (particularly as analyses of this kind have not yet been applied to fathers). We hypothesised that measures drawn from parent and infant behaviours over longer slices (i.e. 3-, 4-min)—as opposed to shorter slices (i.e. 1-, 2-min)—would be most representative of measures drawn from fully coded, 5-min interactions. To address our aims, we compared behavioural measures over various slices of interactions using three distinct populations of parent-infant interactions, building upon previous works that have compared behavioural counts over thin slices and full sessions (e.g., Carcone et al., 2015; Murphy, 2005). Following each section of analysis (frequencies, transition matrices and stationary distributions), we have provided detailed guidance for interpreting how many minutes of coding may be necessary to capture the characteristic of each behaviour and measure.

## Methodology

### Participants

The datasets used in this research are comprised of videos of parent-infant interactions, taken from two cohort studies: Avon Longitudinal Study of Parents and Children (ALSPAC) and Grown in Wales (GiW). From these cohorts, we selected three distinct populations of participants: from GiW we used “Cardiff Mums” (*n* = 31 dyads), and from ALSPAC we used “Bristol Mums” (*n* = 14) and “Bristol Dads” (*n* = 11). For the three populations, we analysed 15 distinct, cumulative thin slices of lengths 1-, 2-, 3-, 4- and 5-min (described in more detail later). Further demographic information for each population is provided below.

*Cardiff Mums (n* = *31)*: Mothers and infants in the Cardiff population are participants from the Grown in Wales (GiW) cohort study, previously described in Janssen et al. (2018) and Savory et al (2020). Briefly, the GiW study aimed to examine the relationship between prenatal mood symptoms, placental genomic characteristics, and offspring outcomes. The GiW cohort is based in South Wales (UK), consisting of women recruited between 1 September 2015 and 31 November 2016 at a presurgical appointment prior to a booked elective caesarean section. Mothers were recruited if they met the criteria of a singleton term pregnancy without fetal anomalies and infectious diseases. At the time of recruitment, the cohort consisted of 355 women between the ages of 18 and 45.

One year after birth, all GiW participants were invited for an in-person assessment, where the video interactions were recorded. In total, 85 dyads attended the assessment between September 2016 and December 2017, and we selected 31 video interactions based on video quality for our work. We refer to these participants as “Cardiff Mums” and “Cardiff Infants”. The mean maternal age at the time of assessment was 35.4 years (*SD* = 0.33), and the average infant age was 13.8 months (*SD* = 0.98). 16 infants were male; 15 infants were female. In terms of parity, 16.1% were nulliparous and 83.9% were multiparous. For the mother's education, 16.1% were educated to GCSE level, 12.9% to A-Levels, 31% had an undergraduate degree, and 38.7% held a postgraduate degree.

*Bristol Mums (n* = *14)*: Mothers and infants in the Bristol Mums population are participants from the Avon Longitudinal Study of Parents and Children (ALSPAC). The study website (http://www.bristol.ac.uk/alspac/researchers/our-data/) contains details of all ALSPAC data that is available through a fully searchable data dictionary and variable search tool. ALSPAC study data were collected and managed using REDCap electronic data capture tools hosted at the University of Bristol (Harris et al., 2009). REDCap (Research Electronic Data Capture) is a secure, web-based software platform designed to support data capture for research studies.

Full cohort demographics and recruitment details have been provided previously elsewhere (Boyd et al., 2013; Fraser et al., 2013; Lawlor et al., 2019; Northstone et al., 2019). Briefly, ALSPAC is an ongoing longitudinal, population-based study based in Bristol, UK. The original cohort was recruited via a set of 14 541 pregnancies with expected delivery dates between 1 April 1991 and 31 December 1992. The children born to the original cohort (ALSPAC-G0) in 1992 are referred to as generation 1, or ALSPAC-G1, and the children born to those children over two decades later are referred to as generation 2, or ALSPAC-G2. Our work comprises mothers from ALSPAC-G1 (born in 1992), and their corresponding infants from ALSPAC-G2 (born over two decades later). We refer to these participants as “Bristol Mums” and “Bristol Infants (1)”.

Bristol Mums were recruited via a research clinic at the University of Bristol, which they attended approximately 6 months after the birth of their infant (mothers at the clinic were invited to take part in the headcam project). The videos used in this work were collected between July 2016 and July 2018. There were no selection criteria for mothers other than being part of the original ALSPAC cohort. Mean maternal age was 24.4 years (*SD* = 0.9), and mean age of infants was 29.5 weeks (*SD* = 1.4). Nine infants were male; five infants were female. In terms of parity, 78.6% were primiparous and 21.4% were multiparous. For the mother's education, 7.1% were educated to GCSE level, 71.4% to A-levels, and 7.1% held a higher education degree (14.3% unknown).

*Bristol Dads (n* = *11)*: Fathers and infants in the Bristol Dads population are also participants from ALSPAC-G1 and ALSPAC-G2, as described above. Explicitly, the fathers belong to generation 1, and were born to the original ALSPAC-G0 mothers in 1992, and the infants in these dyads were born over two decades later, comprising generation 2. We refer to these participants as “Bristol Dads” and “Bristol Infants (2)”. Bristol Dads were recruited via a father-specific research clinic (“Focus on Fathers”), which invited fathers to attend multiple assessments when their infant was 6 months old. Data was collected between July 2019 and 2020, and there were no specific selection criteria other than being a partner of an original ALSPAC participant. Mean paternal age was 27.5 (*SD* = 3.5), and mean age of infants was 30.8 weeks (*SD* = 3.8). Six infants were male; five infants were female. In terms of parity, 54.5% were primiparous and 45.5% were multiparous. For the father’s education, 63.6% were educated to GCSE level, and 18.2% to A-level (18.2% unknown).

### Video Recording Procedures

*Cardiff Mums (n* = *31)*: Interactions were recorded by a tripod-mounted video recorder. Mothers and infants were recorded during an unstructured, “free play” session in a research laboratory at Cardiff University. Free play means that mothers and infants were not provided with a set of play instructions, and were free to move about the room.

Two researchers were present at the time of video recording; one of whom was responsible for operating the video recorder (with the aim to keep as much as the infant and mother in shot as possible, but with a focus on the infant preferentially). The room set up was a soft play pen (that the infant was able to climb in and out of) placed in the middle of the room, and a number of toys placed inside it. There was also a bookcase displaying a number of toys at the back of the room. In the left hand corner of the room there was a chair for the mother to sit on. The mother was instructed to play as she normally would at home with the infant for 5 min, using any of the toys available in the room.

*Bristol Mums (n* = *15) and Bristol Dads (n* = *17)*: Videos for both populations were recorded by cameras worn on headbands by both the parent and the infant, capturing two separate videos for each interaction. First-person headcams have previously been shown to be reliable for capturing mother and infant behaviours (Lee et al., 2017). Separate headcam footage from both the parent and infant cameras were synced by researchers for coding purposes.

Bristol Mums and Dads were given the fully-charged wearable headcams, and asked to use them at home during different types of interactions. For the mothers, the interactions analysed in this study were classed as: “mealtime” (infant engages in eating; *n* = 11) and “stacking task” (mother and infant engage with stacking toy; *n* = 4). For the fathers, the interactions were classed as: “mealtime” (*n* = 10), “stacking task” (*n* = 3), “reading” (parent reads book to infant; *n* = 1), “bedtime” (parent puts infant to bed; *n* = 1), and “mixed” (combination of interaction types; *n* = 2). Due to the videos being taken in the home, it was possible for siblings/other caregivers/pets to be present during the interactions.

Our analyses feature 15 videos from the Bristol Mums population. These videos were provided by 14 individual mother-infant dyads, as one dyad provided two videos to the analyses. Explicitly, this dyad provided both a “feeding” and a “stacking task” video, whereas the other thirteen dyads each provided a single video representing one of the following activities: "feeding", "stacking task", "bedtime", "mealtime", or "reading". Our analyses also feature 17 videos from the Bristol Dads population, provided by 11 individual father-infant dyads. In this case, seven dyads provided one video, three dyads provided two videos, and one dyad provided four videos.

We ran the analyses twice—once using only one video from each dyad, and the second time using all the videos, including the second/third/fourth videos from some dyads. We looked to identify whether correlations were inflated due to re-using multiple videos from the same subjects, and also to see if the corresponding trends drastically differed in any way. For this, we carried out chi-squared analyses on the two separate datasets: one dataset comprised of the correlations in Tables 4 and 5 (including multiple videos per subject), and the equivalent data calculated using one video per subject only. The findings from this analysis are presented in the “Appendix” (“Analysis of Multiple Videos per Subject” section); However, it is clear from these results that the two datasets were considerably similar, and there was no evidence to indicate that including multiple videos inflated our correlations. So, in the interest of including all available observations and maximising statistical power to the analyses, we made the decision to include the multiple videos per subject where these existed.

### Behavioural Coding

All interactions were coded on an event-basis using Noldus Observer XT 14.0 (Noldus, 2021). The full micro-coding manual used for this research is available online (Costantini et al., 2021). The behavioural coding scheme, including overarching codes and individual subcodes, is summarised in Table 1. All populations used the same coding scheme, although the Cardiff population applied a reduced subset of behavioural categories (see Table 1).

**Table 1:** Behaviours coded for each of the three parent-infant populations, categorised by behavioural group

Behavioural group	Behaviour	Behavioural group	Behaviour	Behavioural group	Behaviour
Caregiver posture	Lying down Lie on side Sit on floor Sit on object Stand up Crawl Crouched down Jump Walk Run Dance NPTC^a^ caregiver posture/action	Infant posture	Lie down Lie on one side Sit on the floor Sit on an object Standing Crawling Jumping Walking Running Held/in hold Try to move in another way NPTC^a^ infant posture/action	Visual attention	Look at infant Look at caregiver 1 Look at caregiver 2 Look at same object/joint attention Look at focus object Look at different object Look at other object Look at object outside of view Look at sibling Look at other person Look at distraction No visual attention NPTC^a^ visual attention
Caregiver vocalisation	Speech Musical sounds Laugh Nervous laugh Vocal imitation Bodily sounds Scream Vocal tics Non-verbal sound Silent NPTC^a^ caregiver vocalisation	Infant vocalisation	Laughing Distressed Non-distress Imitating sounds Babbling First words Screaming Bodily Sounds Silent/none of the above NPTC^a^ infant vocalisation	**Head orientation**^**b**^	**Vis-à-vis—infant and caregiver** **Slight (30°–90°) aversion right** **Slight (30°–90°) aversion left** **Full (90°) aversion right** **Full (90°) aversion left** **Arch aversion** **Head not in view of infant** **NPTC**^**a**^** head orientation**
Caregiver body orientation	Body oriented to infant Body oriented to other caregiver Body oriented to sibling Body oriented to other person/object Body oriented to object (focus of the activity) NPTC^a^ caregiver body orientation	Infant body orientation	Body oriented to caregiver 1 Body oriented to caregiver 2 Body oriented to sibling Body oriented to different person/object Body oriented to object (focus of activity) NPTC^a^ code infant body orientation	**Facial expression**^**b**^	**Neutral/Alert** **Positive** **Smile** **Negative** **Disgust** **Surprise** **Woe face** **Mock surprise** **Face not visible** **None of the above**
Touch right hand (R)	Infant touch R Caregiver touch R No infant touch R No caregiver touch R NPTC^a^ touch R	Touch left hand (L)	Infant touch L Caregiver touch L No infant touch L No caregiver touch L NPTC^a^ touch L	**Hand Movements**^**b**^	**Pointing** **Reaching** **Clapping** **Waving** **Gesticulating** **Stacking attempt** **Banging** **Other hand movements** **No hand movements** **NPTC**^**a**^** hand movements**
Caregiver proximity	Out of reach Within reach Loom NPTC^a^ caregiver proximity	Physical play	Physical play evident No physical play NPTC^a^ physical play		

“Behavioural group” refers to an overarching behavioural category, comprised of a subset of mutually exclusive, exhaustive “behaviours”. The full documentation for this coding scheme is available online (Costantini et al., 2021)

^a^NPTC, not possible to code. This code indicates that no behaviour within the behavioural group could be coded. This could be because the subjects eyes are not in view of the headcam, for example, leading to a code of “NPTC Visual Attention”

^b^Code was not applied to Cardiff Mothers population (also indicated by bold text)

Within each behavioural group (e.g., Caregiver Posture), behaviours (e.g., Lying down, Lie on side) are mutually exclusive and exhaustive; this means that at each point in time, exactly one behaviour from each behavioural group must be coded. It follows that at any given moment, Cardiff Mums must have 8 codes applied, Cardiff Infants must have 7, Bristol Mums and Dads must have 11, and Bristol Infants (1) and (2) must have 10.

The original Bristol videos varied in length (ranging from 5 to 20 min). This is because the parents were advised to record a “typical” interaction, so the length of the video was therefore dependent on how long the infant naturally took to be fed, how long the parent took to put the infant to bed, etc. A 5 min portion of each interaction was chosen for coding, a choice that was made for a previous, unrelated study involving the Bristol videos. This choice is consistent with previous studies in thin slice sampling that have also used 5-min slices to represent entire sessions (e.g., Murphy, 2007; Murphy et al., 2019). At the beginning of the Bristol Mums and Bristol Dads videos, the parent would typically spend a few moments setting up the infant headcam and reading the study information sheet provided, before starting the interaction. In line with guidance from an information sheet, the parent would announce the “official” start of the interaction by stating “Today it is X date, and we are doing X activity”. It is from this vocalisation that the 5-min coded segment began. For the Cardiff videos, the recording began immediately once the participant ID had been shown to the camera. Each interaction lasted for 5 min, and the entire interaction was coded.

The three populations were coded by separate researchers, all of whom had been independently trained in using the coding scheme. Cardiff Mums were coded by one researcher (HT), Bristol Mums were coded by three researchers (IC, AC, RP), and Bristol Dads were coded by four researchers (MK, PC, JS, LM). For each of the three populations, 20% of videos were double coded for reliability purposes: 6 from the Cardiff Mums population, 3 from the Bristol Mums and 4 from the Bristol Dads. Two additional researchers were recruited for double coding (HD, RB). Inter-rater reliability was assessed using Cohen’s kappa, separated according to behavioural group. All reliability analyses were conducted using the Observer XT 14.0 (Noldus, 2021). These analyses are provided in Table 2.

**Table 2:** Reliability analyses separated by population and behavioural group

Behavioural group	Population		
	Cardiff mums (n = 6)	Bristol mums (n = 3)	Bristol dads (n = 4)
Proximity	0.96 (0.92–0.99)^a^	0.93 (0.90–0.99)^a^	0.96 (0.88–0.99)^a^
Body orientation	0.97 (0.93–0.99)	0.79 (0.69–0.96)	0.97 (0.91–0.99)
Head orientation	–	0.93 (0.88–0.98)	0.78 (0.68–0.88)
Vocalisation	0.94 (0.93–0.96)	0.79 (0.76–0.80)	0.91 (0.90–0.93)
Posture	0.92 (0.80–0.97)	0.88 (0.69–0.98)	0.97 (0.90–0.99)
Facial expressions	–	0.67 (0.62–0.76)	0.85 (0.66–0.99)
Touch left hand	0.78 (0.73–0.91)	0.89 (0.77–0.98)	0.88 (0.82–0.99)
Touch right hand	0.79 (0.66–0.85)	0.85 (0.68–0.97)	0.91 (0.82–0.97)
Physical play	0.84 (0.77–0.94)	0.73 (0.64–0.85)	0.88 (0.77–0.98)
Hand movements	–	0.90 (0.83–0.99)	0.94 (0.77–0.98)
Visual attention	0.84 (0.69–0.99)	0.75 (0.60–0.85)	0.84 (0.78–0.97)

Inter-rater reliability was calculated using Cohen’s kappa. Minimum and maximum kappa for a behavioural group appear in parentheses

^a^Code was only applied to parents within the population, so kappa calculation does not include infant behavioural coding

### Data Analysis

Each parent-infant interaction was split into 15 distinct thin slices, representing various cumulative minute combinations of the full, 5-min session. For clarity, we named these slices according to the minutes of the interaction that they include. For example, slice *One* represents the coded data from the first minute of the interaction, slice *OneTwo* represents the coded data from the first two minutes of the interaction, etc. This gives five 1-min slices (*One*, *Two*, *Three*, *Four*, *Five*), four 2-min slices (*OneTwo*, *TwoThree*, *ThreeFour*, *FourFive*), three 3-min slices (*OneTwo-Three*, *TwoThreeFour*, *ThreeFourFive*), two 4-min slices (*OneTwoThreeFour*, *TwoThreeFourFive*) and the full 5-min slice (*OneTwoThreeFourFive*) (i.e. the fully coded session).

Our work applies Markov Chain analysis—specifically, transition matrices and stationary distributions—to the parent-infant interactions. We outline these methodologies below, and how we applied them to our work. However, a more thorough walkthrough with a representative example can be found in “Markov Chain Analyses Walkthrough with Example” section of the “Appendix”. The walkthrough details the mathematics involved in deriving transition matrices and stationary distributions, and also how to interpret them.

In brief, Markov chains are probabilistic models describing processes of events over time. The Markov chains in this work are discrete-time—as behaviours are analysed on a second-by-second basis—and finite state—as there are a finite number of behavioural states. An important feature of Markov chains is that in order to predict the state (or behaviour) at time *n*, we only need to know the state (behaviour) at time *n* − 1.

**Fig. 1:**
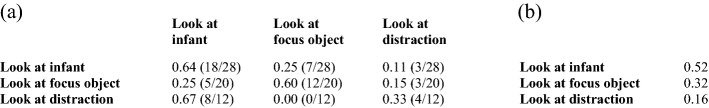
Example transition matrix (**a**) for a 60 s period, and the corresponding stationary distribution (**b**)

Transition matrices contain the probabilities of transitioning between states (Gagniuc, 2017), in our case, transitioning between behaviours within the same behavioural group. An example transition matrix is given in Fig. 1a, showing the probabilities of a mother transitioning between Visual Attention behaviours over a 60 s period. In our work, transitions were calculated on a second-by-second basis, meaning that 60 transitions between behaviours were recorded for a 60 s period. In order to derive the transition matrices, we recorded the behaviours that occurred at second 0, second 1, second 2, etc., then calculated the number of transitions between all behavioural states. The example matrix in Fig. 1a shows that 28 s were spent looking at the infant, 20 s were spent looking at the focus object, and 12 s were spent looking at a distraction. Additionally, the example shows that the probability of transitioning from “Look at infant” to “Look at infant” is 0.64, to “Look at focus object” is 0.25, and to “Look at distraction” is 0.11. It is important to note that rows must sum to 1.

A stationary distribution is a vector representing the long-term probabilities of being within behavioural states (Gagniuc, 2017). The full derivation of a stationary distribution is detailed in “Markov Chain Analyses Walkthrough with Example” section of the “Appendix”, but in brief, it is derived from the equation *s* = *sT*, where *T* is the *n* × *n* transition matrix and *s* is the 1 × *n* stationary distribution row vector (Ross, 2014). An example stationary distribution is given in Fig. 1b, derived from the transition matrix in Fig. 1a. This may be interpreted as the long-run proportion of time that the mother spends within each visual attention state. Here, over 100 s, we would expect the mother to be in the state “Look at infant” for 52 s, “Look at focus object” for 32 s, and “Look at distraction” for 16 s. In this way, we can use the values within stationary distributions as measures for duration of behaviours (for this reason, we have not included specific behaviour duration measures within our analyses, in order to avoid repetition).

As the length of thin slice increases, the transition matrices become populated with more data, and the stationary distributions become more precise. Therefore, we expect that transition matrices and stationary distributions generated from the longest thin slices will be most similar to those generated by the full interactions.

The earliest application of Markov processes to the mother-infant dyad was performed by Freedle and Lewis (1971), who illustrated how these methods could be used to identify sequences of infant vocalisation behaviours. This work outlined the value of Markov modelling for interaction studies; specifically, how current behavioural states within transition matrices influence the conditional probability of the subsequent behavioural states, and how the diagonal probabilities within the matrices can be used to estimate the likelihood of the subject remaining within a given state (a large probability on the diagonal indicates persistence of one behavioural state over time). Further applications of Markov Chain analysis to mother-infant dyads include: investigating differences in vocal affect in depressed and non-depressed mothers (Friedman et al., 2010), differentiating secure vs. avoidant attachment (Koulomzin et al., 2002), quantifying vocal reciprocity (Anderson et al., 1977), and understanding the process of soothing distressed infants (Stifter & Rovine, 2015). To our knowledge, no existing studies have compared transition matrices and stationary distributions over thin slices and full session interactions, in any context.

These three measures of behaviour—frequencies, transition matrices and stationary distributions—were calculated at each of the 15 slices, for both subjects within each population. We used Pearson correlations to measure the strength of the relationships between behavioural frequencies over thin slices and fully coded interactions. We also calculated the absolute differences between: (1) the rows within the transition matrices, and (2) the full stationary distribution vectors. These were calculated over each thin slice and full 5-min session. Finally, chi-squared analyses were employed to evaluate whether these measures, calculated over 1-, 2-, 3- and 4-min slices, were comparable to the full session counterparts. All calculations were conducted using Python 3.8.

Following each of the three analyses, we chose some example “level of agreement” between the thin slice and full session measures, which would indicate that the behavioural measure over the thin slice was adequately representative of that same behavioural measure over the full session (e.g., a “very strong” Pearson correlation, or *p* < 0.05 for a chi-squared test between two transition matrices). We used this information to provide guidance at the end of each section, outlining how to interpret an appropriate thin slice for each specific behaviour.

## Results

### Frequencies of Parent and Infant Behaviours

A total of 19 309 behaviours were coded in the Cardiff Mums population, 7 296 in Bristol Mums, and 8 964 in Bristol Dads; parent behaviours accounted for 57.3%, 53.9% and 54.9% of data, respectively. The mean frequencies of all behaviours over each thin slice are provided within Tables 7, 8, 9, 10, 11 and 12 of the “Appendix” (a separate table is given for each population).

These data show that frequencies of behaviours were almost always higher in the Cardiff population than in both Bristol Mums and Bristol Dads. To illustrate this, note that for the 1-min slice *One*, the mean frequency for Cardiff Mum Proximity (2.90) was higher than the equivalent values for both Bristol Mums (1.87) and Bristol Dads (2.24). Similarly, the mean frequency for Cardiff Infants Visual Attention for the 2-min slice *OneTwo* (26.16) was higher than the equivalent values for both Bristol Infants (1) and (2) (15.33 and 16.24, respectively). These differences could be attributed to the difference in setting between the Cardiff and Bristol populations (i.e., in-lab vs. at home).

We can also compare frequencies of parent and infant behaviours. For example, across all populations and slices, parent vocalisation occurred more frequently than infant vocalisation (unsurprising, as the infants in this work were not of speaking age). Other behaviours—such as Touch and Physical Play—also often occurred more frequently in parents than infants. Conversely, Head Orientation and Body Orientation were both more frequent in infants than in parents for all three populations. Visual Attention, Facial Expressions and Hand Movements occurred with similar frequencies throughout all parents and infants.

A key finding from these analyses is that many behaviours were most frequent in the earliest slices of the interaction, and least frequent in the latest slices. These patterns together were most strongly prevalent in the parents’ behaviours, much more so than the infants (although similar patterns did emerge to a lesser degree in Bristol Infants (1) and (2)). There are many examples to illustrate how slice *Five* shows lower frequencies than other 1-min slices: Cardiff mums Touch R (*One* 14.68, *Two* 14.77, *Three* 13.71, *Four* 14.65 and *Five* 9.94), Bristol Mums Vocalisation (*One* 17.80, *Two* 18.00, *Three* 19.60, *Four* 15.87, *Five* 12.73), and Bristol Dads Head Orientation (*One* 7.59, *Two* 6.29, *Three* 5.53, *Four* 5.06 and *Five* 4.65). This pattern also extends into the other, longer slices that occur latest in the interaction, for example: Cardiff mums Visual Attention (*OneTwo 31.90, TwoThree* 29.48, *ThreeFour* 26.58 and *FourFive* 24.68), Bristol Mums Facial Expressions (*OneTwoThree* 27.27, *TwoThreeFour* 28.40 and *ThreeFourFive* 26.67) and Bristol Dads Posture (*OneTwoThreeFour* 5.76, *TwoThreeFourFive* 4.88).

Conversely, many behaviours were most frequent in the earliest slices, for example: Bristol Dads Body Orientation (*One* 2.82, *Two* 2.65, *Three* 1.53, *Four* 1.47 and *Five* 1.29), Bristol Mums Touch L (*OneTwo* 8.93, *TwoThree* 8.07, *ThreeFour* 8.27, *FourFive* 7.07), and Cardiff Mums Proximity (*OneTwoThree* 7.45, *TwoThreeFour* 7.06, *ThreeFourFive* 7.16). This pattern is strongest in the Cardiff Mums population: slice *One* has the highest frequency of behaviours compared to all other 1-min slices for all behaviours except Touch R, and slices *OneTwo*, *OneTwoThree*, and *OneTwoThreeFour* have the highest frequency of all behaviours compared to other 2-, 3- and 4-min slices, respectively. These findings should be interpreted with caution in many cases, however, as it is often true that the standard deviations are large and overlap across slices. We provide our interpretations of these findings within the discussion.

*Pearson correlations*: Using the frequency data, we calculated Pearson correlations to evaluate the relationship between the frequency of behaviours extracted from the 1-, 2-, 3- and 4-min slices and the fully coded interactions (shown in Tables 3, 4, 5). Schober et al. (2018) suggest that correlations from 0.10 to 0.39 may be interpreted as “weak”, those from 0.40 to 0.69 are “moderate”, those from 0.70 to 0.89 are “strong”, and those higher than 0.90 may be classed as “very strong”.

Most correlations were found to be either “strong” or “very strong”, with three examples demonstrating “very strong” correlations between all thin slices and full sessions (i.e., Bristol Mums Body Orientation, Bristol Dads Hand Movements and Bristol Infants (2) Physical Play). There were several instances of “weak” correlation between a 1 or 2-min slice and full session (e.g., Cardiff Mums Physical Play, slice *Five*; Cardiff Mums Posture, slice *Four*; Bristol Dads Posture, slices *Three*, *Four* and *ThreeFour*), which may in part be explained by the frequencies of these behaviour being very low (see Tables 7 and 11 of the “Appendix”). There were also many examples of “moderate” correlations between 1-, 2- and 3-min slices and full sessions (e.g., Cardiff Infants Touch R, slice *One*; Bristol Mums Visual Attention, slices *One*, *Two* and *OneTwo*; Cardiff Mums Posture, slice *ThreeFourFive*).

Within Tables 3, 4 and 5 below, we have also selected an example “criterion” to indicate a strong correlation between frequencies of a given thin slice and the full interaction; specifically, where r > 0.9, as shown in bold. We have chosen to highlight correlations of this magnitude as the goal of this analysis is to understand which slice pairings yield greatest agreement, and a strength of association of this magnitude indicates that slices are closely related. However, it should be noted that this criterion is provided as an example baseline for our own interpretations, and we recognise that for the benefit of other research aims, it may be more appropriate for researchers to select their own criterion accordingly.

**Table 3:** Cardiff mums: correlations between frequencies over thin slices and full interactions

	1-min slices					2-min slices				3-min slices			4-min slices	
	One	Two	Three	Four	Five	One Two	Two Three	Three Four	Four Five	One Two Three	Two Three Four	Three Four Five	One Two Three Four	Two Three Four Five
*Mother behaviour*														
Proximity	0.69	0.68	0.69	0.64	0.36	0.79	0.86	0.74	0.77	**0.92**	0.85	0.84	**0.94**	**0.93**
Body^a^	0.66	0.75	0.50	0.60	0.41	0.83	0.83	0.67	0.71	**0.91**	0.85	0.80	**0.94**	**0.91**
Visual^b^	0.72	**0.90**	**0.90**	**0.90**	0.72	**0.91**	**0.95**	**0.94**	0.88	**0.96**	**0.97**	**0.94**	**0.98**	**0.97**
Posture	0.72	0.60	0.55	0.37	0.50	0.76	0.73	0.54	0.56	0.87	0.73	0.66	**0.94**	**0.82**
Touch R	0.81	0.75	0.89	0.81	0.69	0.88	**0.93**	**0.93**	0.89	**0.97**	**0.97**	**0.92**	**0.98**	**0.98**
Touch L	0.78	0.79	0.88	0.79	0.65	0.85	**0.93**	0.89	0.80	**0.94**	**0.96**	**0.90**	**0.98**	**0.97**
Physical play	0.61	0.67	0.61	0.46	0.18	0.80	0.74	0.72	0.47	0.89	0.82	0.72	**0.96**	**0.87**
Vocalisation	0.86	0.80	0.80	0.83	0.82	**0.90**	**0.92**	**0.92**	**0.90**	**0.95**	**0.97**	**0.95**	**0.98**	**0.99**
*Infant behaviour*														
Body^a^	0.57	0.61	0.74	0.72	0.81	0.75	0.82	0.85	**0.93**	**0.92**	0.89	**0.94**	**0.96**	**0.97**
Visual^b^	0.73	0.86	0.84	0.77	0.73	0.86	**0.93**	0.86	0.85	**0.94**	**0.95**	**0.91**	**0.98**	**0.98**
Posture	0.83	0.77	0.86	0.72	0.70	0.89	0.89	**0.90**	0.85	**0.94**	**0.94**	**0.95**	**0.97**	**0.98**
Touch R	0.62	0.76	0.85	0.82	0.73	0.85	**0.91**	0.88	**0.90**	**0.95**	**0.94**	**0.95**	**0.98**	**0.97**
Touch L	0.65	0.82	0.82	0.85	0.78	0.83	**0.92**	**0.90**	0.89	**0.94**	**0.95**	**0.93**	**0.98**	**0.98**
Physical play	0.45	0.83	0.66	0.62	0.48	0.84	0.84	0.79	0.66	**0.90**	**0.90**	0.85	**0.96**	**0.95**
Vocalisation	0.76	0.83	0.79	0.79	0.55	**0.91**	**0.90**	**0.91**	0.78	**0.94**	**0.95**	**0.91**	**0.98**	**0.97**

Values corresponding to values of r > 0.90 have been made bold

^a^Body orientation, ^b^Head orientation, ^c^Visual attention, ^d^Facial expressions, ^e^Hand movements

**Table 4:** Bristol mums: correlations between frequencies over thin slices and full interactions

	1-min slices					2-min slices				3-min slices			4-min slices	
	One	Two	Three	Four	Five	One Two	Two Three	Three Four	Four Five	One Two Three	Two Three Four	Three Four Five	One Two Three Four	Two Three Four Five
*Mother behaviour*														
Proximity	0.68	**0.96**	**0.94**	0.77	0.88	**0.90**	**0.96**	**0.93**	**0.95**	**0.98**	**0.95**	**0.97**	**0.99**	**0.97**
Body^a^	**0.92**	**0.91**	**0.94**	**0.93**	**0.90**	**0.98**	**0.93**	**0.97**	**0.99**	**0.99**	**0.98**	**0.99**	**0.99**	**0.99**
Head^b^	0.84	**0.92**	**0.97**	0.80	0.84	**0.96**	**0.97**	**0.98**	**0.91**	**0.98**	**0.98**	**0.98**	**0.99**	**0.99**
Visual^c^	0.43	0.68	**0.92**	**0.90**	0.89	0.58	**0.94**	**0.95**	**0.92**	**0.90**	**0.99**	**0.95**	**0.97**	**0.99**
Face^d^	0.88	0.88	**0.98**	**0.94**	0.83	0.89	**0.95**	**0.99**	**0.93**	**0.94**	**0.99**	**0.99**	**0.98**	**0.99**
Posture	0.74	0.76	0.86	0.77	0.86	**0.91**	0.85	0.86	0.86	**0.96**	0.83	0.88	**0.99**	0.86
Touch R	0.88	**0.91**	0.86	**0.96**	**0.92**	**0.91**	**0.97**	**0.96**	**0.96**	**0.98**	**0.99**	**0.96**	**0.99**	**0.99**
Touch L	**0.90**	0.89	**0.94**	0.89	0.81	**0.98**	**0.96**	**0.97**	**0.99**	**0.99**	**0.99**	**0.99**	**0.99**	**0.99**
Hands^e^	0.85	**0.99**	**0.97**	**0.97**	0.74	**0.94**	**0.98**	**0.97**	**0.99**	**0.99**	**0.98**	**0.99**	**0.99**	**0.99**
Physical play	**0.90**	**0.91**	**0.98**	0.89	0.92	**0.90**	**0.99**	**0.97**	**0.92**	**0.98**	**0.99**	**0.95**	**0.99**	**0.99**
Vocalisation	0.87	**0.92**	**0.93**	0.84	0.89	**0.92**	**0.99**	**0.93**	**0.94**	**0.97**	**0.98**	**0.96**	**0.99**	**0.99**
*Infant behaviour*														
Body^a^	0.85	0.78	**0.94**	**0.96**	**0.97**	0.86	**0.95**	**0.97**	**0.97**	**0.97**	**0.99**	**0.98**	**0.99**	**0.99**
Head^b^	**0.92**	0.84	**0.94**	0.89	**0.90**	**0.92**	**0.97**	**0.97**	**0.93**	**0.97**	**0.99**	**0.96**	**0.99**	**0.99**
Visual^c^	0.77	0.79	**0.94**	**0.93**	0.87	0.85	**0.92**	**0.96**	**0.95**	**0.94**	**0.97**	**0.97**	**0.98**	**0.99**
Face^d^	0.82	0.61	**0.94**	**0.92**	0.69	0.75	0.87	**0.96**	0.85	0.89	**0.94**	**0.93**	**0.94**	**0.98**
Posture	0.80	0.93	0.83	**0.92**	0.85	**0.93**	**0.96**	**0.96**	**0.98**	**0.98**	**0.97**	**0.98**	**0.99**	**0.99**
Touch R	0.69	0.83	0.86	0.87	0.71	0.81	**0.94**	**0.93**	0.83	**0.92**	**0.97**	**0.93**	**0.97**	**0.97**
Touch L	0.85	**0.93**	**0.95**	**0.93**	**0.94**	**0.92**	**0.97**	**0.97**	**0.97**	**0.97**	**0.98**	**0.98**	**0.99**	**0.98**
Hands^e^	0.69	0.82	**0.95**	**0.91**	**0.93**	0.83	**0.96**	**0.95**	**0.92**	**0.95**	**0.97**	**0.95**	**0.99**	**0.97**
Physical play	0.71	**0.92**	**0.92**	**0.96**	0.85	0.84	**0.99**	**0.95**	**0.94**	**0.95**	**0.99**	**0.94**	**0.99**	**0.98**
Vocalisation	0.87	**0.97**	**0.96**	**0.94**	**0.92**	**0.97**	**0.98**	**0.98**	**0.98**	**0.99**	**0.99**	**0.99**	**0.99**	**0.99**

Values corresponding to values of r > 0.90 have been made bold

^a^Body orientation, ^b^Head orientation, ^c^Visual attention, ^d^Facial expressions, ^e^Hand movements

**Table 5:** Bristol dads: correlations between frequencies over thin slices and full interactions

	1-min slices					2-min slices				3-min slices			4-min slices	
	One	Two	Three	Four	Five	One Two	Two Three	Three Four	Four Five	One Two Three	Two Three Four	Three Four Five	One Two Three Four	Two Three Four Five
*Father behaviour*														
Proximity	0.72	**0.93**	0.68	0.83	0.82	**0.94**	**0.96**	0.84	0.88	**0.97**	**0.97**	**0.95**	**0.99**	**0.98**
Body^a^	0.86	**0.95**	0.67	0.85	0.48	**0.99**	**0.96**	0.83	**0.94**	**0.99**	**0.95**	**0.95**	**0.99**	**0.97**
Head^b^	0.88	0.83	0.84	0.88	0.85	**0.94**	**0.95**	**0.91**	**0.98**	**0.99**	**0.97**	**0.96**	**0.99**	**0.99**
Visual^c^	0.84	**0.90**	**0.93**	**0.94**	0.85	**0.96**	**0.95**	**0.97**	**0.96**	**0.99**	**0.97**	**0.98**	**0.99**	**0.99**
Face^d^	0.81	**0.91**	**0.94**	**0.92**	**0.91**	**0.94**	**0.96**	**0.98**	**0.95**	**0.97**	**0.99**	**0.98**	**0.99**	**0.99**
Posture	0.74	0.57	0.38	0.00	0.60	**0.94**	0.70	0.38	0.60	**0.97**	0.70	0.78	**0.97**	0.82
Touch R	**0.96**	**0.90**	0.88	0.86	0.82	**0.97**	**0.96**	**0.91**	**0.93**	**0.98**	**0.97**	**0.97**	**0.99**	**0.99**
Touch L	0.86	**0.91**	**0.95**	0.82	0.88	**0.97**	**0.96**	**0.97**	**0.96**	**0.99**	**0.98**	**0.99**	**0.99**	**0.99**
Hands^e^	**0.97**	**0.91**	**0.95**	**0.93**	**0.92**	**0.98**	**0.96**	**0.97**	**0.97**	**0.99**	**0.99**	**0.99**	**0.99**	**0.99**
Physical play	**0.98**	0.86	**0.91**	**0.90**	0.86	**0.98**	**0.96**	**0.94**	**0.95**	**0.98**	**0.98**	**0.99**	**0.99**	**0.99**
Vocalisation	0.76	0.87	**0.90**	0.75	0.80	0.88	**0.96**	0.89	0.88	**0.96**	**0.97**	**0.94**	**0.98**	**0.99**
*Infant behaviour*														
Body^a^	0.68	0.79	0.71	0.82	0.67	0.89	0.79	0.82	0.82	**0.96**	0.84	**0.90**	**0.98**	**0.91**
Head^b^	0.80	0.89	0.86	0.88	**0.91**	**0.94**	**0.93**	**0.94**	**0.94**	**0.98**	**0.96**	**0.97**	**0.99**	**0.98**
Visual^c^	**0.92**	**0.97**	**0.94**	0.89	**0.94**	**0.97**	**0.97**	**0.98**	**0.96**	**0.99**	**0.99**	**0.99**	**0.99**	**0.99**
Face^d^	**0.95**	**0.95**	**0.94**	**0.96**	0.84	**0.97**	**0.97**	**0.97**	**0.97**	**0.99**	**0.98**	**0.99**	**0.99**	**0.99**
Posture	0.72	0.58	0.78	0.78	0.68	0.88	0.81	0.78	0.84	**0.97**	0.86	0.84	**0.99**	0.89
Touch R	0.85	**0.98**	**0.97**	**0.96**	**0.97**	**0.98**	**0.98**	**0.98**	**0.98**	**0.99**	**0.99**	**0.99**	**0.99**	**0.99**
Touch L	0.71	**0.97**	**0.95**	0.89	0.87	**0.93**	**0.99**	**0.96**	**0.96**	**0.97**	**0.98**	**0.98**	**0.99**	**0.99**
Hands^e^	0.83	**0.90**	0.89	**0.96**	0.89	**0.95**	**0.94**	**0.98**	**0.96**	**0.97**	**0.98**	**0.98**	**0.99**	**0.99**
Physical play	**0.97**	**0.98**	**0.97**	**0.98**	**0.97**	**0.99**	**0.98**	**0.99**	**0.99**	**0.99**	**0.99**	**0.99**	**0.99**	**0.99**
Vocalisation	0.66	**0.94**	0.67	**0.96**	**0.91**	**0.93**	**0.93**	**0.96**	**0.96**	**0.94**	**0.99**	**0.98**	**0.99**	**0.99**

Values corresponding to values of r > 0.90 have been made bold

^a^Body orientation, ^b^Head orientation, ^c^Visual attention, ^d^Facial expressions, ^e^Hand movements

These data lend support to our hypothesis that longer slices would demonstrate the highest correlation with fully coded sessions. The lowest correlations were generally found between the full sessions and slices of length 1-min (mean = 0.81, range = 0.00–0.99), followed by those of length 2-min (mean = 0.90, range = 0.38–0.99), 3-min (mean = 0.94, range = 0.66–0.99) and 4-min (mean = 0.97, range = 0.82–0.99). Specifically, as the slice length increased, behavioural frequencies showed stronger correlations with the fully coded interactions. This was shown to be true across all behaviours, populations, and subjects. As an example, for Cardiff Infant Body Orientation, the correlations for 1-min slices (r(31) = [0.57, 0.61, 0.74, 0.72, 0.81]) were lower than for 2 min slices (r(31) = [0.75, 0.82, 0.85, 0.93]); these were lower than the 3-min slice correlations (r(31) = [0.92, 0.89, 0.94]); and these were lower than the 4-min slice correlations (r(31) = [0.96, 0.97]).

Correlations varied in strength depending on behavioural group. For example, across all slice lengths and populations, Caregiver Touch R correlations were greater than 0.69, Caregiver Vocalisation correlations were greater than 0.75, and Caregiver Head Orientation correlations were greater than 0.80. However, Posture, for example, showed generally weaker correlations both over shorter slices (e.g., Bristol Dads slice *Three*, r = 0.38), and longer slices (e.g., Cardiff Mums slice *ThreeFourFive*, r = 0.66).

Additionally, by providing correlations for individual slices, we can begin to see which portion of the interaction the behaviours best correlate with the full sessions. For the Cardiff Mums population, the original interactions and recordings were all of duration 5 min, so we can fully interpret which portion of the session—the beginning, middle or end—was most (and least) representative of the full session. We cannot speculate in this way for the Bristol Mums and Dads populations, given that many original videos used in were originally longer than 5 min, and these interactions were cut short so to not include the original “ending” of the interaction. For these two populations, we can however look to compare how the slices at the beginning of the interaction compare to those that occur later.

Looking first at 1-min slices, our findings reveal that the middlemost slices best correlated with the full session. This is evident from the bold text in Tables 3, 4 and 5, as most bold values are within columns *Two*, *Three* and *Four* for 1-min slices (particularly for the Bristol Mums and Dads populations). A good illustration of this pattern is Bristol Dads Visual Attention (r(17) = [0.84, 0.90, 0.93, 0.94, 0.85]). Here, slices *Two*, *Three* and *Four* have very strong correlations, whereas slices *One* and *Five* have only strong correlations. Another example is Cardiff Infants Touch R (r(31) = [0.62, 0.76, 0.85, 0.82, 0.73]. In this instance, the highest correlations are in slices *Three* and *Four*, followed by slice *Two*, then slice *Five*, then slice *One*.

For 2-min slices, it is less clear whether any specific slice correlated better than another. In some instances, slice *OneTwo* had the highest correlation (e.g., Bristol Dads Touch R), in others, slice *TwoThree* (e.g., Bristol Mums Vocalisation), slice *ThreeFour* (e.g., Bristol Infants (2) Hand Movements), or *FourFive* (e.g., Cardiff Infants Body Orientation). We observed variation by both behaviour and population. For 3-min slices, we observed that highest correlations most commonly arose for the earliest slice, *OneTwoThree*, whilst the latest slice, *ThreeFourFive*, contained the lowest correlations. These trends were particularly evident within Cardiff Mums, Cardiff Infants and Bristol Mums. A similar result was found for 4-min slices, where slice *OneTwoThreeFour* generally outperformed slice *TwoThreeFourFive* (although values are very similar, *TwoThreeFourFive* was the only 4-min slice to contain r < 0.9).

It is worth mentioning that these values may be inflated, given that the thin slices are themselves contained within the full session (and therefore represent a higher portion of the full session as the slice lengths increase). This could be addressed by evaluating correlations between non-overlapping slices. We have provided a brief example of such an analysis within the “Appendix” (see “Additional Non-overlapping Slices Analyses” section). However, there are a very large number of potential thin slice/full session combinations, and so on account of paper length constraints we have not provided a comprehensive analysis here. Future work could therefore aim to address this limitation, by evaluating patterns between various combinations of non-overlapping slices.

*Selecting Thin Slices Based on Frequencies*: Here we exemplify how researchers could use our analysis to determine how long to code for a given behaviour. In brief, researchers would choose a behaviour of interest, use Tables 3, 4 and 5 to evaluate correlations between thin slices and full-sessions for that behaviour, and use the example criterion provided to determine the appropriate thin slice length to code.

The bold values in Tables 3, 4 and 5 above provide an example “criterion” for a sufficient correlation between frequencies over thin slices and full interactions, i.e., r > 0.9 for all slices of the specified length. This criterion suggests that it is only necessary to code a specific behaviour up until the first fully-bold region within the corresponding table row, where all slices of a given length are suitably correlated with the full-session. For example, data for Cardiff Mums in Table 3 suggests the following coding durations: 2 min for Vocalisation, 3 min for Visual Attention, Touch R and Touch L, 4 min for Proximity, Body Orientation and Physical Play, and the full 5 min for Posture. Similarly, with this criterion, data for Cardiff Infants in Table 3 suggests coding: 3 min for Visual Attention, Posture, Touch R, Touch L and Vocalisation, and 4 min for Body Orientation and Physical Play. However, it is important to note that the threshold in this criterion is arbitrary and has been provided only as guidance for the purposes of interpreting these data. What constitutes an acceptable correlation is subjective, and researchers may wish to adjust the criterion according to their own research aims.

Given that we have identified correlations for individual slices, it would also be reasonable in some cases to select a specific slice for coding based on the correlations above. However, this would only be viable if the video procedures within the research followed the same process as ours (i.e., the 5 min session begins when the participant/researcher announces the beginning of an interaction, any remaining video past these 5 min are not included in the full session). Else, if the video procedures differed to our methodology (for example, selecting a 5-min video segment was chosen randomly out of longer video), it would not make sense to choose to code a specific slice based on the correlations in Tables 3, 4 and 5, because the beginning, middle and end portions of the video would not be representative of our beginning, middle and end portions. In this case, we would recommend using a criterion similar to our example above.

### Transition Matrices

**Fig. 2:**
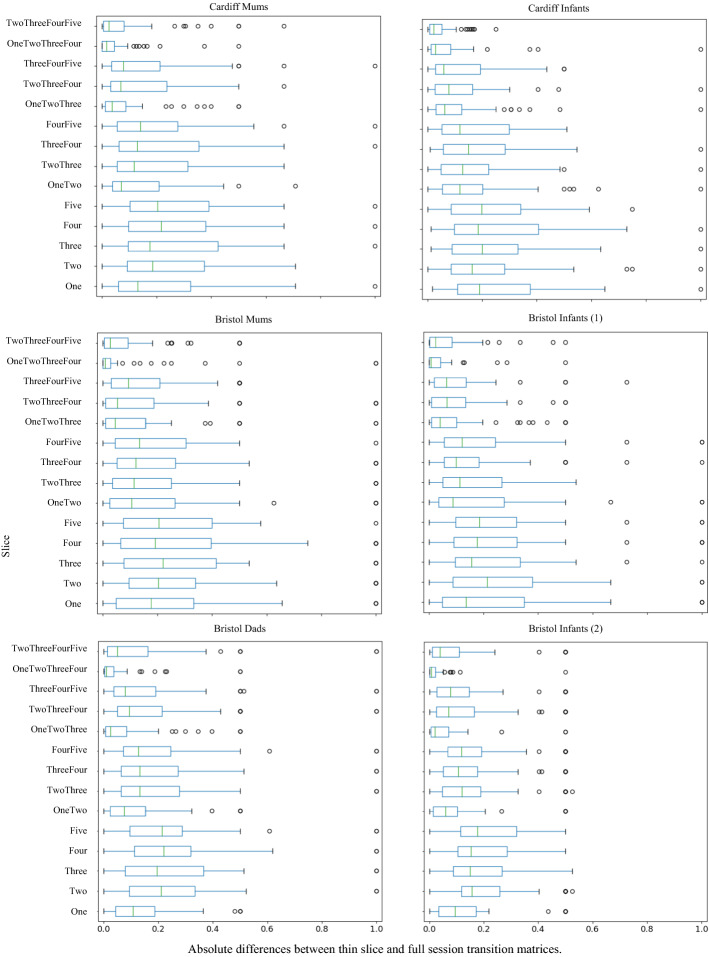
Box plots to show spread of absolute differences between transition matrices calculated at specified thin slice and full sessions. Average differences and anomalous data are indicated. From top-left to bottom-right: Cardiff Mothers, Cardiff Infants, Bristol Mothers, Bristol Infants (1), Bristol Dads and Bristol Infants (2). The boxes represent the upper and lower quartiles of difference values for the specified slice, the horizontal lines demonstrate the full spread of data, the centred, vertical lines show the median differences, and the circles represent anomalies (in this case, anomalies refer to the absolute differences between thin slice and full session transition matrices that were considerably higher than the mean)

For all behaviours and populations, transition matrices were calculated over the 1-, 2-, 3-, 4- and 5-min slices (where the 5-min slice *OneTwoThreeFourFive* is equivalent to the full session). The absolute differences between thin slice and full session transition matrices were calculated and averaged across all mothers/fathers/infants within the population. Then we plotted the absolute agreement between transition matrices at 1-, 2-, 3- and 4-min slices and those from the 5-min full session, separated by subject. Due to paper length constraints, this plot has been included in the “Transition Matrices” section of the “Appendix” (Fig. 2). From Fig. 2 we can interpret which coded slices generate transition matrices that are most similar to the full session transition matrices (i.e., the slices with a “left-most” distribution, with a median absolute difference close to 0). Note that absolute differences may only be between 0 and 1, as transitions are represented by probabilities.

Across all populations, Fig. 2 (of the “Appendix”) highlights a positive correlation between slice length and the absolute difference between transition matrices over thin slices and full sessions. To illustrate this, consider Cardiff Mums: the absolute difference between transition matrices over the fully coded interaction and the 1-min slice *One* was 0.131, the 2-min slice *OneTwo* is 0.071, the 3-min slice *OneTwoThree* is 0.037, and the 4-min slice *OneTwoThreeFour* is 0.018. Consistent with our hypothesis, these data suggest that transition matrices extracted from thin slices of coded data become more similar to those from fully coded interactions as slice length increases.

We can use these data to identify for which specific slices of a given length transition matrices were most similar to the full session equivalents. This gives us an idea of which slices are most representative of full sessions in terms of transitions between behaviours, and can help us to identify which slices to target (or not to target) for coding. For example, for the Cardiff Mums slices of length 1-min (slices *One, Two, Three, Four* and *Five*), we see that the smallest absolute difference between any 1-min slice and the full session (or the “left-most” median for 1-min slices) occurs in slice *One*. This means that the transitions between behaviours within the first minute (*One*) were most similar to behavioural transitions in the fully coded session, compared to the middlemost (*Two, Three, Four*) and final (*Five*) 1-min slices. Similarly for Cardiff Mums, we see that the most representative 2-min slice is slice *OneTwo*, the most representative 3-min slice is slice *OneTwoThree*, and the most representative 4-min slice was slice *OneTwoThreeFour* (these are the slices with the smallest median absolute difference). As such, this indicates that the earliest slices, regardless of length, were more representative of full-session transitions compared to the middlemost and later slices.

This pattern persists across many subplots within Fig. 2: slices taken from early in the interaction show lower absolute differences in comparison to later slices. One specific example is the 3-min slices for Bristol Mums: the mean absolute difference between the full session and slice *OneTwoThree* is 0.046, for slice *TwoThreeFour* is 0.055, and for slice *ThreeFourFive* is 0.096. The slice including the first minute (*OneTwoThree*) showed the lowest absolute difference. Conversely, we also observe that behavioural transitions within the latest slices (e.g., *Five, FourFive, ThreeFourFive*) are least representative of those within the full interaction. This pattern is almost consistent across samples (except for Cardiff Infants), but is particularly strong for Bristol Infants (1) and (2).

*Chi-squared Analyses*: Chi-squared analyses were performed to test the null hypothesis that as slice length increased, transition matrices would become more similar to those obtained by full sessions, for all behaviour categories. For these tests, transition frequencies were used as opposed to transition probabilities (similar to Friedman et al., 2010), and the degrees of freedom (df) were equal to the number of unique behaviours within each behavioural group.

The complete findings from these analyses are very large, so are provided in detail in the “Appendix” (Tables 13, 14, 15, 16, 17, 18). Similarly to within our frequency analyses, values within Tables 13, 14, 15, 16, 17 and 18 have also been made bold appropriately to highlight an example “criterion”, indicating “sufficient” similarity between transitions over a given thin slice and full session; specifically, where *p* < 0.05 for all slices of a given length. This criterion was chosen as the low *p* value indicates that the transitions within the given slice pairings are closely related.

In brief, these data indicate that transition matrices drawn from slices of the shortest length (1-min) are the least similar to the full session transition matrices, whilst transition matrices drawn from the longest slices (4-min) are the most similar. This is consistent with our hypothesis and is shown to be true across all populations and subjects. As an example, consider Bristol Mums transitions from Out of Reach (Table 15). Comparing the full, 5-min session and the 1-min slice *One*, we have χ^2^ (4, *N* = 15) = 33.64, *p* < 0.001; for the full session and the 2-min slice *OneTwo*, χ^2^ (4, *N* = 15) = 30.38, *p* < 0.001; for the full session and the 3-min slice *OneTwoThree*, χ^2^ (4, *N* = 15) = 19.93, *p* < 0.001; and for the full session and the 4-min slice *OneTwoThreeFour*, χ^2^ (4, *N* = 15) = 6.85, *p* > 0.05. In this example, we see that the χ^2^ value decreases as slice length increases, indicating the increased similarity between thin slice and full session transition matrices.

Additionally, comparing chi-squared analyses for individual slices allows us to again interpret which specific slices—or which portions of an interaction—contain transitions which best represent full interaction transitions. In terms how we interpret this from the given tables (Tables 13, 14, 15, 16, 17, 18), we look for the lowest χ^2^ value for a behaviour at a given slice length (this signifies the slice containing transitions most similar to full-session counterparts).

To illustrate this, consider Bristol Infants (1) Head Orientation behaviours over 1-min slices (rows 55–11, Table 16). For “vis-à-vis—infant and caregiver”, for example, we have that: for *One*, χ^2^ (4, *N* = 15) = 75.93, for *Two*, χ^2^ (4, *N* = 15) = 61.03, for *Three*, χ^2^ (4, *N* = 15) = 53.74, for *Four*, χ^2^ (4, *N* = 15) = 65.18 and for *Five*, χ^2^ (4, *N* = 15) = 69.36. In this case, the lowest χ^2^ value for 1-min slices, is for slice *Three*. This indicates that transitions from vis-à-vis in slice *Three* are most similar to transitions from vis-à-vis across the full interaction, compared to the equivalent measure in slices *One, Two, Four* and *Five*. We can find similar results for the other head orientation behaviours: for “Slight (30°–90°) aversion right”, slice *Four* is most similar, for “Slight (30°–90°) aversion left”, slice *Two* is most similar, etc. Looking at all of the Head Orientation behaviours together, we see that slices *Two* and *Five* most frequently have the lowest χ^2^ value. So if we wanted to look at transitions between Head Orientation behaviours and we only were only able to code 1-min of the interaction, then either of these would be the optimum choice. By the same argument (selecting the slice with lowest χ^2^ values), if we were to choose to code 2-min of Head Orientation then we should choose either slice *OneTwo* or *ThreeFour*, if coding 3-min we should choose either slice *TwoThreeFour* or *ThreeFourFive*, or if coding 4-min we should choose slice *TwoThreeFourFive.*

Similar interpretations can be drawn across all behaviours and slice lengths. We can observe that the specific slices that were most representative of full session transitions varied considerably depending on behaviour, and that even within the same behaviour, there was variation across population (we consider the reasons for this in more detail in the Discussion).

*Selecting Thin Slices Based on Transition Matrices*: For ease of translating these data into an applicable coding framework, appropriate values within Tables 13, 14, 15, 16, 17 and 18 have been made bold to exemplify a potential “criterion” for sufficient similarity between transitions over thin slices and full sessions, i.e. where *p* < 0.05 for all slices of a given length. Using this criterion, fully-boldened regions within these tables indicate the suitable coding timeframe for each behavioural group. For example, if we want to understand transitions between behaviours within a category, Table 15 suggests that coding 4 min is suitable for all caregiver behavioural groups except for Posture and Hand Movements, where the full 5-min of coding (or longer) is required. Similarly for Bristol Infants (1), Table 16 suggests coding 4 min for Body Orientation, 4 min for Visual Attention, and the full 5 min (or longer) for Head Orientation. However, it is important to note that whilst this criterion has been provided as guidance, an acceptable *p* value is subjective, and researchers may wish to adjust this criterion according to their own research aims.

Given that we have carried out chi-squared analyses for individual slices, it would also be reasonable in some cases to select a specific slice for coding based on the χ^2^ (and *p*) values provided. This would be done by choosing the slice containing the lowest χ^2^ values, compared to other slices of the same length. However, as before, this would only be viable if the 5-min coding segment was selected in the same way as within our work. Else, choosing a specific slice based on the lowest χ^2^ values would not make sense, as the beginning, middle and end portions of the video would not be representative of our beginning, middle and end portions. In this case, we would recommend using a criterion similar to our example above (*p* < 0.05 for all slices of a given length).

### Stationary Distributions

**Fig. 3:**
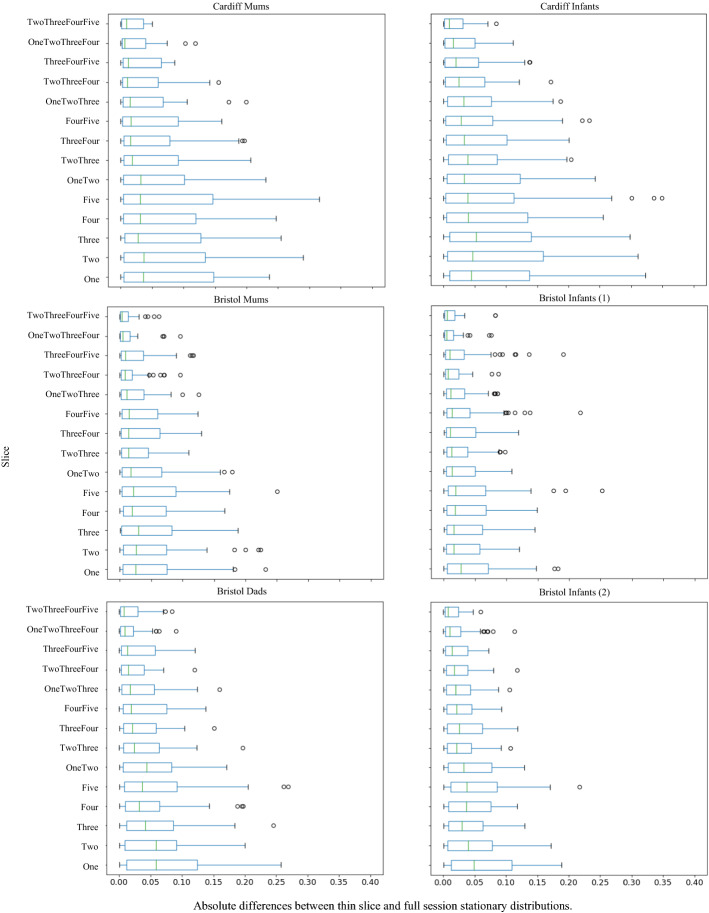
Box plots to show spread of absolute differences between stationary distributions calculated at specified thin slice and full sessions. Average differences and anomalous data are indicated. From top-left to bottom-right: Cardiff Mothers, Cardiff Infants, Bristol Mothers, Bristol Infants (1), Bristol Dads and Bristol Infants (2). The boxes represent the upper and lower quartiles of difference values for the specified slice, the horizontal lines demonstrate the full spread of data, the centred, vertical lines show the median differences, and the circles represent anomalies (in this case, anomalies represent absolute differences between thin slice and full session stationary distributions that were much higher than the mean)

Using the transition matrices, corresponding stationary distributions were calculated for all slices over each population and behaviour. The absolute differences between thin slice and full session stationary distributions were calculated, and averaged across all mothers/fathers/infants within the population. Then we plotted the absolute agreement between stationary distributions at 1-, 2-, 3- and 4-min slices and those from the 5-min full session, separated by subject. Due to size constraints, this plot has been included in “Stationary Distributions” section of the “Appendix” (Fig. 3). From Fig. 3, we can interpret which coded slices obtain stationary distributions that are most similar to the full session stationary distributions (i.e., the slices with a “left-most” median absolute difference, close to 0). Note that absolute differences range between 0 and 1, as stationary distribution values are probabilities.

Across all populations, this plot highlights a positive correlation between slice length and the absolute difference between stationary distributions at thin slices and full sessions. As an example, consider Cardiff Infants: the absolute difference between stationary distributions over the fully coded interaction (*OneTwoThreeFourFive*) and the 1-min slice *One* is 0.044, the 2-min slice *OneTwo* is 0.033, the 3-min slice *OneTwoThree* is 0.032, and the 4-min slice *OneTwoThreeFour* is 0.016. Consistent with our hypothesis, these data suggest that stationary distributions extracted from thin slices become more similar to those from fully coded sessions as slice length increases.

Figure 3 (of the “Appendix”) emphasizes that absolute differences in the Cardiff Mums population showed higher variation compared to the Bristol populations. This is likely due to differences in frequencies; particularly, Tables 7, 8 and 9 above showed how behavioural frequencies were highest in Cardiff Mums and Infants compared to the other subjects. These higher frequencies may have caused higher absolute differences between thin slices and full sessions.

We can also use these data to compare results between slices of the same length, in order to identify which slices to target (or not to target) for coding. For each slice length, we can identify within which specific slice the stationary distributions were most similar to the full session equivalents. For example, for the Bristol Mums slices of length 1-min (slices *One, Two, Three, Four* and *Five*), we can see that the lowest absolute difference between any 1-min slice and the full session (or the “left-most” median for 1-min slices) occurs in slice *Four*. This means that the time spent within each behavioural state during the fourth minute were most similar to behavioural transitions in the fully coded session, compared to the other 1-min slices. As slice length increases, it becomes harder to distinguish which slice shows the lowest absolute difference, as all differences are very small and similar in value. This is true for many of the populations for slices longer than 2 min, and suggests that as slice length increases, it is less important which specific slice is chosen for coding.

*Chi-Squared Analyses*: Chi-squared analyses were performed to test the null hypothesis that as slice length increased, stationary distributions would become more similar to those obtained by full sessions, for all behaviour categories. In these tests, the degrees of freedom (df) were equal to the number of unique behaviours within each behavioural group.

The complete findings from these analyses are very large, so are provided in the “Appendix” (Tables 19, 20, 21, 22, 23, 24). Similarly to above, appropriate values within Tables 19, 20, 21, 22, 23 and 24 have also been made bold to highlight an example “criterion”, indicating “sufficient” similarity between stationary distributions over the thin slice and full session; specifically, where *p* < 0.05 for all slices of a given length. This criterion was chosen as the low *p* value indicates that the stationary distributions within the given slice pairings are closely related.

In sum, these data demonstrate that stationary distributions from slices of the shortest length (e.g., *One*, *Three*) were least similar compared to those from the fully coded interactions, whilst stationary distributions from the longest slices (e.g., *OneTwo-ThreeFour*) were most similar. Consistent with our hypothesis, this means that the probabilities of remaining within behavioural states as predicted by thin slices become more similar to full session probabilities as slice length increases. As an example, consider the Bristol Infants (2) stationary distribution for Touch left Hand (Table 24). Comparing distributions for the full 5-min session and the 1-min slice *One*, we have χ^2^ (3, *N* = 15) = 36.27, *p* < 0.001; for the full session and the 2-min slice *OneTwo*, χ^2^ (3, *N* = 15) = 9.19, *p* < 0.05; for the full session and the 3-min slice *OneTwoThree*, χ^2^ (3, *N* = 15) = 1.75, *p* > 0.05; and for the full session and the 4-min slice *OneTwoThreeFour*, χ^2^ (3, *N* = 15) = 0.54, *p* > 0.05. Here we see that the χ^2^ value decreases as slice length increases, indicating the increased similarity between thin slice and full session stationary distributions.

Additionally, comparing chi-squared analyses for slices of a given length allows us to identify which specific slice—or which portion of an interaction—should be targeted for coding. In terms how we interpret this from the given tables (Tables 19, 20, 21, 22, 23, 24), we look for the lowest χ^2^ value for a behaviour at a given slice length (this signifies the slice containing stationary distributions most similar to full-session counterparts).

To illustrate this, consider Bristol Dads Body Orientation behaviours over 1-min slices (Table 23 of the “Appendix”). We have that: for slice *One*, χ^2^ (4, *N* = 17) = 9.14, for *Two*, χ^2^ (4, *N* = 17) = 44.92, for *Three*, χ^2^ (4, *N* = 17) = 1.04, for *Four*, χ^2^ (4, *N* = 17) = 4.55, and for *Five*, χ^2^ (4, *N* = 17) = 31.09. The lowest χ^2^ value for 1-min slices is for slice *Three*. This means that the long-run proportions of time spent within Proximity behaviours are best approximated by slice *Three*, as opposed to slices *One, Two, Four* and *Five*. So if we wanted to analyse the durations of proximity behaviours, and were only able to code 1-min of the interaction, then slice *Three* would be the optimum choice. By the same argument (selecting the slice with lowest χ^2^ value), if we were to choose to code 2-min of Proximity then we should choose slice *FourFive*, if coding 3-min we should choose slice *ThreeFourFive*, or if coding 4-min we should choose slice *TwoThreeFourFive.*

Similar interpretations can be drawn across all behaviours and slice lengths. Comparing results across populations, we see that the lowest χ^2^ values are most commonly derived from the middlemost and end slices, as opposed to the first slices of the interaction (particularly true as slice length increases). Potential reasons for this trend are detailed within the discussion.

*Selecting Thin Slices Based on Stationary Distributions*: For ease of translating these data into an interpretable coding framework, appropriate values within Tables 19, 20, 21, 22, 23 and 24 have been made bold to exemplify a potential “criterion” for sufficient similarity between stationary distributions over thin slices and full interactions, i.e. where *p* < 0.05 for all slices of a given length. Particularly, the bold values within these tables indicate the suitable coding timeframe for each behavioural group. For example, if our aim is to understand the prediction of long-term behavioural states, Table 23 (Bristol Dads) suggests coding 1 min for Posture, 2 min for Visual Attention, and 3 min for Vocalisations. Similarly, Table 24 (Bristol Infants (2)) suggests coding 2 min for Head Orientation, 3 min for Physical Play, and the full 5 min (or longer) for Facial Expressions. However, it is important to note that this criterion has been suggested only as guidance, and choosing an acceptable *p* value is subjective, so researchers may wish to adjust this criterion according to their own research aims.

As before, the chi-squared analyses for individual slices means that specific slices could be for coding based on the χ^2^ (and *p*) values provided, but only if video recording and coding followed the same procedures as ours.

## Discussion

### Summary of Results

Behavioural coding can be challenging, time intensive and laborious. Researchers’ struggles with this process can be intensified by many factors, including long coding intervals, complex coding schemes with multiple behaviour categories, or the implementation of novel coding schemes (Pesch & Lumeng, 2017). Thin slice coding offers an alternative approach which aims to alleviate the coding burden. However, there is not yet a strong conclusion on whether behavioural features coded over thin slices of an interaction are representative of the full session, particularly for parents and infants. By comparing frequencies, transition matrices and stationary distributions for a range of behaviours over thin slices and fully coded observations, this work aimed to provide quantitative evidence for the value of thin slice coding in parent-infant interactions.

We hypothesised that as slice length increased, behavioural frequencies, transition matrices, and stationary distributions would become more similar to the full session equivalents. Our results were consistent with this hypothesis, across all populations, subjects, and behaviours. Particularly, we found stronger correlations between frequencies of behaviours over thin slices and fully coded interactions as the length of the slice increased; the strongest correlations occurred between 4-min slices and full sessions, and the weakest correlations occurred between 1-min slices and full sessions. Some behaviours demonstrated worse correlations than others. Additionally, we used an example criterion of r > 0.9 to indicate a “standard” strength of association, highlighting the most closely related slices.

Further, using absolute differences and chi-squared analyses, we showed that transition matrices drawn from thin slices became more similar to those from full sessions as slice length increased, i.e., transitions from one behaviour to another were most accurately predicted by longer slices. Similarly, we showed that stationary distributions drawn from thin slices became more similar to those from fully coded interactions as slice length increased, i.e., long term behavioural states were most accurately predicted by longer slices. For both transition matrices and stationary distributions, we used an example criterion of *p* < 0.05 for all slices of a given length to indicate a “standard” sufficient similarity between slices, highlighting those that were most closely related.

These findings are consistent with those of both Murphy (2005) and Carcone et al. (2015), who found higher correlations between frequencies of coded behaviours over thin slices and fully coded interactions as the lengths of slices increased. Whilst these studies utilised full sessions of length 15- and 30-min, respectively, we have reproduced similar results for full sessions at a shorter length of 5-min. Our results are also consistent with those of James et al. (2012), who found that behavioural measures over short portions of interactions (3- and 6-min) were significantly different from full sessions (18-min). We have found this to be true for transitions between behaviours and the prediction of long-term states, specifically when comparing 1- and 2-min slices to the full, 5-min interactions (Tables 13, 14, 15, 16, 17, 18, 19, 20, 21, 22, 23, 24 of the “Appendix”). This is in line with Holden and Miller (1999), who emphasized that brief assessments of parenting practices can reflect characteristics that endure over time.

### Suitability of Thin Slice Sampling

This work contributes further understanding to the instances in which thin slice sampling is an effective, suitable practice for reducing the coding burden. The detail we have provided on specific behaviours, populations and measures means that results can be tailored to individual studies, and researchers can use the correlations and agreements provided to choose for themselves the level of accuracy that they deem acceptable for their own research aims. As such, we separate the following discussion into points (1)–(5) below, each addressing a different factor that may affect the suitability of thin slice sampling in practice.

1. *Behaviours of Interest*: Murphy et al. (2019) suggested that future work should examine what kinds of behaviours are more and less suitable for thin slice analysis. We have aimed to do this by incorporating as many behaviours as possible into our coding methodology. In comparison to previous literature in thin slice sampling, we coded a large number of behavioural groups and individual behaviours. Previously, some have focused on a single type of behaviour (e.g., Carcone et al. (2015) and Roter et al. (2011) both focused only on verbal communication behaviours). Others have coded behaviours across multiple behavioural groups (e.g., Murphy (2005) and Wang et al. (2020) each focused on a combination of five, distinct non-verbal and vocal behaviours, such as ‘lean in’ or ‘nod’). In a coding scheme most similar to ours, Hirschmann et al. (2018) coded multiple behavioural groups that were each comprised of several mutually exclusive behaviours. By including as many behaviours as possible in our analysis, we believe that our behaviour-specific findings may inform future studies across a variety of contexts.

However, whilst including a large range of behaviours ultimately allowed us to assess many different contexts for thin slice sampling, we acknowledge that this has led to the analyses including some specific scenarios which may not well address the research question. For example, knowing infant posture is very useful for providing both physical and social context to a parent-infant interaction (e.g., infant posture is linked to gazing at mothers face (Fogel et al., 1999)), yet, for infants of aged 6–7 months (as within this work), there is unlikely to be much self-led variation in this behaviour. Of course, parents will likely adjust the infants position throughout the interaction, but this is likely to significantly vary dependant on the interaction type and physical setting. Another example is the presence of “Physical Play” within bedtime, reading or feeding interactions, where there is likely to be very little of this behaviour. The inclusion of this scenario is likely to have led to inflated correlations for “Physical Play” in Tables 8 and 9 (only for Bristol Mums and Dads).

Within the “Results” section, we have demonstrated how to extract suitable coding timeframes for specific behaviours from the tables provided, according to individual behavioural measures. Here we give a full example of this process, encompassing all extracted measures for one behavioural group: Caregiver Vocalisation. For simplicity, we will only consider measures obtained from the Cardiff Mums population. Firstly, Table 7 (of the “Appendix”) shows the frequency of Caregiver Vocalisation behaviours across all slices. We can see how the most vocalisations occurred in early slices *One* and *Two*, and the least vocalisations occurred in the later slices *Four* and *Five*. Table 7 (in the “Results” section) shows the correlations between Vocalisation frequencies over 1-, 2-, 3- and 4-min slices compared to the full session. For each slice length, we can see for which specific slice the vocalisations best correlate with the full session (for 1-min slices, slice *One* best correlates; for 2-min slices, slices *TwoThree* and *ThreeFour,* for 3-min, slice *TwoThreeFour*; for 4-min, slice *TwoThreeFourFive*). We can also see that r > 0.9 for all slices of length 2-min or longer; using our criterion of r > 0.9, this suggests that coding 2 min of Vocalisations would be sufficient for representing full session frequencies.

In terms of transitions between individual vocalisation behaviours, using our example criterion of *p* < 0.05 for sufficient similarity, the bold values within Table 13 (of the “Appendix”) show that transitions from multiple vocal states (e.g., Musical Sounds, Nervous Laugh) predicted by 1-min met this criterion. By 3 min, transitions from Speech met the criterion, and by 4-min slices, all transitions from any vocal state met the criterion. This suggests that 4 min is a suitable coding timeframe for representing full session transitions between vocalisation behaviours (as indicated by the fully-boldened region in Table 13).

Finally, using our example criterion of *p* < 0.05, the bold values in Table 19 (of the “Appendix”) shows that Vocalisation stationary distributions from 3- and 4-min slices met the criterion, i.e., distributions from these slices were suitably similar to the distribution from the full 5-min session. This suggests that in terms of long-term behaviour prediction, 3 min of coding is a suitable coding timeframe in order to represent the full 5 min interaction.

We have outlined many examples of where our findings indicate that coding time of a behavioural group could be largely reduced; however, this was not always the case. To illustrate this, consider Caregiver Posture for Bristol Mums. The frequencies of this behaviour were extremely low compared to other behaviours (for 1-min slices, frequencies ranged from 0.73 to 1.27, see Table 9 of the “Appendix”). Consequently, correlations between frequencies over thin slices and full sessions were low compared to other behavioural groups (e.g., Posture was the only behaviour to have r < 0.9 for any 4-min slice, see Table 3 in the “Results” section), and transition matrices from many thin slices—even over the longest, 4-min slices (e.g., see Sit on object, and Sit on floor, Table 15 of the “Appendix”)—did not meet our example criterion (*p* < 0.05). Together, these findings suggest that the full 5-min session should be coded for Caregiver Posture, as coding for a reduced amount of time did not generate the same outcome measures as the fully coded interaction. In this case, thin slice sampling was not a suitable practice.

Roter et al. (2011) suggested that further understanding of the efficacy of thin slice sampling would “…help researchers decide on the trade-offs entailed in adopting this methodology in their own studies”. We believe that our contribution addresses this question. By incorporating a large variety of behavioural groups into our coding scheme, our findings have provided new knowledge regarding specific instances in which thin slice sampling is an effective, suitable practice for reducing the coding burden. We have shown this suitability to be dependent on the behavioural group of interest.

2. *Type of Behavioural Measure*: Our analyses have employed three separate behavioural measures—frequencies, transition matrices and stationary distributions—and each of these measures represents a different aspect of behaviour. Frequencies calculate how often a behaviour occurs within a period of time; transition matrices identify the number of times that a parent/infant transitions between two behaviours; and stationary distributions tell us about the long-run proportion of time spent engaging in (or duration of) a given behaviour. It is therefore unsurprising that the efficacy of thin slice sampling is linked to the chosen measure.

For example, we have seen that correlations between thin slice frequencies and full sessions were “strong” or “very strong” for most behaviours, even at short slices, suggesting that thin slice sampling of 1- or 2-min slices may be suitable for some behaviours (e.g., Bristol Mums Body Orientation and Proximity, Bristol Infants (2) Physical Play and Hand Movements). However, chi-squared analyses for many behavioural groups revealed that transitions predicted by thin slices did not meet the example criterion, and were therefore not considered representative of transitions within the fully coded sessions. This suggests that thin slice sampling of 1- or 2-min slices may not always be suitable for accurately calculating transitions between behaviours. In this way, it is up to researchers to make their own decisions regarding the suitability of this practice, dependent on the type of behavioural measures applied in their methodologies.

3. *Subjectivity of Interpretation*: It is important to note that implications of our findings are also dependent on researchers’ personal perceptions of what level of agreement they deem “acceptable”, for example, the necessity of a “very strong” (r > 0.9) or “strong” (r > 0.7) Pearson correlation.

Throughout our work, however, we have provided detailed guidance for how researchers may choose an appropriate thin slice, depending on the behaviour and measure. We have chosen an “acceptable” correlation of frequencies of r > 0.9, and an “acceptable” similarity between transitions/stationary distributions indicated by *p* < 0.05 for all slices of a given length. With these criteria, we have found many examples where 2, 3 or 4 min of coding is an acceptable proxy for 5 min, depending on the behaviour, population and measure. However, whilst we have used these criteria as a baseline for our own data interpretations, we recognise that the levels of agreement chosen here may not be suitable for all research aims, and that other researchers may wish to adapt these criteria accordingly.

4. *Physical Context of an Interaction*: The three populations used within our work encompass some differences in physical context for the interactions. Namely, some videos were collected in-lab (Cardiff Mums), and others at home (Bristol Mums and Dads). This key difference has, unsurprisingly, been reflected in our findings, and is an important factor to consider as it demonstrates that videos recorded at home vs. in-lab may require different thin slice sampling decisions to be made.

One such difference between these populations is that the Bristol interactions varied in type (e.g., eating, playing, reading), whereas all Cardiff interactions were classed as “free play”. This is likely to have contributed to greater variation in behaviours within the population (as reflected in higher standard deviations within Table 3 of the “Results” section). Also, the use of headcams for recording mother-infant interactions at home has been linked to less socially desirable behaviours occurring in mothers (Lee et al., 2017), which may account for any differences between Bristol and Cardiff Mums. The Cardiff Mums population also has an unusually high proportion of mothers with postgraduate degrees (38.7%), and mothers are around 8–10 years older than the Bristol Mums and Dads (35.4 years compared to 24.4 and 27.5, respectively). Finally, for the Bristol Dads videos, fathers often started the video recording by introducing the activity and vocalising the time and date. These introductions took place within the first minute of coding, which may account for some differences between at-home/in-lab findings (and also the differences between slices including minute *One* and those that do not, in Fig. 2 of the “Appendix”).

Given the multiple potential reasons for variation of behaviours captured within in-lab and at-home data collection, it is not surprising that these differences have been reflected in our findings. As an example, comparing Cardiff findings to those from both Bristol Mums and Dads in correlation Table 3 (in the “Results” section), we observe differences in the correlations between behavioural frequencies over thin slices and full sessions. Correlations in the Cardiff population were generally not classed as “very strong” (r > 0.90) for the earlier slices, and correlations remained much lower than the equivalent values for the Bristol Mums and Dads population. Comparatively, correlations in both Bristol populations were often “very strong” at 1- or 2-min slices. Another example of differences between Cardiff and Bristol findings is the “cut off” for accurately predicting behavioural transitions from a thin slice (Tables 13, 14, 15, 16, 17, 18 of the “Appendix”); these data suggest that 4 min is suitable for Bristol Mums and Dads, whereas a decreased slice length of 3 min is suitable for Cardiff Mums.

Therefore, we can see how the suitability of thin slice sampling varies between populations, depending on whether video recordings were taken in-lab or at-home. We recommend that researchers should consider these differences in the future if choosing to implement a thin slice sampling methodology.

5. *Portion of the Interaction*: Given the choice to code a specific number of minutes of an interaction, researchers will likely want to choose to code a slice from the most representative portion—the beginning, middle or end.

The findings for the Cardiff Mums population can be used to interpret which portion of an interaction—the beginning, middle, or end—is most representative of the full interaction, in terms of frequency and duration of behaviours, and the transitions between them. Whilst these exact interpretations cannot be drawn from the Bristol populations, given the methods of video coding, we can however observe differences between earlier slices (equivalent to the beginning portion of the interaction) and the remaining slices (equivalent to the “rest” of the interaction, including middle and end portions).

Looking first at our frequency correlation results in Table 3 in the “Results” section, for all populations and across different slice lengths, the prevalent pattern was that the largest correlations were found for the middlemost slices (e.g. *TwoThreeFour*), compared to the earliest (e.g., *OneTwo*) and final slices (e.g., *FourFive*). Examples of this include: Bristol Dads Proximity and Facial Expression; Bristol Mums Touch R and Posture; and Cardiff Mums Touch L and Visual Attention. Importantly, this trend was stronger in the parent samples compared to the infants. As slice length increased, we found that correlations were most often highest in the earliest slices, and this was true regardless of population and sample. In contrast, correlations were often lowest in the latest slices.

For transitions between behaviours, we found that the earliest slices, regardless of length, were most representative of full-session transitions compared to the middlemost and later slices (this is shown in Fig. 2 of the “Appendix”). Further, the end-most slices were often the least representative. For stationary distributions, there was no clear pattern linking slice position to how representative it was of the full session (this was shown in Fig. 3 of the “Appendix”).

There are many potential reasons for these observed differences between behaviours within the beginning, middle and end of the interactions. From the parent perspective, it could be that the parents were aware of being filmed, so acted more “performatively” for the camera as the recording started (but dropped this behaviour as the interaction persisted). The parents may also have been more fidgety early on, if they were feeling nervous about the recorded task, or if they were taking time to set up a toy/food for the session (something we did observe in the home based videos). Finally, parents may have been more engaged in the beginning of the interaction, but lost interest over time. All of these example scenarios would contribute to a higher frequency of behaviours early in the interaction, compared to the middle and end, affecting how reflective the beginning slices are of the full interaction, compared to other slices.

It is not surprising that the differences between early and later slices are more pronounced in the parent data compared to the infant data. As infants are too young to comprehend that they are being recorded, they would not consciously (or subconsciously) adapt their behaviour in any way. This could partly explain why this pattern is not strongly present in the infant behaviour frequency correlations (Tables 3, 4, 5 in the “Results” section). However, it is likely that infant behaviour is reflective of parents behaviour in some regard, for example, infants may become more fidgety (i.e., a higher frequency of behaviours) when the parent is taking some time to start feeding or set up a toy. This would explain why differences between early and later slices still occur within the infant data, but are just less pronounced. At this stage we are only speculating about potential correlations in dyadic behaviours, however, this is something we intend to explore in detail in our future work.

Finally, we have also seen that the latest slices were often least representative of full session behaviours. While this may in part be explained by a drop in parent interest as the interaction persists, we also suggest that coder fatigue may be a contributing factor. In particular, coding a 5 min interaction is tiresome and takes a long time (in our experience, roughly 5 min to code one minute of a single behaviour), so it is plausible that human coders begin to code less accurately as the interaction progresses, perhaps by neglecting to code some briefly occurring behaviours. This would result in lower frequencies of behaviours (and subsequently, transitions between them), meaning that the final slice becomes much less comparable to the full interaction.

These interpretations should be treated with caution, as our coding methods for the Bristol Mums and Dads populations ensured that the “end” of the physical interaction was not always captured within the coded video that we analysed. We can, however, still make reasonable comparisons between the beginning slices and the middle/end slices for all populations.

### Implications for Parent–Infant Research

We have built upon existing literature that applied thin slice sampling to mother-infant interactions (e.g., Hirschmann et al., 2018; James et al., 2012). Whilst previous work has focused on behavioural contingencies and cooccurrences (James et al., 2012), we have contributed to the understanding of transitions between behaviours and prediction of long term-states. Although our primary focus differed from that of James et al. (2012), putting findings into sociological context demonstrates their comparability: James et al. (2012) used behavioural measures to represent parent response to infant behaviour (“interactive contingency”, see Beebe et al., 2010), whilst we used transition matrices and stationary distributions to represent parent or infant response to their own behaviour (“self-contingency”, see Beebe et al., 2010). Each of these measures are important in how infants learn to predict and develop control of events, developing their understanding of behavioural sequences in themselves, their parents, and within the dyad (Beebe et al., 2010).

By coding similar behaviours for both mothers and infants, we have been able to see how behavioural frequencies compare between the two subjects. We identified some behaviours that occurred more frequently in parents than in infants, e.g., Vocalisation, Touch R, and others that occurred more frequently in infants than parents, e.g., Body Orientation, Head Orientation. Despite these differences, we did not find evidence that correlations between frequencies of maternal behaviours over thin slices and full sessions were any stronger or weaker than those for infants. Further, Fig. 3 (in the “Appendix”) showed that the thin slice stationary distributions most similar to full session equivalents were obtained from the same slices for mothers and infants within the same population (*OneTwoThreeFour* for Cardiff Mums and Infants, and *TwoThreeFourFive* for Bristol Mums and Infants (1)). This suggests that researchers practicing thin slice sampling for mothers and infants could code the same slice for each subject, whilst still obtaining the optimal prediction of long-term behavioural states.

At the time of this study, we had not identified any previous literature focusing on thin slice sampling for father-infant interactions. Our work has contributed new evidence in this area. We have shown that maternal and paternal behaviours in parent-infant interactions are often comparable. For example, we found similarities in the behavioural frequency correlations between Bristol Mums and Bristol Dads (Table 3); Caregiver Posture showed moderate correlation between 1- and 2-min slices and full sessions, Caregiver Head Orientation showed consistent strong or very strong correlations at all thin slices, and many other behaviours showed very strong correlations for all slices of 2-min in length (and longer). Also, in the stationary distribution analyses, Fig. 3 (of the “Appendix”) demonstrated very similar patterns in absolute differences for Bristol Dads and Bristol Mums, e.g., slice *TwoThreeFourFive* was most similar to the full sessions for both. We did not detect any notable differences between behavioural measures or statistical analyses obtained from mother-infant populations and father-infant populations.

### Strengths

A strength of this research is the inclusion of parent-infant interactions recorded at home. It has been suggested that using first person headcams within “real life” settings may capture more ecologically valid behaviours (such as less socially desirable maternal behaviours), and reduce demand characteristics in participants (Lee et al., 2017).

As previously mentioned, we have used a comprehensive range of both parental and infant behaviours for our analyses. We believe that this is a strength of this work, as we have begun to answer the question posed by Murphy et al. (2019) regarding which specific behaviours are more and less suitable for thin slice sampling.

Finally, we were unable to identify any previous studies investigating thin slice sampling for father-infant interactions specifically. By including fathers in our work—alongside mothers—we have provided new insights into father-infant interactions and how they compare to mother-infant interactions.

### Limitations

A potential limitation of this work is that some dyads provided multiple videos for analysis. Particularly, one Bristol mother provided two videos, three Bristol fathers provided two videos, and one Bristol father provided four videos. We were aware that this repetition could lead to behaviour biases and inflate correlations within the populations. In response, we ran all of the analyses twice—once with only one video per dyad, and once including all the videos (including the second, third and fourth videos for some dyads). Upon observing no differences in trends between the two sets of results, we ultimately decided to keep the analyses comprised of the most videos. Using all available data maximises power and precision of estimates, which is in the best interest of our work. Additionally, we suggest that biases and inflated results would have been reflected in differences between the Bristol and Cardiff populations, as no Cardiff mothers provided multiple videos. Whilst there were differences between the Cardiff and Bristol findings, we believe these were more likely attributed to variation in the interactions themselves (i.e. free play vs. eating/reading/bedtime) and in-lab vs. at-home recording procedures.

A further limitation is the potential selection biases present in the cohort studies. Multiple of these biases in the ALSPAC cohorts have previously been outlined in detail by Lawlor et al. (2019), for example, participants are mostly of White-European origin (particularly in -G2), which reduces the generalisability of findings to the general population. Similarly, 38.7% of mothers from the Cardiff Mums population held a postgraduate degree, compared to around 1% of the UK population (Higher Education Statistics Agency, 2021). However, whilst the populations used here may not be generalisable, findings may be reflective of contemporary families who are part of representative birth cohorts.

Our analyses were limited by the nature of the coding scheme and by the behaviours chosen (Costantini et al., 2021). For example, our coding approach generally does not consider the intensity or sentiment direction of behaviours (the only time this is considered is for “Vocalisation” codes, which includes an optional modifier to code “Positive”, “Neutral” or “Negative” tone). While intensity can be derived to some extent from the frequency of behaviours over a given unit of time, we felt that detailed analyses of this kind were out of the scope of our work, and would therefore serve as a sensible future direction of thin slice sampling.

Our analyses were also limited by the length of the coded interactions. Whilst previous work suggests that 5 min is enough time to constitute as a full session in thin slice sampling (Ambady & Rosenthal, 1992; Murphy, 2007; Murphy et al., 2019), it is plausible that a longer full session would provide new and different insights to those in our work. Unfortunately, as the data used in this work had already been collected and pre-processed for earlier projects (with different aims), most of the interactions were around 5 min in length anyway, and extending our work to interactions of 10 or 20 min would have massively reduced number of viable videos for us to include. However, we do believe that a future analysis comprising 10-, 15- or 20-min videos would be invaluable for understanding the implications of thin slice methodologies.

Finally, there were also instances of missing data in this study. This occurred for two reasons: (1) not all participants provided demographic information (e.g., education), and (2) the option to code “Not possible to code X behaviour”. “Not Possible to Code” was a necessary inclusion as sometimes specific behaviours cannot be identified. This may be due to poor camera footage—especially when videos are taken at home—or because participants move to face away from the camera. It is possible that a more “complete” dataset could reveal different results.

### Future Work

We have provided evidence that coding of many behaviours (e.g., vocalisations, facial expressions) can be reduced, whilst still drawing the same conclusions from the thin slice of coded data. We have also begun to evaluate how different portions of an interaction (the beginning, middle and end) may be more or less representative of the full session. However, given our coding methods, we were unable to fully explore the scope of this question and it’s interpretations (particularly, the “end” of the videos we used were not always representative of the end-most point of the physical interaction). It would therefore be beneficial to provide further evidence for this aspect of thin slice sampling, to help researchers to better understand which portion to prioritise for coding. It would also be useful to see more analyses of this type across different contexts, as the most representative portion is likely to vary significantly depending on the subjects of the interaction, the type of interaction, and the behaviours of interest.

Further, whilst we have seen thin slice sampling used for predictive validity of outcome variables in mother-infant interactions (Hirschmann et al., 2018; James et al., 2012), it would be beneficial to see similar investigations realised for father-infant interactions.

Finally, in a slightly different but related direction, future work that we are planning using this dataset will investigate correlations in dyadic behaviours. This work will include investigating links between simultaneously occurring and time-lagged behaviours, both within and across different behavioural groups.

## Data Availability

The data that support the findings of this study are available from both the Avon Longitudinal Study of Parents and Children (http://www.bristol.ac.uk/alspac/) and The Grown in Wales Study; but, restrictions apply to the availability of these data, which were used under license for the current study, and so are not publicly available. However, data are available from ALSPAC upon approval from the ALSPAC Executive Committee, and data from the Grown in Wales Study are available upon reasonable request from the GiW Study chief investigator Professor Rosalind M John.

## Appendix

### Analysis of Multiple Videos per Subject

Here, we describe an additional analysis that we carried out in order to ascertain whether correlations were inflated due to re-using multiple videos from the same subjects. We wanted to include as many subjects and videos as possible in the interest of power to our study, so here we wanted to evaluate whether this would lead to any biases in our results.

For this, we re-calculated the Pearson correlations in Tables 3, 4 and 5 of the main work (based on frequencies of behaviours), but instead using a dataset comprised only of single videos per mother/father/infant (i.e., second/ third/ fourth videos were removed completely). This totalled 14 videos for the Bristol Mums population, and 11 for Bristol Dads (there were no multiple videos in the Cardiff Mums population).We then carried out chi-squared analyses on these two equivalent datasets; these were computed such that all behaviours for slice One were compared across the two datasets, behaviours across slice Two were compared, etc. This was repeated for each of the 15 slices and for each of the four subjects (Bristol Mums mothers, Bristol Mums infants, Bristol Dads fathers and Bristol Dads infants), meaning that 14 chi-squared tests were completed in total. These results and the associated *p* values are presented below in Table 6.

**Table 6:** Chi-squared test on correlations in repeated and non-repeated subjects

Slice	Population and subject			
	Bristol mums Mothers	Bristol mums Infants	Bristol dads Fathers	Bristol dads Infants
*One*	0.56 (> 0.99)*	0.03 (> 0.99)	0.03 (> 0.99)	1.27 (> 0.99)
*Two*	0.28 (> 0.99)	0.00 (> 0.99)	0.09 (> 0.99)	1.57 (> 0.99)
*Three*	0.05 (> 0.99)	0.33 (> 0.99)	0.03 (> 0.99)	1.85 (.99)
*Four*	0.09 (> 0.99)	0.25 (> 0.99)	0.31 (> 0.99)	1.54 (> 0.99)
*Five*	0.05 (> 0.99)	0.09 (> 0.99)	0.02 (> 0.99)	1.85 (.99)
*OneTwo*	0.37 (> 0.99)	0.00 (> 0.99)	0.01 (> 0.99)	0.64 (> 0.99)
*TwoThree*	0.03 (> 0.99)	0.00 (> 0.99)	0.03 (> 0.99)	1.39 (> 0.99)
*ThreeFour*	0.03 (> 0.99)	0.07 (> 0.99)	0.03 (> 0.99)	1.67 (> 0.99)
*FourFive*	0.03 (> 0.99)	0.01 (> 0.99)	0.01 (> 0.99)	1.62 (> 0.99)
*OneTwoThree*	0.03 (> 0.99)	0.00 (> 0.99)	0.01 (> 0.99)	0.64 (> 0.99)
*TwoThreeFour*	0.05 (> 0.99)	0.00 (> 0.99)	0.03 (> 0.99)	1.26 (> 0.99)
*ThreeFourFive*	0.03 (> 0.99)	0.01 (> 0.99)	0.01 (> 0.99)	1.46 (> 0.99)
*OneTwoThreeFour*	0.00 (> 0.99)	0.00 (> 0.99)	0.02 (> 0.99)	0.65 (> 0.99)
*TwoThreeFourFive*	0.04 (> 0.99)	0.00 (> 0.99)	0.01 (> 0.99)	1.09 (> 0.99)

**P* values are shown in parentheses

These results shows that all *p* values were equivalent to 0.99 or higher, meaning that the differences between correlations in the multiple video dataset and the single video dataset were very small, and that the two datasets were considerably similar. There was therefore no evidence to indicate that including multiple videos inflated our correlations. So, in the interest of including all available observations and maximising statistical power to the analyses, we decided to include the multiple observations for single subjects where these existed.

### Markov Chain Analyses Walkthrough with Example

Here, we provide an in-depth example of how Markov chains, transition matrices and stationary distributions work in the context of parent-infant behavioural interactions. We start by giving a graph (right), to represent a realistic example of how “Visual Attention” behaviours might look for one mother.

The graph represents a 60 s period, in which one behaviour is coded per second. The mothers visual attention remains within three distinct behavioural states throughout the interaction: “Look at Infant”, “Look at focus object” and “Look at distraction”. She does not look anywhere else during the interaction, and her visual attention was always visible to the human coder (and therefore able to be coded). The arrows represent transitions between behaviours, and the numbers represent the number of times that transition happened. For example, we see that the mother transitioned from “Look at infant” to “Look at focus object” 7 times, from “Look at infant” to “Look at distraction” 3 times, and remained within “Look at infant” after “Look at infant” 18 times. By calculating the sum of all arrows leaving each behaviour, we can see that the mother spent 28 s in total in the “Look at infant” state (47% of the interaction), 20 s in “Look at focus object” (33% of the interaction), and 12 s in “Look at distraction” (20% of the interaction).

A Markov chain is a model describing processes of events, where the probability of each state (here, a state is a behaviour) depends only on the previous state; a transition matrix describes the Markov chain by containing the probabilities of transitioning between states. For our example, by converting the data within the above graph into probabilities, we represent the interaction in the following transition matrix *T*:

**Table:**

		**Look at infant**	**Look at focus object**	**Look at distraction**
	**Look at infant**	0.64 (18/28)	0.25 (7/28)	0.11 (3/28)
*T* =	**Look at focus object**	0.25 (5/20)	0.60 (12/20)	0.15 (3/20)
	**Look at distraction**	0.67 (8/12)	0.00 (0/12)	0.33 (4/12)

where each entry $T_{i,j}$ represents the probability of transitioning from state *i* to state *j* (the behaviours have been included for ease of comprehension). The higher the probability value (i.e., the closer to 1), the more frequently a transition between two states is likely to occur. The reverse of this is also true; the lower the probability (i.e., the closer to 0), the less frequently a transition between two states is likely to occur. Note that the probabilities of transitions from behaviour X to all other behaviours (i.e., the rows in the matrix) must sum to 1. As within this example, transition matrices in our work were calculated on a second-by-second basis. Firstly, this means that we interpret transitions as the probability of transitioning from behaviour X to behaviour Y during the next 1 s. Secondly, this also means that we include transitions from a behaviour to itself, as a person may be (and very likely will be) looking in one direction for multiple seconds (this is shown in the graph as an arrow from a behaviour to itself).

In the above example, this says that if the mother was looking at her infant at second 1, the probability that she will be looking at the infant at second 2 is 0.64. Similarly, the probability that she will transition to looking at the focus object at second 2 is 0.25, and the probability that she will transition to looking at a distraction at second 2 is 0.11. This matrix also shows us that for the given interaction, the probability that the mum will transition from looking at a distraction to looking at the focus object is 0.00; this means that this particular transition of visual attention did not occur within the period of observation. Similarly, if a transition matrix contains a probability of 1.00 between behaviour X and behaviour Y, this would mean that behaviour Y was the only behaviour that occurred directly after behaviour X (in fact, as our transitions are second-by-second, this would mean that behaviour X occurred for exactly 1 s maximum, before transitioning to behaviour Y, as longer than 1 s would be reflected in a transition from behaviour X to behaviour X).

A stationary distribution can be considered as the proportion of time spent within each behavioural state over long periods of time, and they are calculated using the data within the transition matrices. The Markov chains in our work are “ergodic”, meaning that they are both “aperiodic” (any state can be transitioned to from any other state after any number of transitions) and “irreducible” (any state can transition to any other state) (Ross, 2014). For ergodic Markov chains, the stationary distribution is a unique row vector of the form $s = \left( {s_{1} ,s_{2} ,s_{3} } \right)$ derived from the equation $s = sT$, where *T* is the transition matrix (Ross, 2014). Using our example transition matrix *T*, we can write the equation for a stationary distribution as:

$$\left( {s_{1} ,s_{2} ,s_{3} } \right) = \left( {s_{1} ,s_{2} ,s_{3} } \right)\left[ { \begin{array}{*{20}c} {0.64} & {0.25} & {0.11} \\ {0.25} & {0.60} & {0.15} \\ {0.67} & {0.00} & {0.33} \\ \end{array} } \right]$$

Therefore, we can find the corresponding stationary distribution by solving the following linear system of Eqs. (1)–(3), subject to $s_{1} + s_{2} + s_{3} = 1$ (Ross, 2014):

1 $$0.64s_{1} + 0.25s_{2} + 0.67s_{3} = s_{1}$$

2 $$0.25s_{1} + 0.60s_{2} = s_{2}$$

3 $$0.11s_{1} + 0.15s_{2} + 0.33s_{3} = s_{3}$$

By solving these equations, we find the stationary distribution:

**Table:**

	**Look at infant**	0.52
*s* =	**Look at focus object**	0.32
	**Look at distraction**	0.16

We can interpret this as the long-run proportion of time that the mother spends within each visual attention state. The higher the probability in the stationary distribution (i.e., the closer to 1), the more time spent in that specific behavioural state. The reverse is also true (i.e., low probability, close to 0, means little time is spent in that behavioural state). In the example above, over a time period of 100 s, we would expect the mother to be in the state “Look at infant” for 52 s, “Look at focus object” for 32 s, and “Look at distraction” for 16 s. Similarly, over a period of 300 s (5 min, as in our work), we would expect the mother to be in the state “Look at infant” for 156 s, “Look at focus object” for 96 s, and “Look at distraction” for 48 s. Hence, it is clear how stationary distributions can be used to infer overall duration of each behaviour.

The content of the stationary distribution follows directly from that of the transition matrix, which were calculated using behaviours extracted on a second by second basis. By extracting behaviours this way, we infer the durations of each behaviour; this information is stored inherently in the transition matrices, and is carried over when calculating the stationary distributions. As such, the stationary distribution is derived by using not only the number of occurrences of each event alone, but also by using both the duration of each event (i.e., the number of seconds spent in that state) and the subsequent events (i.e., the transitions).

Each value of the stationary distribution can be defined by $1/ \pi i$ for time step $i$ (i.e., each second), where $\pi i$ is the mean time for the parent or infant to return to behaviour $i$. This is equivalent to the probability of occurrence of that behaviour, or alternatively, the inverse of the time (i.e. number of steps) until that state recurs. Similarly, the self-transition probability values (diagonal elements in the transition matrix) are related to duration in a state on a single occasion. The average time spent in each $i$ is $1/ \pi ii$, where $\pi ii$ is the probability of staying in state $i$. It follows that the duration of each behaviour can be inferred from the probabilities within both the stationary distribution and the transition matrix.

If we then observed the following 60 s of visual attention for this mother, she might engage in new behaviours that were not present in the first 60 s (e.g., “Look at other person”), or the relative proportions that she spent within the three original states might change. Upon recalculating the transition matrix and stationary distribution, we would find the probabilities to have altered accordingly, and therefore the long-run proportion of time spent in each behavioural state would be different. For example, if a new behaviour was present, the transition matrix would expand in size to include a fourth row and column, and the stationary distribution would also include a fourth value. As we observed more and more of the interaction, we would expect both the transition matrix and the stationary distribution to become more similar to those that we would get from observing the entire interaction. However, it is likely that at some stage, the measures generated from a thin slice could be considered representative—or similar enough—to the full-session equivalents. In this case, we would need only to code the thin slice of that given length in order to calculate a representative stationary distribution.

### Frequency Tables

Here we provide comprehensive tables containing the mean behavioural frequencies (and standard deviations) by population, separated according to each thin slice (see Tables 7, 8, 9, 10, 11, 12).

**Table 7:** Cardiff mums: frequency of behaviours at each slice

Behaviour category	Slice (by length)														
	1-min					2-min				3-min			4-min		Full session
	One	Two	Three	Four	Five	OneTwo	TwoThree	ThreeFour	FourFive	OneTwo Three	TwoThree Four	ThreeFour Five	OneTwo ThreeFour	TwoThree FourFive	
Proximity	2.90 (2.19)	2.32 (1.61)	2.23 (1.72)	2.52 (2.01)	2.42 (1.81)	5.23 (3.29)	4.55 (2.65)	4.74 (3.32)	4.94 (2.50)	7.45 (4.10)	7.06 (4.18)	7.16 (3.72)	9.97 (5.37)	9.48 (4.52)	12.39 (5.72)
Body orientation	3.23 (2.72)	2.71 (2.13)	2.65 (1.86)	2.61 (1.99)	2.35 (2.13)	5.94 (4.08)	5.35 (3.04)	5.26 (3.14)	4.97 (2.91)	8.58 (4.76)	7.97 (4.39)	7.61 (3.76)	11.19 (5.84)	10.32 (5.03)	13.55 (6.39)
Visual attention	16.13 (11.84)	15.77 (12.12)	13.71 (9.68)	12.87 (9.94)	11.81 (9.09)	31.90 (21.47)	29.48 (20.70)	26.58 (18.70)	24.68 (17.60)	45.61 (29.50)	42.35 (29.60)	38.39 (25.85)	58.48 (37.77)	54.16 (36.15)	70.29 (43.76)
Posture	2.32 (2.81)	1.65 (1.18)	1.52 (1.24)	1.65 (1.49)	1.74 (1.52)	3.97 (3.58)	3.16 (1.92)	3.16 (2.27)	3.39 (2.32)	5.48 (3.94)	4.81 (2.67)	4.90 (3.02)	7.13 (4.20)	6.55 (3.29)	8.87 (4.74)
Touch right hand	14.68 (9.22)	14.77 (8.44)	13.71 (8.1)	14.65 (7.32)	9.94 (6.51)	29.45 (15.63)	28.48 (14.42)	28.35 (14.12)	24.58 (11.65)	43.16 (21.60)	43.13 (20.03)	38.29 (19.01)	57.81 (27.28)	53.06 (24.42)	67.74 (31.31)
Touch left hand	5.26 (2.66)	3.81 (2.12)	3.19 (1.89)	3.23 (1.81)	2.06 (1.41)	9.06 (3.78)	7.00 (3.48)	6.42 (2.76)	5.29 (2.30)	12.26 (4.72)	10.23 (4.15)	8.48 (3.12)	15.48 (5.23)	12.29 (4.22)	17.55 (5.28)
Physical play	10.90 (9.77)	9.39 (7.96)	10.06 (8.11)	9.55 (7.30)	9.13 (6.56)	20.29 (16.32)	19.45 (14.41)	19.61 (14.48)	18.68 (12.50)	30.35 (22.38)	29.00 (20.03)	28.74 (19.03)	39.90 (27.29)	38.13 (24.22)	49.03 (31.04)
Vocalisation	23.74 (8.29)	23.13 (8.93)	20.19 (7.16)	19.68 (8.77)	18.52 (8.07)	46.87 (15.76)	43.32 (13.92)	39.87 (14.20)	38.19 (15.38)	67.06 (21.04)	63.00 (20.67)	58.39 (20.71)	86.74 (27.69)	81.52 (27.05)	105.26 (33.85)

Mean frequency of behaviours within a category for each thin slice for the Cardiff Mums (averaged across individual caregivers); standard deviations are provided in brackets

**Table 8:** Cardiff infants: frequency of behaviours at each slice

Behaviour category	Slice (by length)														
	1-min					2-min				3-min			4-min		Full session
	One	Two	Three	Four	Five	OneTwo	TwoThree	ThreeFour	FourFive	OneTwo Three	TwoThree Four	ThreeFour Five	OneTwo ThreeFour	TwoThree FourFive	
Body orientation	4.90 (2.57)	4.48 (2.18)	4.48 (2.73)	4.81 (3.20)	4.71 (3.34)	9.39 (3.71)	8.97 (4.06)	9.29 (5.06)	9.52 (5.39)	13.87 (5.21)	13.77 (6.38)	14.00 (7.47)	18.68 (7.39)	18.48 (8.64)	23.39 (9.83)
Visual attention	12.19 (8.86)	13.97 (9.75)	13.16 (8.86)	13.23 (8.13)	11.74 (8.14)	26.16 (17.34)	27.13 (16.99)	26.39 (15.86)	24.97 (14.32)	39.32 (23.63)	40.35 (23.11)	38.13 (21.5)	52.55 (29.19)	52.10 (28.59)	64.29 (34.46)
Posture	4.65 (4.92)	4.35 (4.27)	4.58 (5.18)	5.23 (4.98)	6.03 (5.29)	9.00 (8.24)	8.94 (8.65)	9.81 (8.88)	11.26 (8.55)	13.58 (12.59)	14.16 (11.97)	15.84 (12.34)	18.81 (15.83)	20.19 (15.29)	24.84 (19.10)
Touch right hand	10.48 (6.72)	9.65 (6.58)	10.58 (7.06)	10.84 (6.94)	10.90 (6.64)	20.13 (10.84)	20.23 (12.15)	21.42 (13.23)	21.74 (11.68)	30.71 (16.05)	31.06 (17.84)	32.32 (17.46)	41.55 (21.4)	41.97 (22.18)	52.45 (25.73)
Touch left hand	9.45 (6.55)	11.26 (7.20)	10.90 (6.83)	12.26 (7.77)	8.90 (6.35)	20.71 (12.29)	22.16 (12.57)	23.16 (13.59)	21.16 (12.99)	31.61 (16.82)	34.42 (19.00)	32.06 (18.53)	43.87 (22.75)	43.32 (23.62)	52.77 (27.33)
Physical play	4.42 (2.25)	5.16 (3.09)	4.77 (2.46)	3.65 (1.79)	3.03 (2.12)	9.58 (4.29)	9.94 (4.99)	8.42 (3.44)	6.68 (3.24)	14.35 (5.77)	13.58 (5.90)	11.45 (4.42)	18.00 (6.58)	16.61 (6.64)	21.03 (7.34)
Vocalisation	7.13 (7.27)	7.39 (7.52)	7.42 (5.67)	8.29 (5.95)	6.39 (4.72)	14.52 (12.97)	14.81 (11.97)	15.71 (10.18)	14.68 (9.31)	21.94 (17.28)	23.10 (16.33)	22.10 (13.01)	30.23 (21.39)	29.48 (18.66)	36.61 (23.59)

Mean frequency of behaviours within a category for each thin slice for the Cardiff Infants (averaged across individual infants); standard deviations are provided in brackets

**Table 9:** Bristol mums: frequency of behaviours at each slice

Behaviour category	Slice (by length)														
	1-min					2-min				3-min			4-min		Full session
	One	Two	Three	Four	Five	OneTwo	TwoThree	ThreeFour	FourFive	OneTwo Three	TwoThree Four	ThreeFour Five	OneTwo ThreeFour	TwoThree FourFive	
Proximity	1.87 (1.71)	1.67 (1.25)	1.73 (1.84)	1.87 (1.75)	1.47 (1.41)	3.53 (2.60)	3.40 (3.03)	3.60 (3.30)	3.33 (2.72)	5.27 (4.14)	5.27 (4.48)	5.07 (4.46)	7.13 (5.50)	6.73 (5.65)	8.60 (6.72)
Body orientation	1.80 (1.97)	1.47 (1.59)	1.07 (1.00)	1.93 (2.57)	1.53 (2.63)	3.27 (3.34)	2.53 (2.55)	3.00 (3.44)	3.47 (4.81)	4.33 (4.25)	4.47 (4.88)	4.53 (5.74)	6.27 (6.68)	6.00 (7.17)	7.80 (8.96)
Head orientation	3.20 (2.90)	3.47 (3.98)	3.87 (4.59)	3.87 (3.40)	2.53 (3.40)	6.67 (6.34)	7.33 (8.36)	7.73 (7.30)	6.40 (6.09)	10.53 (10.73)	11.20 (11.05)	10.27 (10.19)	14.40 (13.39)	13.73 (13.77)	16.93 (16.15)
Visual attention	6.40 (6.56)	5.47 (5.35)	6.13 (8.75)	6.00 (6.89)	6.00 (7.84)	11.87 (11.15)	11.60 (12.39)	12.13 (15.05)	12.00 (14.42)	18.00 (16.09)	17.60 (18.17)	18.13 (22.43)	24.00 (21.30)	23.60 (25.21)	30.00 (27.9)
Facial expression	7.67 (8.47)	8.93 (10.95)	10.67 (13.40)	8.80 (9.82)	7.20 (8.58)	16.60 (19.19)	19.60 (23.94)	19.47 (22.47)	16.00 (17.61)	27.27 (32.13)	28.40 (32.39)	26.67 (29.84)	36.07 (40.39)	35.60 (39.24)	43.27 (46.96)
Posture	1.27 (1.88)	0.93 (0.77)	0.73 (0.44)	0.87 (0.72)	0.73 (0.44)	2.20 (2.17)	1.67 (1.14)	1.60 (1.08)	1.60 (1.08)	2.93 (2.46)	2.53 (1.82)	2.33 (1.49)	3.80 (2.95)	3.27 (2.21)	4.53 (3.30)
Touch right hand	7.20 (8.57)	5.87 (5.18)	5.40 (6.04)	6.33 (7.86)	5.13 (5.46)	13.07 (13.51)	11.27 (10.18)	11.73 (13.30)	11.47 (13.08)	18.47 (17.82)	17.60 (17.58)	16.87 (18.54)	24.80 (25.13)	22.73 (22.73)	29.93 (30.02)
Touch left hand	4.93 (5.62)	4.00 (4.13)	4.07 (4.11)	4.20 (4.87)	2.87 (3.56)	8.93 (8.87)	8.07 (7.87)	8.27 (8.49)	7.07 (7.34)	13.00 (12.62)	12.27 (11.95)	11.13 (11.21)	17.20 (17.12)	15.13 (14.97)	20.07 (19.87)
Hand movements	1.60 (1.93)	1.00 (1.15)	1.33 (2.62)	1.53 (3.36)	1.13 (1.41)	2.60 (2.96)	2.33 (3.75)	2.87 (5.99)	2.67 (4.36)	3.93 (5.40)	3.87 (7.11)	4.00 (6.94)	5.47 (8.68)	5.00 (8.06)	6.60 (9.65)
Physical play	1.13 (2.16)	1.07 (1.91)	1.53 (2.60)	0.93 (1.12)	1.33 (2.09)	2.20 (4.07)	2.60 (4.32)	2.47 (3.67)	2.27 (3.19)	3.73 (6.38)	3.53 (5.29)	3.80 (5.74)	4.67 (7.28)	4.87 (7.27)	6.00 (9.17)
Vocalisation	17.80 (10.42)	18.00 (10.30)	19.60 (11.3)	15.87 (11.04)	12.73 (9.02)	35.80 (20.09)	37.60 (20.22)	35.47 (21.22)	28.60 (18.42)	55.40 (29.86)	53.47 (29.79)	48.20 (28.93)	71.27 (38.62)	66.20 (37.6)	84.00 (46.38)

Mean frequency of behaviours within a category for each thin slice for the Bristol Mums (averaged across individual caregivers); standard deviations are provided in brackets

**Table 10:** Bristol infants (1): frequency of behaviours at each slice

Behaviour category	Slice (by length)														
	1-min					2-min				3-min			4-min		Full session
	One	Two	Three	Four	Five	OneTwo	TwoThree	ThreeFour	FourFive	OneTwo Three	TwoThree Four	ThreeFour Five	OneTwo ThreeFour	TwoThree FourFive	
Body orientation	1.60 (1.58)	1.33 (1.53)	1.27 (2.38)	1.93 (3.62)	1.27 (1.73)	2.93 (2.98)	2.60 (3.61)	3.20 (5.88)	3.20 (5.32)	4.20 (4.94)	4.53 (6.97)	4.47 (7.58)	6.13 (8.29)	5.80 (8.66)	7.40 (9.95)
Head orientation	4.40 (4.36)	4.40 (5.81)	3.93 (5.56)	3.53 (4.15)	4.13 (4.83)	8.80 (9.67)	8.33 (10.43)	7.47 (9.22)	7.67 (8.65)	12.73 (14.52)	11.87 (13.96)	11.60 (13.79)	16.27 (17.97)	16.00 (18.28)	20.40 (22.21)
Visual attention	7.67 (6.01)	7.67 (6.53)	7.80 (7.90)	8.13 (8.64)	8.13 (8.70)	15.33 (11.56)	15.47 (13.65)	15.93 (16.18)	16.27 (16.44)	23.13 (18.30)	23.60 (21.26)	24.07 (23.69)	31.27 (25.67)	31.73 (28.58)	39.40 (33.01)
Facial expression	8.67 (5.50)	6.93 (9.30)	8.47 (8.68)	7.87 (7.59)	6.67 (9.40)	15.60 (13.67)	15.40 (15.83)	16.33 (15.80)	14.53 (15.86)	24.07 (20.61)	23.27 (22.22)	23.00 (23.22)	31.93 (26.87)	29.93 (27.77)	38.60 (32.08)
Posture	0.93 (0.77)	0.87 (0.72)	0.87 (0.81)	0.87 (0.96)	0.67 (0.47)	1.80 (1.38)	1.73 (1.39)	1.73 (1.61)	1.53 (1.31)	2.67 (1.99)	2.60 (2.27)	2.40 (1.99)	3.53 (2.85)	3.27 (2.64)	4.20 (3.23)
Touch right hand	4.93 (3.92)	3.07 (2.79)	4.13 (4.30)	3.20 (3.04)	2.80 (4.23)	8.00 (6.26)	7.20 (6.39)	7.33 (6.80)	6.00 (6.78)	12.13 (9.53)	10.40 (8.89)	10.13 (10.05)	15.33 (11.78)	13.20 (12.01)	18.13 (14.43)
Touch left hand	3.93 (5.50)	1.47 (1.50)	2.13 (3.48)	3.13 (5.38)	2.80 (4.48)	5.40 (6.65)	3.60 (4.84)	5.27 (8.54)	5.93 (9.47)	7.53 (9.67)	6.73 (9.94)	8.07 (12.79)	10.67 (14.49)	9.53 (14.20)	13.47 (18.61)
Hand movements	3.07 (4.52)	1.47 (2.85)	2.40 (3.90)	2.40 (3.40)	2.27 (3.26)	4.53 (6.58)	3.87 (6.33)	4.80 (7.16)	4.67 (6.64)	6.93 (9.62)	6.27 (9.40)	7.07 (10.40)	9.33 (12.35)	8.53 (12.55)	11.60 (15.30)
Physical play	1.60 (2.36)	1.33 (1.92)	1.27 (1.73)	1.53 (2.55)	1.27 (1.73)	2.93 (4.11)	2.60 (3.38)	2.80 (4.25)	2.80 (4.17)	4.20 (5.28)	4.13 (5.90)	4.07 (5.89)	5.73 (7.58)	5.40 (7.44)	7.00 (8.97)
Vocalisation	5.07 (5.85)	9.87 (8.14)	8.73 (9.39)	11.53 (10.63)	10.20 (9.14)	14.93 (13.37)	18.60 (17.16)	20.27 (19.40)	21.73 (18.78)	23.67 (22.23)	30.13 (27.08)	30.47 (27.57)	35.20 (32.15)	40.33 (35.36)	45.40 (40.29)

Mean frequency of behaviours within a category for each thin slice for the Bristol Infants (1) (averaged across individual infants); standard deviations are provided in brackets

**Table 11:** Bristol dads: frequency of behaviours at each slice

Behaviour category	Slice (by length)														
	1-min					2-min				3-min			4-min		Full session
	One	Two	Three	Four	Five	OneTwo	TwoThree	ThreeFour	FourFive	OneTwo Three	TwoThree Four	ThreeFour Five	OneTwo ThreeFour	TwoThree FourFive	
Proximity	2.24 (1.66)	2.12 (2.30)	2.18 (1.92)	1.59 (1.09)	1.82 (1.69)	4.35 (3.55)	4.29 (3.58)	3.76 (2.62)	3.41 (2.59)	6.53 (4.82)	5.88 (4.51)	5.59 (3.77)	8.12 (5.65)	7.71 (5.85)	9.94 (6.94)
Body orientation	2.82 (2.46)	2.65 (3.38)	1.53 (0.98)	1.47 (1.19)	1.29 (1.07)	5.47 (5.37)	4.18 (4.03)	3.00 (2.00)	2.76 (1.63)	7.00 (6.00)	5.65 (5.14)	4.29 (2.29)	8.47 (7.06)	6.94 (5.55)	9.76 (7.51)
Head orientation	7.59 (4.78)	6.29 (4.98)	5.53 (4.29)	5.06 (3.92)	4.65 (4.52)	13.88 (8.89)	11.82 (8.13)	10.59 (7.75)	9.71 (7.46)	19.41 (12.05)	16.88 (11.51)	15.24 (11.35)	24.47 (15.59)	21.53 (15.22)	29.12 (19.26)
Visual attention	9.65 (6.71)	7.24 (6.82)	7.41 (6.04)	6.76 (5.34)	6.71 (5.36)	16.88 (12.24)	14.65 (12.31)	14.18 (10.99)	13.47 (9.97)	24.29 (17.59)	21.41 (17.23)	20.88 (15.55)	31.06 (22.57)	28.12 (21.59)	37.76 (26.94)
Facial expression	7.18 (4.48)	5.41 (5.15)	6.12 (5.94)	5.35 (5.01)	5.53 (6.18)	12.59 (8.83)	11.53 (10.71)	11.47 (10.39)	10.88 (10.76)	18.71 (14.27)	16.88 (15.01)	17.00 (16.14)	24.06 (18.69)	22.41 (20.70)	29.59 (24.17)
Posture	2.06 (1.55)	1.29 (1.18)	1.41 (1.14)	1.00 (0.00)	1.18 (0.71)	3.35 (1.94)	2.71 (1.56)	2.41 (1.14)	2.18 (0.71)	4.76 (2.31)	3.71 (1.56)	3.59 (1.09)	5.76 (2.31)	4.88 (1.84)	6.94 (2.67)
Touch right hand	6.88 (4.75)	5.53 (4.38)	4.71 (2.89)	4.00 (3.46)	4.65 (3.51)	12.41 (8.81)	10.24 (6.80)	8.71 (6.05)	8.65 (6.33)	17.12 (11.31)	14.24 (9.75)	13.35 (8.70)	21.12 (14.2)	18.88 (12.45)	25.76 (16.96)
Touch left hand	6.59 (3.71)	4.00 (4.39)	4.29 (3.97)	3.71 (3.19)	4.00 (3.91)	10.59 (7.40)	8.29 (8.09)	8.00 (6.56)	7.71 (6.29)	14.88 (11.08)	12.00 (10.55)	12.00 (9.96)	18.59 (13.70)	16.00 (13.95)	22.59 (17.01)
Hand movements	7.88 (5.39)	5.94 (5.13)	5.18 (4.13)	5.24 (4.98)	4.35 (3.34)	13.82 (10.02)	11.12 (8.97)	10.41 (8.83)	9.59 (7.95)	19.00 (13.94)	16.35 (13.43)	14.76 (11.79)	24.24 (18.49)	20.71 (16.37)	28.59 (21.52)
Physical play	2.00 (1.75)	1.71 (1.13)	1.65 (1.49)	1.53 (1.09)	1.53 (1.54)	3.71 (2.76)	3.35 (2.42)	3.18 (2.50)	3.06 (2.41)	5.35 (4.12)	4.88 (3.39)	4.71 (3.71)	6.88 (5.10)	6.41 (4.64)	8.41 (6.35)
Vocalisation	16.88 (6.37)	16.35 (7.98)	16.00 (7.33)	12.00 (6.44)	11.41 (7.16)	33.24 (13.33)	32.35 (14.03)	28.00 (12.76)	23.41 (11.97)	49.24 (19.18)	44.35 (18.84)	39.41 (18.25)	61.24 (23.55)	55.76 (24.40)	72.65 (28.86)

Mean frequency of behaviours within a category for each thin slice for the Bristol Dads (averaged across individual caregivers); standard deviations are provided in brackets

**Table 12:** Bristol infants (2): frequency of behaviours at each slice

Behaviour category	Slice (by length)														
	1-min					2-min				3-min			4-min		Full session
	One	Two	Three	Four	Five	OneTwo	TwoThree	ThreeFour	FourFive	OneTwo Three	TwoThree Four	ThreeFour Five	OneTwo ThreeFour	TwoThree FourFive	
Body orientation	1.65 (1.23)	1.06 (0.64)	1.18 (0.92)	1.00 (0.49)	1.00 (0.59)	2.71 (1.52)	2.24 (1.48)	2.18 (1.29)	2.00 (0.97)	3.88 (2.08)	3.24 (1.86)	3.18 (1.62)	4.88 (2.45)	4.24 (2.16)	5.88 (2.81)
Head orientation	7.18 (5.73)	6.18 (4.99)	7.06 (6.17)	5.88 (4.80)	4.76 (3.83)	13.35 (9.63)	13.24 (10.45)	12.94 (10.16)	10.65 (8.22)	20.41 (14.62)	19.12 (14.54)	17.71 (13.43)	26.29 (18.62)	23.88 (17.81)	31.06 (22.06)
Visual attention	8.65 (6.16)	7.59 (6.00)	9.18 (8.35)	8.18 (6.53)	6.29 (4.91)	16.24 (11.79)	16.76 (14.11)	17.35 (13.96)	14.47 (10.83)	25.41 (19.53)	24.94 (19.69)	23.65 (18.45)	33.59 (25.16)	31.24 (24.20)	39.88 (29.72)
Facial expression	5.59 (5.37)	5.29 (4.16)	5.53 (6.33)	5.94 (6.49)	4.24 (4.44)	10.88 (9.30)	10.82 (10.18)	11.47 (12.54)	10.18 (10.27)	16.41 (15.19)	16.76 (16.40)	15.71 (16.09)	22.35 (21.34)	21.00 (19.94)	26.59 (24.95)
Posture	1.41 (1.42)	1.06 (1.06)	0.94 (0.64)	0.94 (0.64)	0.82 (0.51)	2.47 (1.85)	2.00 (1.37)	1.88 (1.28)	1.76 (1.00)	3.41 (2.20)	2.94 (1.86)	2.71 (1.60)	4.35 (2.66)	3.76 (2.18)	5.18 (2.98)
Touch right hand	3.18 (3.54)	6.12 (5.99)	4.94 (4.57)	5.94 (6.72)	3.53 (4.17)	9.29 (9.05)	11.06 (10.48)	10.88 (11.05)	9.47 (10.69)	14.24 (13.41)	17.00 (16.84)	14.41 (15.04)	20.18 (19.73)	20.53 (20.85)	23.71 (23.75)
Touch left hand	4.24 (3.95)	4.00 (5.28)	2.94 (4.15)	3.71 (6.37)	2.71 (5.06)	8.24 (8.50)	6.94 (9.21)	6.65 (10.03)	6.41 (10.56)	11.18 (12.26)	10.65 (15.10)	9.35 (14.40)	14.88 (17.72)	13.35 (19.39)	17.59 (21.97)
Hand movements	4.65 (4.23)	5.53 (5.26)	4.65 (4.42)	6.00 (6.13)	3.65 (4.39)	10.18 (8.65)	10.18 (9.21)	10.65 (10.02)	9.65 (10.21)	14.82 (12.47)	16.18 (14.88)	14.29 (14.03)	20.82 (18.17)	19.82 (18.64)	24.47 (21.99)
Physical play	2.53 (3.15)	1.76 (1.70)	1.94 (2.31)	1.82 (1.72)	1.88 (1.84)	4.29 (4.72)	3.71 (3.98)	3.76 (3.98)	3.71 (3.51)	6.24 (6.96)	5.53 (5.67)	5.65 (5.74)	8.06 (8.63)	7.41 (7.42)	9.94 (10.41)
Vocalisation	6.12 (4.07)	7.06 (6.58)	6.88 (5.02)	9.18 (8.24)	6.29 (7.74)	13.18 (9.50)	13.94 (10.29)	16.06 (11.69)	15.47 (15.62)	20.06 (13.09)	23.12 (17.69)	22.35 (18.60)	29.24 (20.4)	29.41 (24.66)	35.53 (27.18)

Mean frequency of behaviours within a category for each thin slice for the Bristol Infants (2) (averaged across individual infants); standard deviations are provided in brackets

### Transition Matrices

Here we provide two sets of analyses related to transition matrices. “Absolute Differences Plot” section includes a plot to show the absolute differences between transition matrices calculated at each thin slice and full sessions; “Chi-Squared Tables” section provides comprehensive tables containing the results of chi-squared tests on the absolute differences between thin slice and full session transition matrices.

#### Absolute Differences Plot

See Fig. 2.

#### Chi Squared Tables

See Tables 13, 14, 15, 16, 17 and 18.

**Table 13:** Cardiff mums: chi-squared test on absolute differences between thin slice and full session transition matrices

Behaviour (by category)	Slice (by length)													
	1-min					2-min				3-min			4-min	
	One	Two	Three	Four	Five	OneTwo	TwoThree	ThreeFour	FourFive	OneTwo Three	TwoThree Four	ThreeFour Five	OneTwo ThreeFour	TwoThree FourFive
Out of reach	42.38***	34.36***	29.00***	47.06***	32.03***	17.98***	11.81**	19.93***	20.69***	**4.57**	**6.07**	**6.08**	**2.01**	**0.64**
Within reach	138.37***	138.07***	139.57***	133.88***	141.10***	78.31***	82.87***	72.61***	73.67***	35.88***	35.09***	30.71***	**7.85***	**9.91***
Loom	**0.60**	**0.34**	**1.38**	**2.72**	**3.70**	**0.54**	**0.15**	**0.97**	**3.14**	**1.68**	**0.10**	**1.96**	**0.31**	**0.42**
NPTC caregiver proximity	**0.52**	**6.89**	**6.89**	**3.00**	**6.89**	**0.52**	**6.89**	**3.00**	**3.00**	**0.52**	**3.00**	**3.00**	**0.00**	**3.00**
BO to infant	160.22***	162.28***	165.60***	154.44***	161.46***	92.61***	96.73***	88.76***	85.76***	43.79***	40.81***	39.16***	**10.08***	**10.44***
BO to other person/object	23.02***	14.41**	10.64*	21.30***	12.79**	12.04**	3.67	8.78*	11.01*	**1.77**	**2.25**	**4.27**	**0.96**	**0.50**
BO to object (focus of the activity)	7.49	5.28	4.48	18.84***	1.51	**2.35**	**1.73**	**9.46***	**7.84***	**0.41**	**3.81**	**3.66**	**0.63**	**1.04**
NPTC caregiver body orientation	**0.99**	**2.98**	**1.49**	**1.50**	**8.58***	**0.78**	**0.64**	**0.26**	**1.50**	**0.36**	**0.71**	**0.26**	**0.00**	**0.71**
Look at infant	6.56	7.63	4.26	10.12	13.80	1.01	2.32	2.21	1.21	**0.98**	**1.12**	**0.86**	**0.46**	**0.15**
Look at focus object	31.03***	35.10***	34.90***	30.66***	37.12***	19.96**	20.46**	14.99*	21.10**	**8.59**	**7.26**	**9.02**	**1.22**	**3.14**
Look at other object	9.56	7.96	7.64	6.51	6.88	**4.94**	**3.48**	**3.76**	**3.20**	**1.77**	**1.53**	**1.70**	**0.62**	**0.19**
Look at object outside of view	18.59**	21.71**	20.69**	16.56*	21.03**	**12.36**	**14.09***	**9.20**	**9.81**	**6.29**	**4.92**	**3.18**	**1.05**	**1.24**
Look at other person	**1.75**	**1.75**	**5.75**	**1.75**	**0.75**	**1.75**	**5.75**	**0.75**	**0.64**	**5.75**	**0.75**	**0.00**	**0.75**	**0.00**
No visual attention	**4.86**	**5.14**	**3.46**	**5.85**	**5.25**	**2.53**	**1.96**	**2.09**	**3.10**	**0.83**	**0.90**	**0.82**	**0.16**	**0.29**
NPTC visual attention	76.01***	66.08***	63.84***	74.35***	63.8***	42.59***	33.53***	41.53***	40.66***	**18.19***	**16.75***	**18.40***	**4.84**	**3.77**
Lie on side	62.84***	25.54**	21.70**	7.62	16.50*	**14.39**	**11.58**	**2.18**	**0.96**	**6.57**	**1.46**	**0.53**	**0.14**	**0.40**
Sit on floor	112.8***	113.98***	117.64***	115.81***	113.86***	55.85***	64.97***	63.35***	54.86***	31.86***	30.09***	22.34**	**9.14**	**5.63**
Sit on object	**0.00**	**8.00**	**8.00**	**8.00**	**8.00**	**0.00**	**8.00**	**8.00**	**8.00**	**0.00**	**8.00**	**8.00**	**0.00**	**8.00**
Stand up	**0.91**	**1.91**	**2.38**	**1.76**	**1.76**	**0.52**	**0.61**	**0.99**	**0.71**	**0.71**	**0.52**	**0.75**	**0.20**	**0.27**
Crawl	**5.64**	**0.22**	**1.76**	**0.35**	**1.63**	**0.22**	**0.31**	**0.20**	**0.01**	**0.31**	**0.23**	**0.04**	**0.23**	**0.00**
Crouched down	47.22***	33.32***	49.02***	41.29***	48.49***	16.50*	12.27	22.76**	28.19***	**6.40**	**3.65**	**10.45**	**1.81**	**0.73**
Walk	**1.49**	**1.48**	**3.17**	**10.82**	**6.50**	**0.54**	**2.01**	**2.16**	**10.82**	**0.84**	**0.56**	**2.16**	**0.00**	**0.56**
Dance	**3.00**	**3.00**	**3.00**	**3.00**	**0.00**	**3.00**	**3.00**	**3.00**	**0.00**	**3.00**	**3.00**	**0.00**	**3.00**	**0.00**
NPTC caregiver posture/action	**0.64**	**1.54**	**12.25**	**12.25**	**12.25**	**0.00**	**1.54**	**12.25**	**12.25**	**0.00**	**1.54**	**12.25**	**0.00**	**1.54**
Caregiver touch R	78.54***	83.41***	78.47***	77.84***	80.80***	48.18***	46.91***	42.77***	43.38***	21.70***	19.78***	18.57***	**5.12**	**4.88**
No caregiver touch R	59.82***	51.66***	51.93***	54.83***	52.12***	32.04***	27.91***	30.26***	30.31***	14.10**	11.76*	12.97*	**3.67**	**2.39**
Not possible to code touch R	50.06***	52.13***	51.79***	54.74***	52.19***	28.26***	31.47***	31.68***	31.00***	12.48*	14.92**	13.30**	**3.45**	**3.80**
Caregiver touch L	60.68***	52.79***	59.49***	55.50***	54.53***	33.34***	32.46***	33.37***	28.06***	17.54**	14.64**	14.09**	**4.75**	**2.79**
No caregiver touch L	60.93***	72.54***	66.95***	72.56***	68.9***	36.01***	38.76***	39.92***	41.29***	13.84**	20.27***	17.23**	**4.73**	**5.45**
Not possible to code touch L	50.36***	43.70***	41.60***	47.51***	53.11***	27.22***	17.68**	21.02***	28.01***	10.71*	7.65	10.71*	**2.01**	**2.13**
Physical play evident	45.83***	52.06***	46.20***	48.42***	43.46***	27.88***	27.45***	25.98***	30.67***	11.28**	12.14**	12.25**	**2.35**	**3.77**
No physical play	144.07***	132.27***	137.24***	136.46***	129.53***	80.45***	76.96***	78.38***	72.51***	37.71***	33.83***	32.42***	10.14**	7.13*
Not possible to code physical play	0.21	2.86	0.82	2.19	9.89**	**0.14**	**1.48**	**1.22**	**2.19**	**0.13**	**1.05**	**1.22**	**0.00**	**1.05**
Speech	54.30***	56.33***	53.26***	56.54***	62.54***	30.19***	28.89***	29.45***	36.60***	**11.24**	**12.30**	**15.65***	**2.18**	**4.15**
Musical Sounds	**1.75**	**1.08**	**0.12**	**1.75**	**1.75**	**1.08**	**0.00**	**0.12**	**1.75**	**0.00**	**0.00**	**0.12**	**0.00**	**0.00**
Laugh	**4.25**	**3.14**	**2.95**	**3.37**	**2.24**	**2.67**	**1.72**	**1.87**	**1.31**	**1.07**	**0.84**	**0.59**	**0.43**	**0.12**
Nervous laugh	**3.00**	**0.50**	**1.50**	**0.50**	**0.38**	**0.50**	**0.75**	**0.00**	**0.00**	**0.75**	**0.12**	**0.12**	**0.12**	**0.00**
Vocal Imitation	**0.91**	**1.36**	**1.26**	**0.83**	**1.50**	**0.65**	**0.65**	**0.63**	**0.61**	**0.23**	**0.24**	**0.40**	**0.09**	**0.05**
Bodily sounds	**2.50**	**0.93**	**1.88**	**0.86**	**1.09**	**0.93**	**0.21**	**0.82**	**0.86**	**0.21**	**0.19**	**0.56**	**0.19**	**0.00**
Scream	**0.67**	**2.00**	**0.67**	**0.67**	**2.00**	**0.67**	**0.67**	**0.17**	**0.67**	**0.67**	**0.17**	**0.17**	**0.00**	**0.17**
Non-verbal sound	**6.24**	**5.29**	**7.61**	**6.34**	**6.25**	**2.84**	**4.06**	**4.91**	**3.68**	**1.47**	**2.09**	**2.63**	**0.47**	**0.61**
Silent	123.97***	123.83***	126.08***	121.89***	116.93***	71.21***	72.78***	70.90***	64.49***	34.19***	32.73***	29.31***	**9.29**	**7.36**

**p* < 0.05; ***p* < 0.01; ****p* < 0.001. NPTC, not possible to code; BO, body orientation. From top to bottom, behavioural groups shown are: Proximity, Body Orientation, Visual Attention, Posture, Touch R, Touch L, Physical Play, and Vocalisation. Only behaviours that occurred within this population are included. Behaviours have been arranged according to the corresponding behavioural groups. The bold values highlight an example criterion which could be used to identify an appropriate length of time to code for each behaviour, if wanting to investigate transitions between behaviours: i.e. where all chi-squared values for a given slice length have *p* < 0.05. Fully-boldened regions represent the corresponding “cut off”, where *p* < 0.05 for all slices to the right of the line; indicating that the slice length rightward adjacent to the black line would be an appropriate coding timeframe for the given behavioural group. E.g., data within the first rows suggest that coding 4-min of Proximity would be suitable

**Table 14:** Cardiff infants: chi-squared test on absolute differences between thin slice and full session transition matrices

Behaviour (by category)	Slice (by length)													
	1-min					2-min				3-min			4-min	
	One	Two	Three	Four	Five	OneTwo	TwoThree	ThreeFour	FourFive	OneTwo Three	TwoThree Four	ThreeFour Five	OneTwo ThreeFour	TwoThree FourFive
BO to caregiver 1	51.00***	55.70***	51.33***	44.43***	52.86***	31.38***	31.88***	20.34***	23.74***	15.54**	9.88*	9.32*	**1.91**	**3.49**
BO to different person/object	19.73***	22.23***	25.28***	25.41***	15.23**	12.58**	14.69**	17.75***	9.74*	**8.04***	**8.51***	**5.37**	**3.50**	**0.90**
BO to object (focus of activity)	83.49***	87.84***	89.32***	83.31***	90.01***	45.30***	51.48***	46.75***	52.61***	21.36***	23.20***	25.52***	**5.13**	**6.54**
NPTC infant body orientation	**15.90****	**7.73**	**3.15**	**12.98****	**14.42****	**7.88***	**0.35**	**1.70**	**9.26***	**1.15**	**0.13**	**1.28**	**0.13**	**0.16**
Look at caregiver 1	**4.33**	**3.53**	**6.26**	**4.01**	**3.28**	**1.66**	**2.97**	**3.00**	**1.52**	**1.67**	**1.16**	**0.87**	**0.55**	**0.12**
Look at focus object	59.99***	60.25***	59.32***	49.95***	67.63***	35.20***	32.53***	25.52***	32.57***	**14.75***	**10.88**	**14.24***	**1.78**	**3.62**
Look at other object	**5.48**	**4.85**	**9.01**	**8.10**	**4.79**	**1.84**	**5.32**	**5.54**	**3.24**	**2.49**	**3.12**	**1.57**	**1.00**	**0.51**
Look at object outside of view	24.54***	22.79**	24.40***	23.39**	24.85***	**13.34**	**12.09**	**12.64**	**12.98**	**5.49**	**4.65**	**6.03**	**1.13**	**1.51**
Look at other person	**1.63**	**1.86**	**1.19**	**3.71**	**0.81**	**0.61**	**1.15**	**1.19**	**0.81**	**0.13**	**1.15**	**0.36**	**0.13**	**0.32**
No visual attention	**4.27**	**3.77**	**4.14**	**3.64**	**5.16**	**2.40**	**1.99**	**2.07**	**2.68**	**1.14**	**0.70**	**1.07**	**0.23**	**0.12**
NPTC visual attention	66.66***	76.00***	74.65***	83.62***	65.22***	37.73***	42.11***	48.79***	44.27***	15.31*	24.73***	19.64**	**6.22**	**6.26**
Lie down	**13.26**	**7.09**	**4.21**	**5.53**	**4.32**	**7.14**	**16.51**	**3.74**	**3.95**	**17.96**	**0.17**	**3.04**	**0.11**	**0.03**
Lie on one side	**1.06**	**7.62**	**10.94**	**18.00**	**18.00**	**0.74**	**4.76**	**10.94**	**18.00**	**0.00**	**4.76**	**10.94**	**0.00**	**4.76**
Sit on the floor	109.54***	114.18***	114.51***	112.99***	115.53***	65.53***	67.08***	64.65***	65.73***	27.69**	30.42***	28.88**	**6.64**	**8.71**
Sit on an object	1.50	0.50	3.00	3.00	3.00	**0.00**	**0.50**	**3.00**	**3.00**	**0.00**	**0.50**	**3.00**	**0.00**	**0.50**
Standing	42.46***	41.33***	32.02***	30.11***	34.95***	**20.79***	**18.93***	**16.16**	**16.75**	**7.82**	**9.38**	**8.44**	**1.78**	**3.82**
Crawling	**12.38**	**9.10**	**4.21**	**8.04**	**10.09**	**7.21**	**1.93**	**2.74**	**6.06**	**1.16**	**0.46**	**2.16**	**0.03**	**0.25**
Walking	**11.91**	**7.77**	**7.59**	**5.13**	**7.45**	**6.65**	**4.42**	**1.76**	**2.78**	**1.70**	**1.18**	**0.79**	**0.24**	**0.32**
Running	**4.00**	**2.67**	**0.67**	**4.00**	**2.00**	**2.67**	**2.00**	**0.67**	**2.00**	**2.00**	**0.50**	**0.17**	**0.50**	**0.00**
Held/in hold	24.76**	8.72	23.16*	13.10	20.78*	**10.01**	**1.91**	**13.54**	**5.74**	**3.05**	**0.46**	**6.59**	**0.84**	**0.26**
Try to move in another way	**5.80**	**6.51**	**8.83**	**5.66**	**10.56**	**3.18**	**4.21**	**4.72**	**3.92**	**1.21**	**1.99**	**3.72**	**0.44**	**0.65**
NPTC infant posture/action	**13.87**	**9.87**	**3.13**	**2.13**	**11.49**	**10.94**	**1.17**	**1.03**	**0.91**	**3.99**	**3.96**	**2.44**	**0.81**	**7.26**
Infant touch R	76.84***	79.13***	68.17***	66.01***	76.23***	49.36***	43.11***	34.77***	38.11***	22.07***	15.41**	14.79**	**4.23**	**3.51**
No infant touch R	36.98***	35.22***	39.58***	42.41***	41.43***	19.41***	20.57***	25.16***	26.10***	**8.36**	**10.63***	**12.58***	**2.23**	**3.19**
Not possible to code touch R	66.47***	67.95***	74.92***	77.18***	67.61***	36.10***	40.90***	48.56***	43.06***	17.74**	22.18***	21.20***	**5.60**	**5.51**
Infant touch L	72.04***	69.03***	68.14***	67.27***	63.42***	43.39***	37.80***	37.43***	34.02***	19.59***	16.08**	14.71**	**5.28**	**2.98**
No infant touch L	40.07***	48.29***	42.80***	44.05***	45.15***	25.90***	26.93***	21.84***	25.76***	**10.71***	**11.45***	**9.71***	**2.11**	**3.77**
Not possible to code touch L	69.24***	72.65***	76.85***	78.42***	74.97***	37.05***	40.09***	46.66***	45.24***	15.82**	20.96***	22.12***	**4.57**	**5.97**
Physical play evident	63.86***	72.47***	67.95***	55.61***	62.36***	41.96***	42.69***	33.21***	32.27***	21.31***	16.84***	13.45**	**4.20**	**4.27**
No physical play	106.39***	103.65***	115.16***	106.99***	97.23***	56.01***	63.59***	67.83***	57.52***	27.90***	30.88***	28.06***	**8.21***	**7.33***
NPTC physical play	14.55***	3.85	0.34	16.34***	7.43*	6.75*	1.78	0.11	10.49**	**0.52**	**1.43**	**0.05**	**0.11**	**1.13**
Laughing	**3.55**	**0.45**	**1.53**	**1.05**	**0.20**	**0.45**	**0.33**	**0.74**	**0.13**	**0.33**	**0.25**	**0.07**	**0.25**	**0.00**
Distressed	**1.61**	**0.22**	**1.73**	**1.61**	**4.50**	**0.01**	**0.27**	**0.07**	**1.61**	**0.07**	**0.10**	**0.07**	**0.00**	**0.10**
Non-Distress	**8.49**	**8.70**	**8.26**	**8.40**	**8.35**	**4.82**	**5.12**	**4.23**	**4.64**	**2.25**	**2.00**	**1.82**	**0.51**	**0.52**
Imitating sounds	**0.17**	**0.47**	**1.34**	**2.89**	**0.50**	**0.06**	**0.41**	**1.34**	**0.50**	**0.03**	**0.41**	**0.73**	**0.03**	**0.29**
Babbling	**0.88**	**2.02**	**0.56**	**1.99**	**2.92**	**0.43**	**0.81**	**0.80**	**1.25**	**0.09**	**0.57**	**0.53**	**0.10**	**0.35**
First words	**1.20**	**0.37**	**0.42**	**0.52**	**0.52**	**0.47**	**0.02**	**0.28**	**0.52**	**0.07**	**0.01**	**0.20**	**0.00**	**0.02**
Screaming	**1.00**	**1.00**	**1.00**	**1.00**	**0.00**	**1.00**	**1.00**	**1.00**	**0.00**	**1.00**	**1.00**	**0.00**	**1.00**	**0.00**
Bodily Sounds	**1.21**	**0.67**	**0.11**	**0.97**	**0.19**	**0.78**	**0.14**	**0.30**	**0.12**	**0.27**	**0.06**	**0.10**	**0.07**	**0.02**
Silent/none of the above	175.77***	176.54***	176.98***	177.88***	176.18***	98.67***	99.58***	100.65***	100.01***	43.94***	44.98***	44.8***	**11.28**	**11.39**
NPTC infant vocalisation	**6.00**	**0.00**	**6.00**	**6.00**	**6.00**	**0.00**	**0.00**	**6.00**	**6.00**	**0.00**	**0.00**	**6.00**	**0.00**	**0.00**

**p* < 0.05; ***p* < 0.01; ****p* < 0.001. NPTC, not possible to code; BO, body orientation. From top to bottom, behavioural groups shown are: Body Orientation, Visual Attention, Posture, Touch R, Touch L, Physical Play, and Vocalisation. Only behaviours that occurred within this population are included. Behaviours have been arranged according to the corresponding behavioural groups. The bold values highlight an example criterion which could be used to identify an appropriate length of time to code for each behaviour, if wanting to investigate transitions between behaviours: i.e. where all chi-squared values for a given slice length have *p* < 0.05. Fully-boldened regions represent the corresponding “cut off”, where *p* < 0.05 for all slices to the right of the line; indicating that the slice length rightward adjacent to the black line would be an appropriate coding timeframe for the given behavioural group. E.g., data within the first rows suggest that coding 4-min of Body Orientation would be suitable

**Table 15:** Bristol mums: chi-squared test on absolute differences between thin slice and full session transition matrices

Behaviour (by category)	Slice (by length)													
	1-min					2-min				3-min			4-min	
	One	Two	Three	Four	Five	OneTwo	TwoThree	ThreeFour	FourFive	OneTwo Three	TwoThree Four	ThreeFour Five	OneTwo ThreeFour	TwoThree FourFive
Out of reach	33.64***	54.87***	34.83***	12.04**	60.62***	30.38***	28.02***	5.81	0.68	19.93***	4.43	2.63	**6.85**	**3.39**
Within reach	168.52***	168.90***	164.22***	167.41***	160.23***	99.98***	91.83***	90.76***	92.99***	43.37***	39.58***	38.83***	**10.86***	**8.98***
Loom	11.49**	3.91	7.39	13.91**	9.77*	**3.95**	**4.03**	**4.04**	**7.96***	**2.28**	**2.09**	**0.77**	**0.85**	**0.34**
NPTC caregiver proximity	27.40***	39.02***	83.30***	96.25***	92.93***	10.11*	29.51***	83.30***	92.93***	9.72*	15.31**	81.44***	**2.50**	**5.54**
BO to infant	156.98***	155.21***	151.11***	156.13***	146.25***	85.36***	87.86***	81.66***	87.7***	42.99***	36.57***	34.94***	**10.47***	**7.97**
BO to sibling (c)	**4.00**	**0.00**	**4.00**	**4.00**	**4.00**	**0.00**	**0.00**	**4.00**	**4.00**	**0.00**	**0.00**	**4.00**	**0.00**	**0.00**
BO to other person/object	**11.28***	**1.58**	**11.24***	**7.49**	**11.28***	**2.08**	**1.68**	**5.80**	**6.15**	**1.66**	**0.22**	**4.64**	**0.04**	**0.25**
BO to object (focus of the activity)	52.90***	50.73***	22.46***	40.90***	10.43*	39.71***	15.82**	10.40*	5.15	19.44***	9.08	1.43	**6.50**	**0.21**
NPTC caregiver BO	101.32***	95.48***	96.67***	108.57***	95.05***	50.61***	49.03***	58.35***	57.48***	23.11***	31.90***	23.09***	**5.80**	**10.65***
Vis-à-vis—infant and caregiver	117.62***	94.37***	97.90***	109.89***	105.68***	60.59***	50.32***	56.68***	56.18***	28.93***	22.26***	20.66***	**9.06**	**2.99**
Slight (30°–90°) aversion right	19.37**	0.49	7.74	5.54	15.50**	**14.99***	**1.62**	**5.26**	**5.54**	**0.77**	**1.83**	**5.26**	**0.00**	**1.83**
Slight (30°–90°) aversion left	**1.60**	**0.90**	**0.40**	**1.60**	**3.00**	**0.35**	**0.35**	**0.10**	**1.60**	**0.40**	**0.07**	**0.10**	**0.00**	**0.07**
Full (90°) aversion right	**11.77***	**12.67***	**6.09**	**2.54**	**7.87**	**11.77***	**6.09**	**0.95**	**1.14**	**5.24**	**0.95**	**0.35**	**0.54**	**0.35**
Full (90°) aversion left	**0.00**	**5.00**	**5.00**	**5.00**	**5.00**	**0.00**	**5.00**	**5.00**	**5.00**	**0.00**	**5.00**	**5.00**	**0.00**	**5.00**
NPTC head orientation	115.08***	120.00***	126.83***	116.85***	113.20***	59.21***	70.76***	60.09***	57.47***	32.22***	26.80***	20.56***	**7.45**	**4.74**
Look at infant	73.56***	73.06***	54.27***	63.34***	69.87***	43.75***	34.31***	21.36*	34.68***	**15.32**	**13.41**	**6.61**	**2.95**	**4.05**
Look at same object/joint attention	**1.00**	**1.00**	**1.00**	**1.00**	**0.00**	**1.00**	**1.00**	**1.00**	**0.00**	**1.00**	**1.00**	**0.00**	**1.00**	**0.00**
Look at focus object	**4.75**	**11.38**	**4.09**	**12.11**	**8.90**	**4.14**	**2.69**	**6.51**	**4.58**	**1.66**	**4.78**	**2.70**	**0.71**	**1.73**
Look at different object	**0.00**	**1.00**	**1.00**	**1.00**	**0.00**	**0.00**	**1.00**	**1.00**	**0.00**	**0.00**	**1.00**	**0.00**	**0.00**	**0.00**
Look at other object	45.37***	28.96**	67.2***	55.30***	58.02***	25.40**	12.77	55.30***	47.69***	12.6	7.42	47.69***	**3.19**	**1.26**
Look at object outside of view	**19.57***	**10.30**	**14.43**	**8.15**	**1.99**	**9.69**	**7.60**	**8.50**	**4.84**	**7.57**	**5.82**	**0.21**	**4.31**	**0.19**
Look at sibling	**14.00**	**13.00**	**12.50**	**6.46**	**3.96**	**13.00**	**11.50**	**4.96**	**1.50**	**11.50**	**3.96**	**1.00**	**3.96**	**0.00**
Look at other person	**0.00**	**3.00**	**3.00**	**3.00**	**3.00**	**0.00**	**3.00**	**3.00**	**3.00**	**0.00**	**3.00**	**3.00**	**0.00**	**3.00**
Look at distraction	**0.00**	**8.00**	**8.00**	**8.00**	**8.00**	**0.00**	**8.00**	**8.00**	**8.00**	**0.00**	**8.00**	**8.00**	**0.00**	**8.00**
NPTC visual attention	106.18***	97.49***	100.07***	105.20***	100.20***	55.22***	51.80***	51.61***	55.75***	21.73*	24.46**	22.10*	**8.06**	**3.40**
Neutral/Alert	41.92***	46.53***	49.24***	46.61***	41.38***	24.05**	27.22***	28.87***	23.93**	**12.91**	**14.14***	**13.01**	**3.29**	**3.88**
Positive	**7.320**	**6.38**	**6.33**	**4.10**	**5.96**	**3.37**	**3.19**	**2.49**	**1.48**	**1.96**	**0.70**	**0.88**	**0.36**	**0.15**
Smile	**13.15**	**13.19**	**8.85**	**11.10**	**13.64**	**8.81**	**5.35**	**5.29**	**7.02**	**3.23**	**2.92**	**2.35**	**0.75**	**0.88**
Negative	**1.28**	**3.94**	**5.50**	**2.83**	**1.28**	**10.61**	**0.61**	**2.83**	**1.28**	**1.28**	**0.44**	**1.18**	**0.29**	**0.11**
Disgust	**2.00**	**2.00**	**2.00**	**2.00**	**0.00**	**2.00**	**2.00**	**2.00**	**0.00**	**2.00**	**2.00**	**0.00**	**2.00**	**0.00**
Mock surprise	**5.50**	**2.21**	**4.58**	**4.21**	**3.63**	**1.10**	**1.22**	**2.88**	**2.37**	**0.59**	**0.60**	**2.12**	**0.10**	**0.38**
Face not visible	120.46***	128.30***	132.85***	131.89***	117.76***	68.46***	76.12***	78.50***	68.75***	34.73***	34.13***	34.60***	**9.12**	**8.10**
None of the above	**2.82**	**4.25**	**4.25**	**0.87**	**1.99**	**2.82**	**4.25**	**1.37**	**0.31**	**1.89**	**1.37**	**0.31**	**0.43**	**0.31**
Sit on floor	167.39***	176.71***	148.49***	148.49***	148.49***	127.39***	90.84***	70.95***	70.95***	56.62***	33.28***	21.73***	14.15**	4.03
Sit on object	166.81***	157.26***	156.61***	157.37***	156.61***	94.16***	78.20***	78.31***	78.31***	41.79***	26.85***	26.72***	**10.52***	**2.46**
Stand up	**0.50**	**0.50**	**4.50**	**4.50**	**4.50**	**0.00**	**0.50**	**4.50**	**4.50**	**0.00**	**0.50**	**4.50**	**0.00**	**0.50**
NPTC caregiver posture/action	51.99***	22.20***	18.70***	18.70***	18.7***	19.66***	3.56	3.56	3.56	8.74	2.39	58.31***	2.18	18.70***
Caregiver touch R	90.73***	88.26***	90.33***	93.20***	96.46***	54.92***	50.05***	59.17***	53.57***	25.90***	21.55***	28.73***	**7.06***	**3.96**
No caregiver touch R	10.70**	1.79	11.55**	13.40**	4.74	4.14	0.40	10.33**	4.32	**3.04**	**0.91**	**3.18**	**1.60**	**0.13**
NPTC touch R	116.74***	122.27***	112.98***	115.31***	117.53***	68.62***	65.97***	57.12***	64.26***	28.60***	27.96***	23.39***	**6.33***	**7.71***
Caregiver touch L	58.00***	66.39***	62.50***	61.62***	56.47***	43.85***	38.00***	41.21***	34.45***	19.79***	15.52***	22.02***	**4.99**	**3.01**
No caregiver touch L	31.87***	3.84	36.67***	31.18***	12.57**	14.63***	11.84**	26.22***	2.67	12.15**	6.59*	1.25	**8.36***	**5.05**
NPTC touch L	140.00***	138.17***	136.17***	137.75***	138.26***	75.18***	76.71***	72.10***	76.92***	32.20***	33.90***	30.53***	7.53*	9.54**
Reaching	**2.45**	**1.31**	**5.50**	**0.74**	**5.50**	**1.81**	**1.31**	**0.74**	**0.74**	**1.81**	**2.45**	**0.74**	**0.00**	**2.45**
Clapping	**15.00***	**0.00**	**15.00***	**15.00***	**15.00***	**0.00**	**0.00**	**15.00***	**15.00***	**0.00**	**0.00**	**15.00***	**0.00**	**0.00**
Banging	21.78***	6.16	10.67	13.57*	13.42*	**3.12**	**0.06**	**2.79**	**8.38**	**0.52**	**2.79**	**3.53**	**4.66**	**0.19**
Other hand movements	26.17***	27.50***	2.25	17.15**	2.25	26.17***	2.25	0.91	0.91	8.35	0.91	26.17***	5.19	26.17***
No hand movements	145.75***	134.01***	136.95***	135.11***	145.33***	83.56***	65.12***	67.98***	85.96***	38.72***	20.13**	43.19***	**10.24**	**9.42**
NPTC hand movements	117.14***	97.04***	97.04***	107.88***	103.82***	58.22***	31.49***	45.47***	49.41***	19.72**	9.74	11.53*	**6.93**	**0.31**
Physical play evident	62.64***	61.20***	54.90***	86.23***	67.79***	23.17***	34.29***	33.22***	43.42***	18.92***	17.71***	9.42**	**7.29***	**2.29**
No physical play	166.79***	167.02***	176.94***	167.47***	172.72***	90.90***	98.39***	98.73***	95.61***	42.61***	42.95***	45.9***	10.0**	11.50**
NPTC physical play	193.29***	190.08***	190.08***	190.08***	190.08***	108.72***	106.32***	106.32***	106.32***	48.32***	46.72***	46.72***	12.08**	11.29**
Speech	47.06***	57.66***	56.16***	53.03***	60.18***	27.94***	35.18***	31.43***	31.31***	**13.16**	**16.35***	**15.33***	**3.06**	**5.37**
Musical Sounds	9.83	3.61	0.45	10.33	2.06	0.53	18.56**	0.45	2.06	0.51	18.56**	0.78	**0.51**	**2.46**
Laugh	**2.50**	**3.48**	**1.69**	**3.53**	**1.68**	**1.74**	**1.31**	**1.51**	**1.21**	**0.85**	**1.14**	**0.35**	**0.33**	**0.27**
Vocal imitation	**2.99**	**1.42**	**3.16**	**4.56**	**5.19**	**3.75**	**0.43**	**2.24**	**3.51**	**0.51**	**0.29**	**1.72**	**0.21**	**0.12**
Bodily sounds	**3.99**	**2.09**	**4.22**	**0.88**	**5.60**	**0.66**	**1.16**	**1.34**	**0.88**	**0.94**	**0.14**	**1.34**	**0.00**	**0.14**
Scream	**1.00**	**1.00**	**0.00**	**1.00**	**1.00**	**1.00**	**0.00**	**0.00**	**1.00**	**0.00**	**0.00**	**0.00**	**0.00**	**0.00**
Non-verbal sound	**5.68**	**4.05**	**5.19**	**5.56**	**6.54**	**1.88**	**1.86**	**3.64**	**5.14**	**0.47**	**1.22**	**2.65**	**0.21**	**0.46**
Silent	130.24***	121.98***	121.87***	121.02***	113.42***	74.33***	67.93***	67.23***	64.96***	33.96***	29.14***	27.64***	**8.92**	**6.01**

**p* < 0.05; ***p* < 0.01; ****p* < 0.001. NPTC, not possible to code; BO, body orientation. From top to bottom, behavioural groups shown are: Proximity, Body Orientation, Head Orientation, Visual Attention, Facial Expression, Posture, Touch R, Touch L, Hand Movements, Physical Play, and Vocalisation. Only behaviours that occurred within this population are included. Behaviours have been arranged according to the corresponding behavioural groups. The bold values highlight an example criterion which could be used to identify an appropriate length of time to code for each behaviour, if wanting to investigate transitions between behaviours: i.e. where all chi-squared values for a given slice length have *p* < 0.05. Fully-boldened regions represent the corresponding “cut off”, where *p* < 0.05 for all slices to the right of the line; indicating that the slice length rightward adjacent to the black line would be an appropriate coding timeframe for the given behavioural group. E.g., data within the first rows suggest that coding 4-min of Proximity would be suitable

**Table 16:** Bristol infants (1): chi-squared test on absolute differences between thin slice and full session transition matrices

Behaviour (by category)	Slice (by length)													
	1-min					2-min				3-min			4-min	
	One	Two	Three	Four	Five	OneTwo	TwoThree	ThreeFour	FourFive	OneTwo Three	TwoThree Four	ThreeFour Five	OneTwo ThreeFour	TwoThree FourFive
BO to caregiver 1	119.68***	113.64***	104.96***	106.76***	110.51***	64.54***	47.00***	42.40***	53.57***	22.83***	9.59*	16.42***	**2.39**	**1.27**
BO to different person/object	**2.94**	**0.28**	**10.50***	**10.50***	**10.50***	**0.00**	**0.28**	**10.50***	**10.50***	**0.00**	**0.28**	**10.50***	**0.00**	**0.28**
BO to object (focus of activity)	69.00***	63.41***	70.42***	78.80***	73.90***	28.51***	39.87***	38.38***	40.85***	20.64***	24.39***	13.70**	**4.07**	**8.57***
NPTC infant body orientation	135.64***	118.62***	109.69***	116.24***	118.86***	72.64***	62.34***	52.75***	58.88***	30.54***	26.02***	17.27***	**7.47**	**5.64**
Vis-à-vis—infant and caregiver	75.93***	61.03***	53.74***	65.18***	69.36***	40.39***	26.51***	24.09***	37.71***	**13.43***	**10.67**	**11.41**	**4.46**	**1.01**
Slight (30°–90°) aversion right	**7.69**	**10.50**	**11.25**	**0.54**	**2.64**	**9.08**	**9.81**	**1.04**	**3.49**	**8.83**	**0.79**	**0.36**	**1.43**	**0.48**
Slight (30°–90°) aversion left	7.88	2.03	17.57**	20.46**	27.54***	1.39	2.84	16.22*	19.88**	**0.15**	**2.29**	**15.95***	**0.03**	**2.18**
Full (90°) aversion right	**9.50**	**9.50**	**9.50**	**1.07**	**1.07**	**9.50**	**9.50**	**3.39**	**9.50**	**9.50**	**3.39**	**0.00**	**3.39**	**0.00**
Full (90°) aversion left	9.33	9.33	27.50***	27.50***	26.17***	26.17***	9.33	27.50***	26.17***	26.17***	9.33	26.17***	26.17***	15.46*
Arch aversion	**2.00**	**0.50**	**2.00**	**2.00**	**0.50**	**0.50**	**0.50**	**2.00**	**0.50**	**0.50**	**0.50**	**0.50**	**0.50**	**0.00**
NPTC head orientation	136.28***	135.97***	134.63***	140.55***	134.25***	73.70***	81.76***	82.10***	75.60***	39.32***	36.46***	34.84***	**10.64**	**7.83**
Look at caregiver 1	42.55***	38.70***	42.10***	46.62***	47.35***	19.09*	21.07**	21.72**	27.89***	**9.60**	**8.74**	**11.09**	**2.68**	**1.37**
Look at focus object	33.02***	43.21***	36.28***	36.46***	33.30***	23.42**	26.71***	15.23	16.97*	**11.58**	**12.72**	**2.65**	**3.25**	**3.30**
Look at different object	**0.00**	**3.00**	**3.00**	**3.00**	**3.00**	**0.00**	**3.00**	**3.00**	**3.00**	**0.00**	**3.00**	**3.00**	**0.00**	**3.00**
Look at other object	37.75***	8.19	18.22*	11.07	37.35***	20.98**	3.28	3.32	4.50	**12.52**	**1.34**	**0.55**	**0.90**	**4.27**
Look at object outside of view	18.09*	23.40**	27.81***	12.15	8.20	**13.33**	**19.63***	**10.53**	**1.62**	**9.88**	**7.22**	**1.83**	**2.52**	**1.07**
Look at sibling	**5.33**	**7.00**	**7.00**	**4.33**	**2.33**	**5.33**	**7.00**	**4.33**	**0.33**	**5.33**	**4.33**	**0.33**	**3.33**	**0.33**
No visual attention	**4.42**	**2.87**	**0.47**	**4.42**	**5.67**	**1.65**	**1.17**	**0.40**	**4.42**	**0.08**	**1.10**	**0.400**	**0.00**	**1.10**
NPTC visual attention	111.02***	95.44***	92.34***	102.04***	99.66***	56.86***	42.83***	49.32***	58.10***	21.27**	11.67	23.52**	**3.12**	**1.85**
Neutral/Alert	30.59***	31.96***	31.31***	32.79***	33.93***	**16.38***	**15.22***	**15.21***	**19.64****	**8.52**	**4.67**	**7.68**	**1.61**	**1.45**
Positive	**11.64**	**7.85**	**4.30**	**8.25**	**4.34**	**7.59**	**3.43**	**1.92**	**2.21**	**3.14**	**1.71**	**0.80**	**1.67**	**0.44**
Smile	**3.79**	**6.09**	**0.63**	**4.50**	**8.50**	**2.08**	**0.57**	**0.80**	**4.12**	**0.28**	**0.18**	**0.74**	**0.03**	**0.24**
Negative	**1.51**	**4.00**	**2.88**	**0.41**	**7.58**	**1.51**	**2.88**	**0.36**	**0.76**	**1.63**	**0.36**	**0.14**	**0.44**	**0.14**
Disgust	**1.00**	**1.00**	**0.00**	**1.00**	**1.00**	**1.00**	**0.00**	**0.00**	**1.00**	**0.00**	**0.00**	**0.00**	**0.00**	**0.00**
Face not visible	153.32***	142.51***	145.32***	141.44***	145.08***	84.59***	81.23***	77.90***	80.73***	39.38***	35.31***	37.67***	**10.24**	**7.58**
None of the above	**13.52***	**4.41**	**2.36**	**13.18***	**11.37**	**4.46**	**1.40**	**3.25**	**11.27**	**1.07**	**1.43**	**1.63**	**0.76**	**0.33**
Lie down	1.05	19.05**	22.00**	22.00**	22.00**	0.00	19.05**	22.00**	22.00**	0.00	19.05**	22.00**	0.00	19.05**
Sit on the floor	145.33***	171.33***	158.73***	197.05***	128.98***	97.27***	95.35***	128.98***	102.34***	42.58***	51.60***	55.44***	**15.48***	**10.83**
Sit on an object	167.41***	156.35***	157.11***	156.35***	156.35***	94.07***	78.01***	78.01***	77.51***	42.02***	26.35***	26.35***	**10.51**	**2.13**
Standing	**10.00**	**10.00**	**10.00**	**0.00**	**10.00**	**10.00**	**10.00**	**0.00**	**0.00**	**10.00**	**0.00**	**0.00**	**0.00**	**0.00**
Crawling	49.00***	49.00***	19.75**	6.75	49.00***	49.00***	19.75**	0.00	6.75	19.75**	0.00	0.00	**0.00**	**0.00**
Held/in hold	192.00***	192.00***	192.00***	192.00***	192.00***	108.00***	108.00***	108.00***	108.00***	48.00***	48.00***	48.00***	**12.00**	**12.00**
Try to move in another way	**3.00**	**3.00**	**0.00**	**3.00**	**3.00**	**3.00**	**0.00**	**0.00**	**3.00**	**0.00**	**0.000**	**0.00**	**0.00**	**0.00**
NPTC infant posture/action	82.88***	79.64***	73.49***	73.49***	73.49***	47.23***	44.13***	16.47*	16.47*	20.99**	19.11**	1.42	**5.25**	**4.59**
Infant touch R	10.01**	6.86*	4.64	10.52**	19.69***	1.35	0.78	1.08	11.61**	**0.05**	**0.17**	**3.14**	**0.78**	**1.12**
No infant touch R	25.43***	33.49***	25.36***	17.88***	7.84*	22.49***	22.56***	12.79**	2.76	12.24**	10.76**	2.96	**4.05**	**1.93**
NPTC touch R	162.52***	153.53***	155.32***	154.85***	150.70***	88.2***	86.81***	83.88***	80.53***	39.93***	38.82***	32.37***	10.44**	9.06*
Infant touch L	11.87**	3.79	25.10***	7.63*	6.50*	2.51	7.65*	12.49**	14.63***	**2.45**	**2.47**	**5.94**	**0.45**	**0.49**
No infant touch L	19.20***	22.38***	4.60	16.58***	7.30*	15.28***	5.04	9.65**	5.22	**4.33**	**6.57***	**1.61**	**3.61**	**0.74**
NPTC touch L	172.32***	154.21***	160.55***	162.42***	159.98***	96.40***	87.27***	88.27***	93.77***	41.47***	36.39***	40.1***	9.76**	8.57*
Reaching	**2.18**	**8.38**	**1.91**	**5.58**	**2.31**	**1.81**	**1.87**	**2.62**	**2.52**	**0.84**	**2.49**	**1.32**	**0.39**	**1.40**
Waving	**0.00**	**5.00**	**5.00**	**5.00**	**5.00**	**0.00**	**5.00**	**5.00**	**5.00**	**0.00**	**5.00**	**5.00**	**0.00**	**5.00**
Gesticulating	**1.00**	**1.00**	**0.00**	**1.00**	**1.00**	**1.00**	**0.00**	**0.00**	**1.00**	**0.00**	**0.00**	**0.00**	**0.00**	**0.00**
Banging	27.86***	1.91	16.46*	22.73***	9.27	**8.13**	**5.80**	**12.47**	**5.03**	**5.05**	**3.60**	**3.32**	**2.93**	**0.09**
Other hand movements	58.56***	81.21***	53.76***	23.96***	70.35***	55.11***	51.57***	8.50	15.60*	30.60***	7.40	4.11	**1.35**	**3.56**
No hand movements	19.65**	7.04	23.48***	31.85***	11.24	**10.84**	**8.05**	**14.89***	**4.71**	**5.37**	**4.59**	**0.62**	**3.25**	**5.85**
NPTC hand movements	151.30***	139.19***	142.97***	155.55***	144.39***	71.72***	65.80***	89.57***	84.45***	22.79***	37.83***	38.41***	**11.23**	**8.17**
Physical play evident	23.14***	6.36*	32.97***	25.18***	35.33***	5.03	9.58**	10.46**	11.83**	7.63*	0.52	3.03	**1.18**	**1.14**
No physical play	112.8***	126.51***	112.31***	128.06***	111.36***	62.43***	62.12***	63.25***	62.58***	20.58***	34.91***	20.67***	**8.9***	**8.58***
NPTC physical play	142.46***	139.01***	138.26***	154.74***	138.73***	67.86***	64.51***	89.46***	89.79***	20.07***	37.04***	42.3***	9.77**	8.77*
Laughing	2.00	2.00	1.00	1.00	2.00	**2.00**	**1.0**	**0.00**	**1.00**	**1.00**	**0.00**	**0.00**	**0.00**	**0.00**
Distressed	11.11	28.65***	37.26***	16.75*	25.7***	**10.84**	**16.65***	**13.86**	**8.35**	**4.14**	**4.53**	**7.71**	**0.31**	**2.07**
Non-Distress	36.06***	32.43***	28.22***	40.48***	24.28**	18.47*	15.34*	22.47**	17.99*	**6.39**	**9.55**	**7.49**	**3.26**	**1.43**
Imitating sounds	**2.00**	**2.00**	**2.00**	**2.00**	**0.00**	**2.00**	**2.00**	**2.00**	**0.00**	**2.00**	**2.00**	**0.00**	**2.00**	**0.00**
Babbling	**6.00**	**6.00**	**6.00**	**4.20**	**4.20**	**6.00**	**6.00**	**4.20**	**0.00**	**6.00**	**4.20**	**0.00**	**4.20**	**0.00**
Screaming	**14.33***	**12.83**	**14.33***	**0.88**	**7.25**	**12.83**	**12.83**	**0.88**	**3.21**	**12.83**	**0.85**	**3.21**	**0.85**	**0.00**
Bodily Sounds	8.45	10.82	11.73	22.8**	19.46**	**3.74**	**4.44**	**9.99**	**15.51***	**1.50**	**3.08**	**5.01**	**0.85**	**0.84**
Silent/none of the above	99.08***	106.31***	101.34***	114.86***	119.92***	54.29***	46.74***	60.47***	66.98***	15.30*	25.4***	27.47***	**6.51**	**6.35**

**p* < 0.05; ***p* < 0.01; ****p* < 0.001. NPTC, not possible to code; BO, body orientation. From top to bottom, behavioural groups shown are: Body Orientation, Head Orientation, Visual Attention, Facial Expression, Posture, Touch R, Touch L, Hand Movements, Physical Play, and Vocalisation. Only behaviours that occurred within this population are included. Behaviours have been arranged according to the corresponding behavioural groups. The bold values highlight an example criterion which could be used to identify an appropriate length of time to code for each behaviour, if wanting to investigate transitions between behaviours: i.e. where all chi-squared values for a given slice length have *p* < 0.05. Fully-boldened regions represent the corresponding “cut off”, where *p* < 0.05 for all slices to the right of the line; indicating that the slice length rightward adjacent to the black line would be an appropriate coding timeframe for the given behavioural group. E.g., data within the first rows suggest that coding 4-min of Body Orientation would be suitable

**Table 17:** Bristol dads: chi-squared test on absolute differences between thin slice and full session transition matrices

Behaviour (by category)	Slice (by length)													
	1-min					2-min				3-min			4-min	
	One	Two	Three	Four	Five	OneTwo	TwoThree	ThreeFour	FourFive	OneTwo Three	TwoThree Four	ThreeFour Five	OneTwo ThreeFour	TwoThree FourFive
Out of reach	41.40***	46.83***	48.12***	9.80*	48.32***	26.70***	37.05***	7.49	3.56	29.52***	3.43	0.48	**4.95**	**1.46**
Within reach	137.66***	128.73***	133.34***	130.14***	125.15***	78.98***	68.85***	69.79***	63.91***	36.02***	26.07***	28.83***	**10.28***	**5.46**
Loom	69.38***	42.31***	60.50***	59.85***	77.7***	23.68***	20.85***	30.84***	41.66***	10.6*	5.53	20.53***	**1.12**	**1.72**
NPTC caregiver proximity	52.86***	30.73***	41.49***	21.08***	41.32***	24.72***	14.83**	6.95	6.79	15.24**	1.46	2.59	**3.87**	**4.73**
BO to infant	131.37***	128.86***	122.07***	123.95***	117.54***	73.42***	63.6***	60.46***	62.94***	29.34***	24.35***	23.35***	**6.64**	**4.82**
BO to other caregiver	15.77**	32.76***	45.00***	4.95	9.21	12.37*	32.76***	4.95	58.99***	12.37*	10.86*	58.99***	**5.26**	**2.09**
BO to other person/object	5.37	4.50	6.09	8.00	28.50***	**5.02**	**5.49**	**1.09**	**13.12***	**5.24**	**0.82**	**3.82**	**2.07**	**5.38**
BO to object (focus object)	**2.15**	**9.42**	**3.36**	**12.33***	**4.79**	**4.59**	**0.42**	**1.7**	**4.45**	**1.11**	**0.09**	**0.36**	**0.70**	**2.15**
NPTC caregiver body orientation	142.17***	114.82***	115.22***	107.75***	92.39***	83.56***	69.36***	51.96***	45.06***	40.35***	27.42***	12.93*	**10.62***	**4.17**
Vis-à-vis—infant and caregiver	121.85***	106.90***	102.81***	99.45***	101.79***	72.62***	50.82***	45.90***	48.10***	32.33***	14.24*	14.12*	**7.30**	**0.80**
Slight (30°–90°) aversion right	**1.97**	**2.89**	**3.52**	**3.10**	**0.76**	**0.54**	**2.37**	**1.53**	**0.25**	**1.03**	**0.73**	**0.04**	**0.36**	**0.02**
Slight (30°–90°) aversion left	25.87***	19.17**	29.51***	30.45***	37.71***	12.68*	15.70*	14.25*	24.59***	**4.03**	**6.17**	**8.86**	**0.43**	**3.18**
Full (90°) aversion right	11.85	9.07	7.40	18.25**	6.27	**5.52**	**1.27**	**5.65**	**5.83**	**1.32**	**0.52**	**2.38**	**1.07**	**0.46**
Full (90°) aversion left	**0.36**	**0.57**	**0.57**	**5.00**	**5.00**	**0.06**	**0.57**	**0.57**	**5.00**	**0.00**	**0.57**	**0.57**	**0.00**	**0.57**
Head not in view of infant	15.92*	30.77***	18.41**	13.00*	4.04	13.92*	21.88**	8.89	7.26	**5.75**	**14.26***	**6.59**	**2.30**	**5.25**
NPTC head orientation	62.90***	35.68***	43.14***	33.86***	37.98***	33.66***	9.83	10.10	8.57	**17.01****	**0.27**	**1.74**	**5.95**	**0.92**
Look at infant	99.15***	85.27***	85.05***	87.06***	85.89***	53.18***	48.66***	44.73***	48.96***	**21.33***	**19.53***	**20.53***	**4.16**	**5.95**
Look at caregiver 2	36.96***	34.19***	24.16**	19.63*	22.40**	25.23**	16.57	3.79	3.00	**12.20**	**3.94**	**2.42**	**2.70**	**0.24**
Look at focus object	**11.70**	**17.72***	**15.26**	**17.01***	**14.62**	**8.04**	**10.48**	**9.27**	**9.71**	**3.83**	**6.37**	**4.40**	**1.28**	**1.86**
Look at different object	**0.69**	**3.00**	**3.00**	**1.75**	**4.00**	**0.69**	**3.00**	**1.75**	**0.94**	**0.69**	**1.75**	**0.94**	**0.33**	**0.94**
Look at other object	**4.77**	**12.50**	**3.79**	**2.65**	**4.24**	**3.25**	**3.80**	**1.69**	**1.04**	**0.99**	**1.61**	**0.85**	**0.27**	**1.06**
Look at object outside of view	**7.18**	**4.66**	**9.60**	**7.22**	**3.96**	**2.11**	**4.10**	**5.84**	**2.43**	**1.79**	**2.25**	**1.69**	**0.67**	**0.71**
Look at sibling	31.34***	26.09**	35.01***	28.33***	22.57**	20.05*	22.71**	23.95**	14.17	**10.22**	**11.88**	**10.05**	**3.37**	**3.29**
Look at distraction	**0.00**	**8.00**	**8.00**	**8.00**	**8.00**	**0.00**	**8.00**	**8.00**	**8.00**	**0.00**	**8.00**	**8.00**	**0.00**	**8.00**
NPTC visual attention	96.83***	68.69***	82.33***	61.55***	68.13***	56.77***	36.83***	37.26***	25.81**	29.21***	9.77	11.67	**8.88**	**0.71**
Neutral/Alert	25.59***	31.51***	31.65***	38.91***	27.31***	11.54	19.35**	24.41***	19.99**	**5.24**	**14.05**	**13.53**	**1.99**	**5.59**
Positive	**9.91**	**8.92**	**5.70**	**7.29**	**11.73**	**6.30**	**2.43**	**1.26**	**7.18**	**1.61**	**0.36**	**1.05**	**0.17**	**0.27**
Smile	**10.76**	**11.67**	**11.13**	**7.52**	**9.49**	**6.98**	**6.74**	**4.99**	**2.32**	**4.58**	**2.07**	**0.65**	**1.49**	**0.30**
Negative	**4.00**	**4.00**	**4.00**	**4.00**	**0.00**	**4.00**	**4.00**	**4.00**	**0.00**	**4.00**	**4.00**	**0.00**	**4.00**	**0.00**
Surprise	**2.00**	**2.00**	**1.00**	**1.00**	**2.00**	**2.00**	**1.00**	**0.00**	**1.00**	**1.00**	**0.00**	**0.00**	**0.00**	**0.00**
Mock surprise	**1.42**	**1.74**	**2.74**	**2.00**	**3.47**	**1.54**	**1.64**	**0.72**	**1.48**	**1.32**	**0.13**	**0.98**	**0.07**	**0.07**
Face not visible	144.24***	132.25***	131.82***	130.76***	127.34***	85.90***	67.77***	66.69***	67.00***	38.34***	24.30**	24.18**	**9.46**	**2.88**
None of the above	**7.00**	**7.00**	**7.00**	**7.00**	**0.0**	**7.00**	**7.00**	**7.00**	**0.00**	**7.00**	**7.00**	**0.00**	**7.0**	**0.00**
Lie on side	203.53***	175.72***	175.72***	175.72***	175.72***	114.49***	93.88***	93.88***	93.88***	50.88***	37.49***	37.49***	**12.72***	**6.53**
Sit on floor	**0.00**	**7.00**	**7.00**	**7.00**	**7.00**	**0.00**	**7.00**	**7.00**	**7.00**	**0.00**	**7.00**	**7.00**	**0.00**	**7.00**
Sit on object	145.72***	130.02***	138.07***	127.69***	133.34***	77.04***	67.41***	70.9***	57.95***	36.02***	23.82***	28.04***	**8.48**	**3.11**
Stand up	29.59***	17.89**	32.32***	2.22	16.79**	14.78*	18.57**	10.78	3.25	**10.94**	**6.34**	**7.58**	**4.32**	**1.59**
Crouched down	5.95	17.50**	1.29	17.50**	17.50**	**5.95**	**1.29**	**1.29**	**17.50****	**0.00**	**1.29**	**1.29**	**0.00**	**1.29**
NPTC caregiver posture/action	71.39***	43.07***	45.23***	30.61***	30.61***	39.03***	16.33**	1.91	1.11	23.84***	0.29	18.92**	5.96	22.87***
Caregiver touch R	64.78***	64.01***	58.74***	60.44***	70.75***	33.48***	31.13***	29.12***	42.97***	10.18*	12.01**	19.22***	**1.12**	**5.13**
No caregiver touch R	**15.08****	**2.23**	**10.91***	**15.29****	**8.70***	**2.08**	**1.69**	**7.87***	**8.02***	**1.80**	**1.21**	**2.85**	**1.40**	**0.14**
NPTC touch R	126.05***	120.29***	114.15***	107.18***	110.87***	74.11***	68.79***	56.14***	52.87***	37.18***	26.11***	21.28***	**9.67***	**3.32**
Caregiver touch L	69.41***	54.60***	68.05***	65.64***	72.13***	34.11***	33.47***	37.42***	42.13***	16.67***	15.93***	22.0***	**4.26**	**5.71**
No caregiver touch L	9.55**	3.91	8.24*	12.59**	11.83**	2.39	0.46	7.23*	10.40**	**0.54**	**0.53**	**4.79**	**0.76**	**0.01**
NPTC touch L	129.01***	115.60***	105.59***	98.47***	103.09***	80.15***	56.71***	44.93***	42.35***	37.64***	15.75***	10.87**	**8.99***	**0.55**
Pointing	**4.21**	**1.83**	**3.33**	**3.33**	**2.33**	**1.58**	**0.83**	**3.33**	**2.33**	**0.58**	**0.96**	**1.08**	**0.78**	**0.71**
Reaching	12.28*	8.51	8.85	16.37**	10.23	**5.97**	**1.95**	**7.18**	**6.32**	**1.57**	**0.96**	**2.84**	**1.08**	**0.19**
Gesticulating	12.00*	11.21*	19.83**	11.01	12.47*	**3.79**	**9.72**	**9.02**	**5.97**	**2.97**	**5.26**	**4.92**	**1.32**	**1.39**
Other hand movements	41.79***	35.30***	45.74***	40.83***	51.00***	21.07***	17.77**	18.91**	25.76***	**8.52**	**3.32**	**10.99**	**1.34**	**1.03**
No hand movements	35.71***	34.10***	36.79***	40.14***	34.50***	16.49**	19.22**	19.45**	24.36***	**6.22**	**9.83**	**10.15**	**2.10**	**2.48**
NPTC hand movements	118.76***	91.59***	94.69***	94.09***	95.06***	72.40***	46.91***	42.44***	47.03***	32.55***	13.20*	15.17**	**8.30**	**1.59**
Physical play evident	74.94***	65.42***	56.20***	48.21***	56.69***	38.07***	30.86***	18.03***	27.22***	13.26**	9.60**	11.71**	**1.36**	**3.10**
No physical play	145.45***	137.78***	137.43***	143.89***	137.77***	79.99***	66.00***	70.22***	74.91***	33.82***	22.84***	25.45***	**8.47***	**2.14**
NPTC physical play	64.14***	29.65***	53.04***	44.27***	46.33***	27.83***	20.60***	17.89***	6.65*	22.42***	5.81	0.12	9.51**	2.92
Speech	73.89***	69.69***	78.46***	76.81***	86.86***	35.56***	38.81***	47.63***	51.05***	14.81*	18.30*	25.21***	**3.55**	**5.77**
Musical Sounds	**4.45**	**3.29**	**4.24**	**3.29**	**9.50**	**0.81**	**0.74**	**1.33**	**3.29**	**0.64**	**0.24**	**1.33**	**0.00**	**0.24**
Laugh	**5.35**	**1.43**	**3.50**	**3.67**	**4.11**	**1.02**	**0.55**	**1.87**	**3.27**	**0.14**	**0.21**	**1.55**	**0.01**	**0.19**
Nervous laugh	**0.00**	**1.00**	**1.00**	**1.00**	**1.00**	**0.00**	**1.00**	**1.00**	**1.00**	**0.00**	**1.00**	**1.00**	**0.00**	**1.00**
Vocal Imitation	**4.50**	**2.83**	**4.50**	**3.50**	**1.83**	**2.83**	**2.83**	**3.50**	**0.58**	**2.83**	**2.33**	**0.58**	**2.33**	**0.00**
Bodily sounds	**1.25**	**6.71**	**6.71**	**1.19**	**0.26**	**1.25**	**6.71**	**1.19**	**0.44**	**1.25**	**1.19**	**0.44**	**0.19**	**0.44**
Non-verbal sound	**6.27**	**4.06**	**7.12**	**5.42**	**9.96**	**1.57**	**2.05**	**3.93**	**5.28**	**0.82**	**0.77**	**2.52**	**0.23**	**0.19**
Silent	120.56***	118.37***	108.29***	106.39***	97.30***	77.99***	68.07***	56.65***	52.23***	36.76***	28.72***	19.73**	**9.98**	**5.09**

**p* < 0.05; ***p* < 0.01; ****p* < 0.001. NPTC, not possible to code; BO, body orientation. From top to bottom, behavioural groups shown are: Proximity, Body Orientation, Head Orientation, Visual Attention, Facial Expression, Posture, Touch R, Touch L, Hand Movements, Physical Play, and Vocalisation. Only behaviours that occurred within this population are included. Behaviours have been arranged according to the corresponding behavioural groups. The bold values highlight an example criterion which could be used to identify an appropriate length of time to code for each behaviour, if wanting to investigate transitions between behaviours: i.e. where all chi-squared values for a given slice length have *p* < 0.05. Fully-boldened regions represent the corresponding “cut off”, where *p* < 0.05 for all slices to the right of the line; indicating that the slice length rightward adjacent to the black line would be an appropriate coding timeframe for the given behavioural group. E.g., data within the first rows suggest that coding 4-min of Proximity would be suitable

**Table 18:** Bristol infants (2): chi-squared test on absolute differences between thin slice and full session transition matrices

Behaviour (by category)	Slice (by length)													
	1-min					2-min				3-min			4-min	
	One	Two	Three	Four	Five	OneTwo	TwoThree	ThreeFour	FourFive	OneTwo Three	TwoThree Four	ThreeFour Five	OneTwo ThreeFour	TwoThree FourFive
BO to caregiver 1	115.17***	96.83***	110.54***	101.58***	106.31***	48.76***	51.03***	57.50***	40.17***	25.50***	20.50**	20.41**	**7.96**	**2.19**
BO to caregiver 2	84.83***	67.26***	57.56***	53.30***	52.12***	52.55***	13.37*	8.14	6.33	24.21***	2.28	6.71	6.15	39.82***
BO to sibling	**2.00**	**2.00**	**2.00**	**0.00**	**2.00**	**2.00**	**2.00**	**0.00**	**0.00**	**2.00**	**0.00**	**0.00**	**0.00**	**0.00**
BO to different person/object	0.00	25.00***	25.00***	25.00***	25.00***	0.00	25.0***	25.00***	25.00***	0.00	25.00***	25.00***	0.00	25.00***
BO to object (focus of activity)	70.73***	33.95***	51.68***	33.95***	86.30***	9.60	3.58	20.39**	43.67***	1.42	1.25	29.60***	**14.05***	**3.44**
NPTC infant body orientation	69.10***	53.28***	43.69***	38.42***	42.52***	39.66***	24.44***	3.31	6.26	23.26***	2.96	2.21	**7.01**	**0.53**
Vis-à-vis—infant and caregiver	71.74***	66.13***	75.94***	69.94***	71.56***	35.74***	33.68***	41.26***	36.06***	**14.71***	**14.03**	**17.99***	**4.19**	**2.54**
Slight (30°–90°) aversion right	55.83***	48.36***	35.23***	37.11***	39.85***	39.31***	26.5***	14.23*	13.86	18.49**	8.61	2.74	**4.30**	**0.95**
Slight (30°–90°) aversion left	35.89***	19.96**	21.14**	24.51***	31.88***	19.10**	10.05	10.59	16.18*	**8.42**	**1.47**	**4.11**	**1.23**	**0.10**
Full (90°) aversion right	**5.00**	**0.57**	**5.00**	**1.39**	**5.00**	**0.57**	**0.57**	**1.39**	**1.39**	**0.57**	**0.00**	**1.39**	**0.00**	**0.00**
Full (90°) aversion left	**0.38**	**6.50**	**6.50**	**6.50**	**3.41**	**0.38**	**6.50**	**6.50**	**3.41**	**0.38**	**6.50**	**3.41**	**0.38**	**3.41**
Arch aversion	**2.00**	**2.00**	**2.00**	**2.00**	**0.00**	**2.00**	**2.00**	**2.00**	**0.00**	**2.00**	**2.00**	**0.00**	**2.00**	**0.00**
NPTC head orientation	62.57***	63.85***	62.45***	47.43***	43.94***	34.65***	30.90***	24.58***	9.38	**16.41***	**10.70**	**1.58**	**5.35**	**0.39**
Look at caregiver 1	30.51**	38.77***	38.27***	33.47***	39.83***	**16.98**	**22.43***	**20.09***	**21.25***	**8.33**	**8.41**	**10.38**	**1.41**	**2.70**
Look at caregiver 2	46.36***	35.96***	27.20**	12.77	22.18*	30.52**	19.83*	3.80	2.55	**15.22**	**5.08**	**3.39**	**3.84**	**0.38**
Look at focus object	76.21***	51.43***	53.04***	60.08***	51.41***	42.58***	19.08	29.44**	28.80**	**19.63**	**9.14**	**9.20**	**4.95**	**0.50**
Look at different object	**8.44**	**13.24**	**5.83**	**7.35**	**4.80**	**6.92**	**4.80**	**6.16**	**5.58**	**3.08**	**5.08**	**2.42**	**1.07**	**1.94**
Look at other object	**15.79**	**13.18**	**10.21**	**4.05**	**12.19**	**6.90**	**3.96**	**3.88**	**2.94**	**1.94**	**1.54**	**0.90**	**0.69**	**0.27**
Look at object outside of view	**6.44**	**4.14**	**11.13**	**7.60**	**6.30**	**2.30**	**2.85**	**6.07**	**2.69**	**1.58**	**1.57**	**2.19**	**0.90**	**0.10**
Look at sibling	**17.63**	**9.69**	**1.87**	**3.39**	**23.00***	**12.70**	**1.09**	**0.56**	**3.39**	**1.38**	**2.68**	**0.56**	**0.00**	**2.68**
Look at other person	**17.00**	**17.00**	**0.00**	**17.00**	**17.00**	**17.0**	**0.00**	**0.00**	**17.00**	**0.00**	**0.00**	**0.00**	**0.00**	**0.00**
Look at distraction	**2.00**	**2.00**	**0.00**	**2.00**	**2.00**	**2.00**	**0.00**	**0.00**	**2.00**	**0.00**	**0.00**	**0.00**	**0.00**	**0.00**
No visual attention	**0.00**	**5.00**	**5.00**	**5.00**	**5.00**	**0.00**	**5.00**	**5.00**	**5.00**	**0.00**	**5.00**	**5.00**	**0.00**	**5.00**
NPTC visual attention	103.22***	96.53***	99.41***	105.34***	98.77***	55.52***	48.36***	59.12***	55.60***	**23.77***	**21.81***	**21.02***	**7.91**	**2.57**
Neutral/Alert	45.56***	46.71***	57.54***	50.13***	49.27***	18.99**	30.21***	33.57***	25.89***	10.21	14.77*	17.16**	**2.36**	**5.39**
Positive	31.43***	33.76***	4.26	15.72**	36.73***	25.75***	1.28	3.21	12.47*	**3.67**	**1.73**	**1.64**	**0.32**	**0.71**
Smile	**13.15***	**6.69**	**9.70**	**7.49**	**5.52**	**6.61**	**3.78**	**5.52**	**2.86**	**4.37**	**1.18**	**1.50**	**1.44**	**1.04**
Negative	**0.02**	**9.33**	**9.33**	**1.48**	**7.56**	**0.02**	**9.33**	**1.48**	**2.52**	**0.02**	**1.48**	**2.52**	**0.10**	**2.52**
Disgust	**0.00**	**3.00**	**3.00**	**3.00**	**3.00**	**0.00**	**3.00**	**3.00**	**3.00**	**0.00**	**3.00**	**3.00**	**0.00**	**3.00**
Face not visible	152.66***	145.12***	139.83***	144.11***	137.26***	88.91***	79.24***	78.51***	75.60***	39.46***	35.30***	30.77***	**11.04**	**6.92**
Lie down	**0.00**	**7.00**	**7.00**	**7.00**	**7.00**	**0.00**	**7.00**	**7.00**	**7.00**	**0.00**	**7.00**	**7.00**	**0.00**	**7.00**
Sit on the floor	15.78**	35.00***	7.62	27.78***	35.00***	15.78**	7.62	4.78	27.78***	**0.78**	**4.78**	**4.78**	**0.00**	**4.78**
Sit on an object	193.15***	183.66***	180.84***	180.84***	184.54***	109.56***	100.37***	98.35***	101.05***	48.05***	42.09***	42.56***	**11.54***	**9.65***
Held/in hold	29.69***	3.16	23.62***	73.50***	73.50***	4.03	6.24	23.62***	73.50***	0.00	6.24	23.62***	**0.00**	**6.24**
NPTC infant posture/action	38.51***	34.62***	41.77***	17.28**	29.84***	22.49***	24.06***	1.72	1.92	18.18**	1.39	3.04	**1.78**	**0.79**
Infant touch R	24.35***	32.96***	38.99***	40.76***	36.09***	13.85***	13.50**	25.46***	24.92***	3.47	10.12**	14.31***	**1.60**	**3.76**
No infant touch R	30.34***	32.99***	39.89***	34.23***	32.45***	11.31**	20.89***	21.63***	13.16**	7.37*	10.62**	8.47*	**2.50**	**2.82**
NPTC touch R	142.11***	144.14***	138.66***	142.33***	137.57***	85.51***	79.37***	78.03***	76.09***	37.82***	35.72***	31.65***	10.33**	7.63*
Infant touch L	35.89***	41.53***	35.53***	33.90***	30.06***	21.05***	21.70***	12.73**	10.24**	10.88**	10.79**	0.88	**2.26**	**2.42**
No infant touch L	33.66***	21.24***	17.71***	23.48***	21.11***	14.28***	4.47	6.02*	5.49	**5.20**	**0.03**	**0.24**	**1.04**	**2.99**
NPTC touch L	156.56***	143.23***	146.48***	142.18***	138.51***	90.99***	83.49***	78.65***	68.63***	44.09***	34.89***	29.97***	11.61**	6.36*
Reaching	15.44**	9.70*	18.29**	14.93**	10.84*	**5.33**	**5.75**	**11.37***	**5.77**	**2.86**	**2.55**	**6.16**	**0.90**	**0.60**
Gesticulating	60.34***	57.22***	48.50***	51.03***	39.88***	33.81***	29.15***	24.73***	21.23***	17.75**	5.83	5.78	**3.72**	**0.27**
Other hand movements	50.53***	46.00***	40.92***	35.52***	34.28***	29.77***	25.54***	18.30**	12.95*	18.75***	7.79	4.85	**3.62**	**0.51**
No hand movements	12.15*	14.97**	15.71**	17.75**	20.61***	**4.78**	**6.72**	**9.17**	**10.53***	**1.25**	**4.00**	**4.41**	**0.50**	**1.45**
NPTC hand movements	146.57***	139.95***	132.23***	134.71***	133.09***	82.25***	75.91***	71.98***	71.85***	35.42***	34.95***	33.61***	**9.95***	**7.64**
Physical play evident	174.18***	148.81***	138.16***	137.21***	145.67***	103.31***	76.10***	67.93***	73.08***	44.68***	27.22***	25.71***	10.07**	4.18
No physical play	134.61***	128.80***	118.79***	137.16***	127.88***	75.04***	65.58***	75.58***	71.90***	34.15***	30.73***	29.84***	**9.10***	**5.36**
NPTC physical play	43.62***	41.98***	31.00***	32.51***	14.15***	26.62***	15.64***	4.96	8.47*	10.24**	2.69	1.99	**1.98**	**0.51**
Laughing	**6.00**	**0.00**	**6.00**	**6.00**	**6.00**	**0.00**	**0.00**	**6.00**	**6.00**	**0.00**	**0.00**	**6.00**	**0.00**	**0.00**
Distressed	**11.58**	**15.67***	**14.96***	**3.97**	**3.76**	**9.18**	**8.83**	**3.33**	**2.47**	**6.06**	**1.28**	**1.02**	**1.35**	**0.75**
Non-Distress	24.43***	20.68**	19.19**	16.89**	15.66*	**15.30***	**11.41**	**8.06**	**9.22**	**7.27**	**3.18**	**3.21**	**1.30**	**0.51**
Screaming	69.95***	66.42***	48.66***	12.11	90.00***	59.71***	32.74***	1.64	18.42**	42.11***	1.16	38.84***	10.53	53.79***
Bodily Sounds	50.75***	30.95***	36.13***	42.90***	41.78***	16.45*	13.34*	15.70*	31.06***	**6.04**	**3.17**	**12.51**	**0.35**	**1.24**
Silent/none of the above	115.84***	112.29***	112.27***	114.72***	108.20***	63.93***	54.97***	59.89***	53.90***	27.56***	20.66**	21.3**	**7.60**	**1.94**
NPTC infant vocalisation	**0.00**	**15.00***	**15.00***	**15.00***	**15.00***	**0.00**	**15.00***	**15.00***	**15.00***	**0.00**	**15.00***	**15.00***	**0.00**	**15.00***

**p* < 0.05; ***p* < 0.01; ****p* < 0.001. NPTC, not possible to code; BO, body orientation. From top to bottom, behavioural groups shown are: Body Orientation, Head Orientation, Visual Attention, Facial Expression, Posture, Touch R, Touch L, Hand Movements, Physical Play, and Vocalisation. Only behaviours that occurred within this population are included. Behaviours have been arranged according to the corresponding behavioural groups. The bold values highlight an example criterion which could be used to identify an appropriate length of time to code for each behaviour, if wanting to investigate transitions between behaviours: i.e. where all chi-squared values for a given slice length have *p* < 0.05. Fully-boldened regions represent the corresponding “cut off”, where *p* < 0.05 for all slices to the right of the line; indicating that the slice length rightward adjacent to the black line would be an appropriate coding timeframe for the given behavioural group. E.g., data within the first rows suggest that coding the full 5-min (or longer) of Body Orientation would be suitable

### Stationary Distributions

Here we provide two sets of analyses related to stationary distributions. “Absolute Differences Plot” section includes a plot to show the absolute differences between stationary distributions calculated at each thin slice and full sessions; “Chi-Squared Tables” section provides comprehensive tables containing the results of chi-squared tests on the absolute differences between thin slice and full session stationary distributions.

#### Absolute Differences Plot

See Fig. 3.

#### Chi-Squared Tables

See Tables 19, 20, 21, 22, 23 and 24.

**Table 19:** Cardiff mums: chi-squared test on absolute differences between thin slice and full session stationary distributions

Behaviour category	Slice (by length)													
	1-min					2-min				3-min			4-min	
	One	Two	Three	Four	Five	OneTwo	TwoThree	ThreeFour	FourFive	OneTwo Three	TwoThree Four	ThreeFour Five	OneTwo ThreeFour	TwoThree FourFive
Proximity	21.50***	7.70	5.04	2.31	4.97	**2.35**	**8.08***	**4.24**	**2.05**	**2.01**	**1.44**	**0.73**	**0.60**	**0.87**
Body orientation	14.18**	132.8***	2.50	7.99*	3.28	68.22***	6.54	3.23	1.81	**1.03**	**6.79**	**1.12**	**3.04**	**1.40**
Visual attention	13.50	3.95	3.14	19.53**	28.25***	**4.45**	**1.89**	**1.86**	**2.20**	**0.88**	**0.92**	**0.57**	**0.95**	**0.27**
Posture	49.06***	19.69**	105.75***	19.65**	9.37	14.59*	110.73***	19.47**	6.10	106.01***	18.29*	4.00	**11.1**	**3.30**
Touch right hand	**4.66**	**3.39**	**2.82**	**1.11**	**2.10**	**0.88**	**1.27**	**0.62**	**0.43**	**0.25**	**0.55**	**0.43**	**0.02**	**0.46**
Touch left hand	21.49***	28.90***	7.08	4.68	6.95	21.24***	5.90	3.20	3.95	**2.62**	**2.07**	**3.66**	**0.40**	**2.74**
Physical play	37.79***	0.73	10.20**	3.22	13.2**	**2.90**	**2.68**	**0.81**	**2.12**	**3.99**	**0.80**	**1.37**	**0.20**	**1.20**
Vocalisation	3.35	43.67***	6.62	0.87	6.12	43.17***	1.63	1.37	2.27	**1.01**	**0.41**	**0.56**	**0.41**	**0.22**

**p* < 0.05; ***p* < 0.01; ****p* < 0.001. Only behavioural groups that were coded within this population are included. The bold values highlight an example criterion which could be used to identify an appropriate length of time to code for each behaviour, if wanting to investigate stationary distributions: i.e. where all chi-squared values for a given slice length have *p* < 0.05. The furthest left bold region in a row indicates that the corresponding slice length would be an appropriate coding timeframe for the given behavioural group. E.g., the first row suggests that coding 2 min of Proximity would be suitable for understanding the associated stationary distribution

**Table 20:** Cardiff infants: chi-squared test on absolute differences between thin slice and full session stationary distributions

Behaviour category	Slice (by length)													
	1-min					2-min				3-min			4-min	
	One	Two	Three	Four	Five	OneTwo	TwoThree	ThreeFour	FourFive	OneTwo Three	TwoThree Four	ThreeFour Five	OneTwo ThreeFour	TwoThree FourFive
Body orientation	10.55*	24.35***	9.38*	24.66***	2.01	14.03**	13.34**	6.78	2.39	**3.31**	**3.18**	**1.67**	**1.02**	**1.45**
Visual attention	12.55	2.73	113.81***	6.63	11.19	5.01	102.61***	5.43	1.72	99.14***	2.48	0.49	**0.74**	**0.29**
Posture	1207.74***	90.07***	52.63***	74.25***	10.03	137.62***	30.98***	49.16***	18.63*	22.02*	53.65***	14.43	54.84***	2.85
Touch right hand	26.57***	16.46**	26.86***	25.54***	7.93	14.93**	24.35***	23.13***	1.68	23.45***	22.59***	1.03	22.66***	0.06
Touch left hand	17.91**	1.94	31.69***	1.97	2.70	**6.89**	**8.14**	**12.03***	**0.77**	**9.37**	**8.04**	**0.37**	**8.98**	**0.27**
Physical play	5.90	17.32***	5.50	8.46*	8.20*	7.90*	9.70**	5.47	8.15*	**7.77***	**0.43**	**4.94**	**0.50**	**0.15**
Vocalisation	4.53	5.06	152.36***	2.91	3.90	1.95	170.87***	1.59	2.10	174.01***	0.48	0.92	**0.28**	**0.25**

**p* < 0.05; ***p* < 0.01; ****p* < 0.001. Only behavioural groups that were coded within this population are included. The bold values highlight an example criterion which could be used to identify an appropriate length of time to code for each behaviour, if wanting to investigate stationary distributions: i.e. where all chi-squared values for a given slice length have *p* < 0.05. The furthest left bold region in a row indicates that the corresponding slice length would be an appropriate coding timeframe for the given behavioural group. E.g., the first row suggests that coding 3 min of Body Orientation would be suitable for understanding the associated stationary distribution

**Table 21:** Bristol mums: chi-squared test on absolute differences between thin slice and full session stationary distributions

Behaviour category	Slice (by length)													
	1-min					2-min				3-min			4-min	
	One	Two	Three	Four	Five	OneTwo	TwoThree	ThreeFour	FourFive	OneTwo Three	TwoThree Four	ThreeFour Five	OneTwo ThreeFour	TwoThree FourFive
Proximity	9.67*	21.04***	87.07***	42.34***	79.75***	16.59***	52.72***	30.95***	61.10***	7.44	0.56	32.0***	**1.21**	**0.18**
Body orientation	16.79***	30.33***	71.39***	7.34	9.01*	6.80	13.29**	0.84	6.74	**7.58**	**2.21**	**1.86**	**0.65**	**0.27**
Head orientation	26.03***	759.77***	179.24***	264.54***	10.62*	754.37***	139.4***	6.66	3.51	**141.22*****	**1.02**	**2.70**	**0.43**	**0.64**
Visual attention	45.43***	31.15***	46.62***	31.83***	36.21***	52.72***	5.67	30.17***	28.86**	5.92	2.74	25.69**	**2.31**	**1.15**
Facial expression	580.03***	4.66	8.82	3.90	164.04***	**2.86**	**2.08**	**1.47**	**4.57**	**1.28**	**1.24**	**1.56**	**0.30**	**1.13**
Posture	36.91***	4.62	0.55	4.62	0.55	**0.90**	**0.88**	**0.88**	**0.88**	**0.40**	**0.24**	**0.39**	**0.03**	**0.03**
Touch right hand	0.31	2.32	15.03***	3.56	32.94***	**0.84**	**0.91**	**4.40**	**1.41**	**0.71**	**1.06**	**0.43**	**0.66**	**0.01**
Touch left hand	2.55	22.43***	8.93*	4.05	30.53***	**2.63**	**1.20**	**6.86***	**5.54**	**1.01**	**1.00**	**3.64**	**0.94**	**0.06**
Hand movements	9.84	62.87***	13.23*	14.65*	93.94***	**9.84**	**5.19**	**8.21**	**9.51**	**2.04**	**1.80**	**12.22***	**1.40**	**12.54***
Physical play	3.57	3.16	9.37**	0.16	1.07	**3.36**	**0.41**	**1.28**	**0.05**	**0.04**	**0.06**	**1.23**	**0.06**	**0.19**
Vocalisation	64.54***	121.63***	63.60***	79.19***	12.79	57.67***	82.82***	65.45***	1.91	67.56***	75.49***	1.40	67.13***	0.25

**p* < 0.05; ***p* < 0.01; ****p* < 0.001. Only behavioural groups that were coded within this population are included. The bold values highlight an example criterion which could be used to identify an appropriate length of time to code for each behaviour, if wanting to investigate stationary distributions: i.e. where all chi-squared values for a given slice length have *p* < 0.05. The furthest left bold region in a row indicates that the corresponding slice length would be an appropriate coding timeframe for the given behavioural group. E.g., the first row suggests that coding 4 min of Proximity would be suitable for understanding the associated stationary distribution

**Table 22:** Bristol infants (1): chi-squared test on absolute differences between thin slice and full session stationary distributions

Behaviour category	Slice (by length)													
	1-min					2-min				3-min			4-min	
	One	Two	Three	Four	Five	OneTwo	TwoThree	ThreeFour	FourFive	OneTwo Three	TwoThree Four	ThreeFour Five	OneTwo ThreeFour	TwoThree FourFive
Body orientation	67.84***	28.10***	25.62***	39.22***	14.80**	43.18***	18.97***	31.90***	10.74*	31.24***	19.75***	9.29*	28.93***	1.66
Head orientation	59.30***	35.19***	14.87*	34.71***	39.56***	29.94***	7.10	11.61	46.04***	8.64	4.08	33.17***	**3.73**	**2.89**
Visual attention	7.66	11.39	42.89***	15.10	97.74***	2.94	9.50	6.05	26.67***	2.87	2.52	21.26**	**0.59**	**0.51**
Facial expression	13.77*	8.32	25.25***	9.08	110.26***	**2.20**	**11.70**	**1.37**	**14.17***	**3.56**	**1.09**	**11.05**	**0.44**	**5.05**
Posture	331.99***	11.56	199.35***	13.52	13.52	11.84	126.42***	38.81***	13.52	126.20***	12.62	19.77**	**0.72**	**4.78**
Touch right hand	19.04***	38.87***	3.62	1.38	23.69***	**7.67***	**5.64**	**2.37**	**8.25***	**3.84**	**0.57**	**4.33**	**1.26**	**0.91**
Touch left hand	42.02***	24.91***	14.48***	2.21	4.60	30.46***	21.06***	3.21	3.33	20.78***	2.04	3.70	**0.30**	**2.68**
Hand movements	66.69***	11.54	6.24	29.49***	8.66	**5.52**	**4.92**	**5.08**	**5.98**	**1.87**	**3.21**	**2.11**	**0.44**	**1.64**
Physical play	**2.11**	**0.36**	**3.14**	**2.38**	**7.21***	**0.48**	**0.04**	**2.62**	**3.49**	**0.16**	**0.05**	**3.33**	**0.15**	**0.16**
Vocalisation	17.51*	9.50	16.43*	17.28*	33.17***	12.87	11.33	24.42***	12.21	**10.97**	**1.40**	**2.51**	**1.60**	**0.64**

**p* < 0.05; ***p* < 0.01; ****p* < 0.001. Only behavioural groups that were coded within this population are included. The bold values highlight an example criterion which could be used to identify an appropriate length of time to code for each behaviour, if wanting to investigate stationary distributions: i.e. where all chi-squared values for a given slice length have *p* < 0.05. The furthest left bold region in a row indicates that the corresponding slice length would be an appropriate coding timeframe for the given behavioural group. E.g., the first row suggests that coding 5 min (or longer) of Body Orientation would be suitable for understanding the associated stationary distribution

**Table 23:** Bristol dads: chi-squared test on absolute differences between thin slice and full session stationary distributions

Behaviour category	Slice (by length)													
	1-min					2-min				3-min			4-min	
	One	Two	Three	Four	Five	OneTwo	TwoThree	ThreeFour	FourFive	OneTwo Three	TwoThree Four	ThreeFour Five	OneTwo ThreeFour	TwoThree FourFive
Proximity	9.14*	44.92***	1.04	4.55	31.09***	22.07***	2.20	2.74	0.76	**0.25**	**2.62**	**0.18**	**0.88**	**0.09**
Body orientation	17.10**	5.95	208.12***	14.81**	13.97**	3.47	55.47***	7.85	10.46*	65.15***	5.47	8.59	**1.35**	**3.42**
Head orientation	29.81***	22.65***	183.33***	35.51***	8.27	19.63**	59.53***	24.53***	10.71	0.93	19.89**	8.05	**16.24***	**10.3**
Visual attention	41.98***	12.86	69.40***	17.61*	7.75	**21.33***	**13.11**	**4.49**	**11.56**	**1.95**	**2.62**	**6.57**	**0.88**	**7.88**
Facial expression	31.53***	25.18***	24.09**	11.39	41.71***	26.26***	13.99	6.64	21.76**	**10.55**	**2.83**	**2.89**	**1.09**	**1.13**
Posture	**2.19**	**6.38**	**1.02**	**1.02**	**8.84**	**4.46**	**0.63**	**1.02**	**0.47**	**0.45**	**0.75**	**0.25**	**0.56**	**0.22**
Touch right hand	2.27	3.61	10.99**	7.69*	2.98	**1.04**	**1.91**	**1.23**	**4.25**	**2.03**	**1.55**	**0.68**	**0.79**	**0.63**
Touch left hand	17.78***	13.20**	3.67	24.40***	0.79	9.39**	0.30	5.44	6.72*	**3.29**	**0.8**	**4.36**	**0.20**	**1.15**
Hand movements	28.99***	4.56	6.96	7.70	50.55***	4.74	1.41	3.68	44.02***	2.28	1.20	40.38***	**0.58**	**13.56***
Physical play	13.48**	13.09**	5.70	2.57	0.88	13.16**	9.54**	0.32	0.15	10.92**	0.07	0.18	**0.00**	**0.06**
Vocalisation	10.24	161.85***	4.83	4.60	35.72***	153.11***	7.47	3.00	7.48	**3.84**	**2.88**	**3.87**	**1.58**	**0.54**

**p* < 0.05; ***p* < 0.01; ****p* < 0.001. Only behavioural groups that were coded within this population are included. The bold values highlight an example criterion which could be used to identify an appropriate length of time to code for each behaviour, if wanting to investigate stationary distributions: i.e. where all chi-squared values for a given slice length have *p* < 0.05. The furthest left bold region in a row indicates that the corresponding slice length would be an appropriate coding timeframe for the given behavioural group. E.g., the first row suggests that coding 3 min of Proximity would be suitable for understanding the associated stationary distribution

**Table 24:** Bristol infants (2): chi-squared test on absolute differences between thin slice and full session stationary distributions

Behaviour category	Slice (by length)													
	1-min					2-min				3-min			4-min	
	One	Two	Three	Four	Five	OneTwo	TwoThree	ThreeFour	FourFive	OneTwo Three	TwoThree Four	ThreeFour Five	OneTwo ThreeFour	TwoThree FourFive
Body orientation	3.58	7.46	10.87*	24.72***	6.98	5.48	10.95*	25.56***	9.03	9.55*	23.92***	9.40	22.18***	10.43*
Head orientation	58.89***	15.38*	21.22**	14.17*	22.71**	**17.64***	**7.27**	**9.59**	**1.44**	**1.06**	**6.75**	**3.55**	**1.54**	**2.45**
Visual attention	34.51***	20.28*	36.54***	20.24*	12.73	**8.93**	**8.97**	**8.95**	**10.4**	**4.61**	**4.27**	**4.30**	**0.75**	**3.10**
Facial expression	66.01***	11.16*	11.62*	67.53***	164.01***	9.22	3.22	71.12***	1.93	0.67	47.42***	3.9	39.36***	1.59
Posture	121.48***	41.19***	15.15**	14.15**	13.21*	63.80***	12.98*	16.34**	9.75*	15.55**	14.52**	8.84	15.79**	9.33
Touch right hand	26.38***	1.03	0.72	1.01	28.53***	**2.52**	**0.03**	**0.11**	**1.13**	**0.66**	**0.12**	**0.68**	**0.59**	**0.21**
Touch left hand	36.27***	3.77	7.48*	1.40	5.72	**9.19***	**4.70**	**3.62**	**3.77**	**1.75**	**3.22**	**4.91**	**0.54**	**4.25**
Hand movements	13.80**	53.31***	3.06	9.82*	4.09	**7.24**	**4.75**	**5.54**	**3.38**	**1.92**	**1.50**	**3.13**	**0.27**	**0.86**
Physical play	2.00	31.63***	1.35	0.77	0.37	21.81***	0.81	1.05	0.10	**0.03**	**0.73**	**0.21**	**0.08**	**0.16**
Vocalisation	23.10***	97.79***	57.45***	34.77***	28.88***	41.10***	54.55***	40.78***	7.67	40.45***	42.26***	1.48	35.03***	0.48

**p* < 0.05; ***p* < 0.01; ****p* < 0.001. Only behavioural groups that were coded within this population are included. The bold values highlight an example criterion which could be used to identify an appropriate length of time to code for each behaviour, if wanting to investigate stationary distributions: i.e. where all chi-squared values for a given slice length have *p* < 0.05. The furthest left bold region in a row indicates that the corresponding slice length would be an appropriate coding timeframe for the given behavioural group. E.g., the first row suggests that coding 5 min (or longer) of Body Orientation would be suitable for understanding the associated stationary distribution

### Additional Non-overlapping Slices Analyses

Here, we provide some additional analyses to illustrate how future work could be adapted to account for non-overlapping slices. We have explored this as we understand that our correlations may be inflated on account of the thin slices themselves being contained within the full sessions.

As examples, we calculated Pearson correlations between three sets of non-overlapping slices: *One* and *TwoThreeFourFive*, *Two* and *Four*, and *OneTwo* and *FourFive.* These correlations are presented below in Tables 25, 26 and 27.

**Table 25:** Pearson correlations between slice *One* and slice *TwoThreeFourFive* for each population

Behavioural category	Population					
	Cardiff mums	Cardiff infants	Bristol mums	Bristol infants (1)	Bristol dads	Bristol infants (2)
Proximity	0.38		0.54		0.58	
Body orientation	0.30	0.54	0.88	0.79	0.72	0.32
Head orientation			0.78	0.88	0.80	0.67
Visual attention	0.55	0.77	0.30	0.69	0.74	0.87
Facial expression			0.89	0.75	0.73	0.92
Posture	0.20	0.84	0.30	0.69	0.23	0.34
Touch right hand	0.66	0.64	0.80	0.51	0.93	0.79
Touch left hand	0.60	0.67	0.83	0.74	0.78	0.60
Hand movements			0.79	0.49	0.94	0.75
Physical play	0.13	0.46	0.84	0.56	0.97	0.93
Vocalisation	0.77	0.78	0.80	0.82	0.63	0.57

A blank value indicates where data was not coded for that specific sample and population (e.g., proximity was not coded for any infant samples)

**Table 26:** Pearson correlations between slice *Two* and slice *Four* for each population

Behavioural category	Population					
	Cardiff mums	Cardiff infants	Bristol mums	Bristol infants (1)	Bristol dads	Bristol infants (2)
Proximity	0.37		0.78		0.92	
Body orientation	0.42	0.44	0.79	0.60	0.85	0.76
Head orientation			0.74	0.66	0.76	0.78
Visual attention	0.80	0.46	0.36	0.59	0.36	0.81
Facial expression			0.70	0.49	0.66	0.90
Posture	0.05	0.36	0.95	0.94	0.93	0.27
Touch right hand	0.48	0.50	0.80	0.62	0.78	0.90
Touch left hand	0.43	0.58	0.64	0.89	0.88	0.89
Hand movements			0.96	0.63	0.95	0.87
Physical play	0.14	0.37	0.62	0.85	0.66	0.99
Vocalisation	0.55	0.58	0.68	0.87	0.78	0.88

A blank value indicates where data was not coded for that specific sample and population (e.g., proximity was not coded for any infant samples)

**Table 27:** Pearson correlations between slice *OneTwo* and slice *FourFive* for each population

Behavioural category	Population					
	Cardiff mums	Cardiff infants	Bristol mums	Bristol infants (1)	Bristol dads	Bristol infants (2)
Proximity	0.31		0.81		0.78	
Body orientation	0.31	0.53	0.94	0.72	0.92	0.60
Head orientation			0.82	0.74	0.87	0.82
Visual attention	0.62	0.49	0.29	0.67	0.86	0.90
Facial expression			0.68	0.32	0.82	0.91
Posture	0.04	0.58	0.60	0.87	0.69	0.50
Touch right hand	0.60	0.59	0.78	0.41	0.83	0.92
Touch left hand	0.41	0.52	0.97	0.81	0.89	0.79
Hand movements			0.88	0.56	0.92	0.85
Physical play	0.03	0.25	0.66	0.61	0.89	0.98
Vocalisation	0.68	0.50	0.74	0.91	0.44	0.83

A blank value indicates where data was not coded for that specific sample and population (e.g., proximity was not coded for any infant samples)

These correlations reveal many of the same patterns that emerged within the analysis presented in the main work. For example, the correlations between behaviours in the Cardiff dataset are almost always lower than their equivalents within the Bristol datasets. Additionally, we observe that the behaviours with lowest correlations in the main work continue to show lowest correlations here (e.g., Proximity, Posture); the same is generally true for the highest correlation behaviours (e.g., Vocalisation, Touch).

What is interesting, however, is that many of the correlations presented here are “strong” or “very strong” (e.g., Bristol Dads Body Orientation, Head Orientation, and Visual Attention in Table 27). So while it is true that our correlations may be inflated slightly on account of the thin slice itself being contained within the full session, we note that correlations between non-adjacent slices (of varying lengths) can also be strong, dependent on behaviour. This suggests that thin slices can also be representative of other portions of the interaction.

## Code Availability

Available at: https://github.com/RomanaBurgess/Thin_Slice_Sampling.
